# Amphiphilic stilbene derivatives attenuate the neurotoxicity of soluble Aβ_42_ oligomers by controlling their interactions with cell membranes†

**DOI:** 10.1039/d2sc02654f

**Published:** 2022-10-19

**Authors:** Zhengxin Yu, Weijie Guo, Shrey Patel, Hong-Jun Cho, Liang Sun, Liviu M. Mirica

**Affiliations:** Department of Chemistry, Beckman Institute for Advanced Science and Technology, The Neuroscience Program, University of Illinois at Urbana-Champaign 600 S. Mathews Avenue Urbana Illinois 61801 USA mirica@illinois.edu; Department of Biochemistry, University of Illinois at Urbana-Champaign 600 S. Mathews Avenue Urbana Illinois 61801 USA; Hope Center for Neurological Disorders, Washington University School of Medicine St. Louis MO 63110 USA

## Abstract

The misfolded proteins or polypeptides commonly observed in neurodegenerative diseases, including Alzheimer's disease (AD), are promising drug targets for developing therapeutic agents. To target the amyloid-β (Aβ) peptide plaques and oligomers, the hallmarks of AD, we have developed twelve amphiphilic small molecules with different hydrophobic and hydrophilic fragments. *In vitro* fluorescence binding assays demonstrate that these amphiphilic compounds show high binding affinity to both Aβ plaques and oligomers, and six of them exhibit selective binding toward Aβ oligomers. These amphiphilic compounds can also label the Aβ species in the brain sections of transgenic AD mice, as shown by immunostaining with an Aβ antibody. Molecular docking studies were performed to obtain structure–affinity relationships. To our delight, four amphiphilic compounds can alleviate the Cu^2+^–Aβ induced toxicity in cell viability assays. In addition, confocal fluorescence imaging studies provide evidence that two compounds, ZY-15-MT and ZY-15-OMe, can disrupt the interactions between Aβ oligomers and human neuroblastoma SH-SY5Y cell membranes. Overall, these studies strongly suggest that developing compounds with amphiphilic properties that target Aβ oligomers and modulate the Aβ oligomer–cell membrane interactions can be an effective strategy for the development of small molecule AD therapeutics.

## Abstract 2

Amphiphilic compounds with selectivity towards soluble Aβ_42_ oligomers were developed. Cell imaging studies show the compounds can reduce the interactions between Aβ_42_ oligomers and SH-SY5Y cell membranes, both in the presence and absence of Cu.

## Graphical abstract

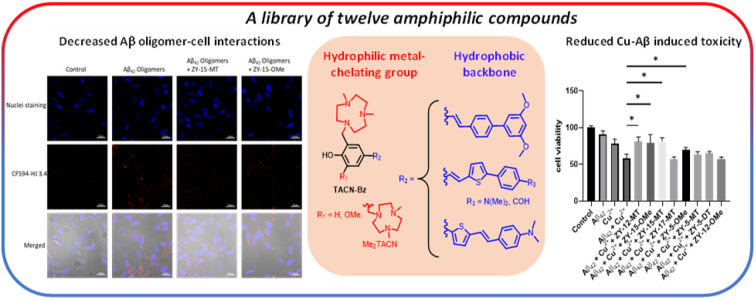

## Introduction

The aggregation of misfolded proteins is commonly observed in different neurodegenerative diseases, including the amyloid-β (Aβ) peptides and tau proteins in Alzheimer's disease (AD), or α-synuclein in Parkinson's disease.^1–3^ AD is the most prevalent neurodegenerative disease and affects more than 44 million people worldwide, yet there is still a lack of effective treatments.^4,5^ Although the insoluble amyloid plaques have been considered the main hallmark of AD over the past century, recently the soluble Aβ oligomers were found to be the most neurotoxic species that directly affect synapse loss and neuronal injury.^6–9^ Since Aβ oligomer species appear in the early stages of the disease and are the cause of continuous synaptic damage, they have become attractive targets for AD drug development.^10,11^ Therefore, numerous strategies have been developed to target soluble Aβ oligomers by using various antibodies,^12,13^ polypeptides,^14,15^ natural products,^16–20^ as well as small molecules. Small molecules utilized as therapeutic agents for central nervous system (CNS) disorders have advantages over biologics, including low molecular weight for higher blood–brain barrier (BBB) permeability and simpler structures for easy access at low cost.^21^ However, due to the lack of a molecular-level understanding of Aβ oligomers' structures, there is no effective way to design small molecules with high and selective Aβ oligomers binding affinity and to modulate their neurotoxicity.

**Fig. 1:**
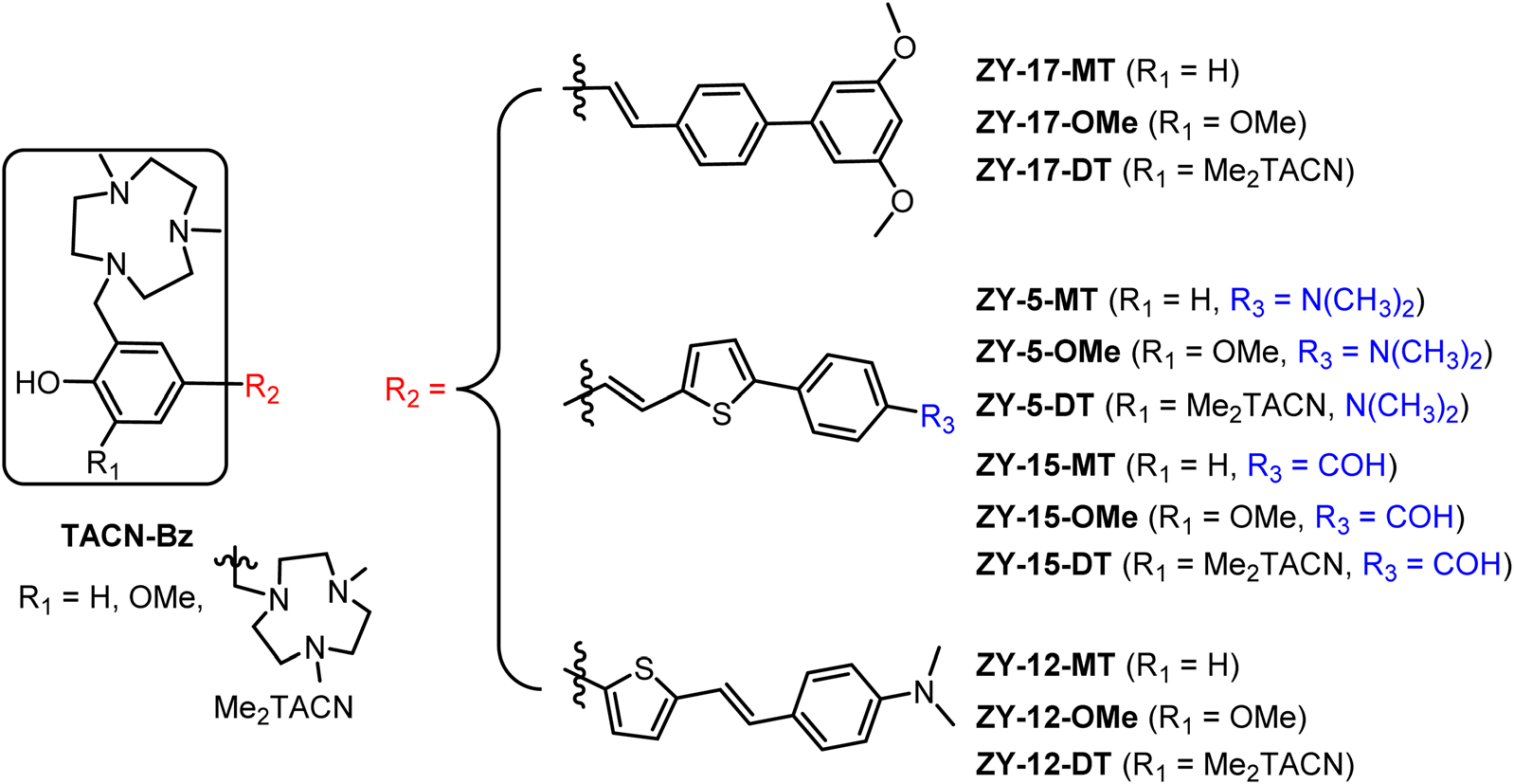
Molecular structures of the twelve amphiphilic compounds.

With the aim of developing therapeutic agents to alleviate the neurotoxicity of Aβ oligomers, we report herein an effective strategy to develop twelve amphiphilic compounds with different amphiphilicity by linking various hydrophobic stilbene derivatives to the hydrophilic triazamacrocycle (Me_2_TACN, Fig. 1). The binding affinity of the amphiphilic compounds was measured by *in vitro* fluorescence saturation assays. These compounds show high binding affinities to both Aβ fibrils and oligomers in the low micromolar to high nanomolar range. More importantly, six of the compounds exhibit selective binding affinity toward Aβ oligomers. Molecular docking studies were performed to extract structure–affinity relationships need for the design of second-generation compounds. Immunostaining assays with an Aβ antibody HJ 3.4 confirms that our compounds are able to label *ex vivo* Aβ species in the brain sections of transgenic AD mice. Cell toxicity studies demonstrate that four compounds could rescue mouse neuroblastoma N2a cells from Cu^2+^–Aβ induced toxicity. Finally, the ZY-15-MT and ZY-15-OMe compounds were found to decrease the interaction between Aβ oligomers and human neuroblastoma SH-SY5Y cells as shown *via* confocal microscopy studies. Overall, these findings provide lead compounds for future optimization and demonstrate an effective strategy for small molecule development.

## Results and discussion

### Design and synthesis of the amphiphilic compounds

Inspired by the amphiphilic nature of the Aβ peptide, we proposed that developing compounds with amphiphilic properties targeting Aβ species could effectively treat AD. Recently, peptidomimetic-based amphiphilic compounds were shown to inhibit Aβ fibrillation process and attenuate Aβ cytotoxicity in both neuroblastoma N2a and human neuroblastoma SH-SY5Y cells.^22^ Moreover, our group successfully developed an amphiphilic small molecule, LS-4, that can serve as a therapeutic and imaging agent for Aβ oligomers in AD.^23^ LS-4 was synthesized by attaching a hydrophilic azamacrocycle, 2,4-dimethyl-1,4,7-triazacyclononane (Me_2_HTACN), to a hydrophobic distyryl stilbene derivative. Interestingly, because of the incorporation of the hydrophilic TACN fragment, the binding affinity of LS-4 toward both Aβ fibrils and oligomers increased dramatically *vs.* the Pre-LS-4 precursor, which does not contain the Me_2_TACN group. Although LS-4 showed high binding affinity to Aβ fibrils (*K*_d_ = 58 ± 15 nM) and Aβ oligomers (*K*_d_ = 50 ± 9 nM), there is no selectivity of LS-4 toward Aβ oligomers. Considering the higher neurotoxicity of Aβ oligomers and their appearance in the early stages of AD, it is beneficial to develop compounds with high affinity and selectivity toward Aβ oligomers. For this purpose, we designed a series of compounds with different amphiphilicity by adding different hydrophobic aromatic ring systems and changing the number of hydrophilic Me_2_TACN groups attached to the (hetero)aromatic conjugated fragments. More specifically, we designed the TACN-Bz component of the compounds with (1) a hydroxyl group with one Me_2_TACN (where R1 = H, yielding the ZY-#-MT series), (2) a methoxy group with one Me_2_TACN (where R1 = OMe, yielding the ZY-#-OMe series), and (3) two Me_2_TACN groups (where R1 = Me_2_TACN, giving ZY-#-DT series). In the ZY-#-OMe series, the methoxy group *ortho* to the hydroxyl group was introduced as its interaction with Aβ oligomers was reported previously.^24^ For the R2 component of the molecules, we incorporated different aromatic ring systems (thiophene–benzene or benzene–benzene) with different substituents for their potential hydrophobic π–π interactions with the Aβ species. Based on this design approach, twelve compounds were designed for structure–activity relationships (SAR) studies and further analysis.

**Scheme 1:**
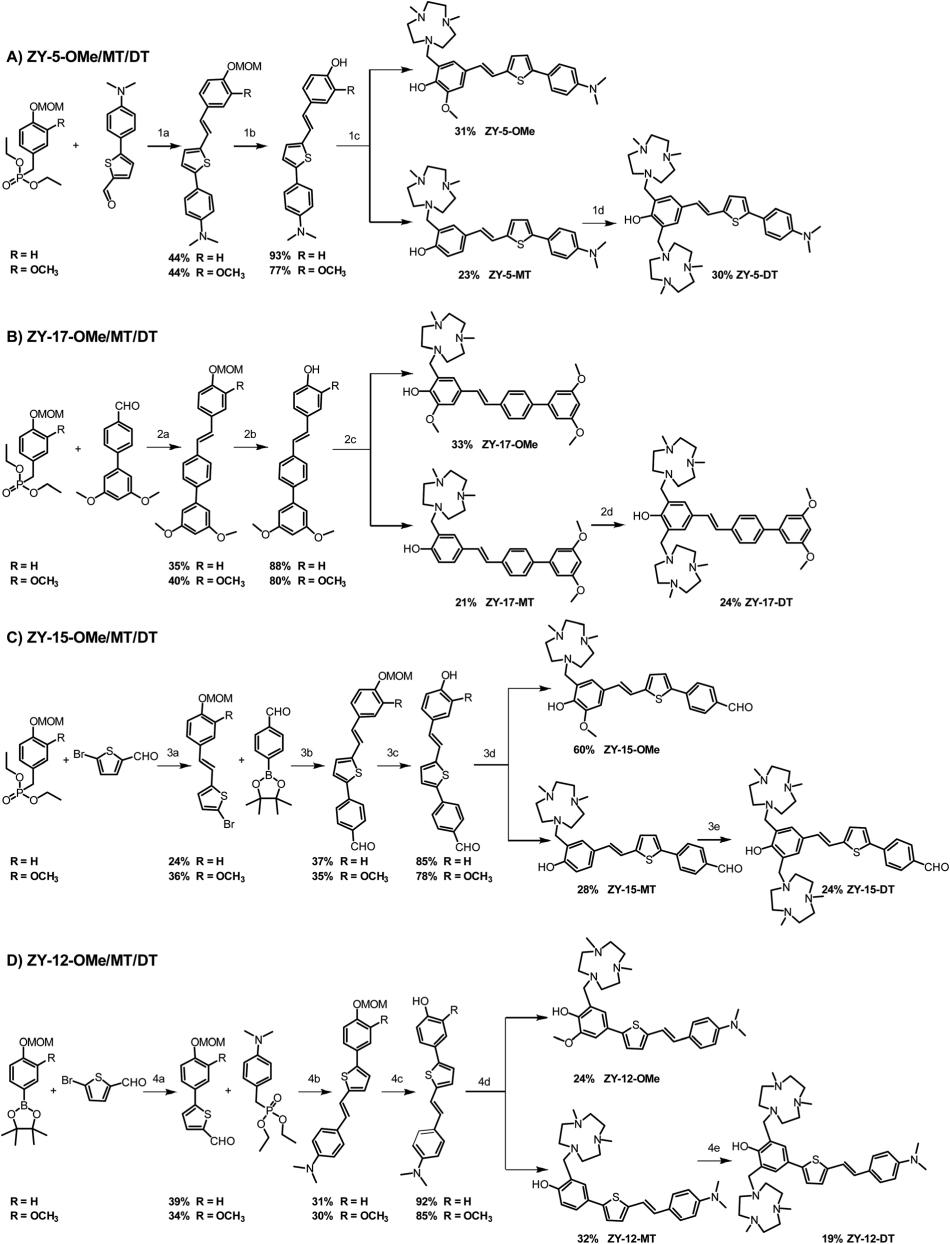
Synthetic route for the amphiphilic compounds. (A) Synthesis for ZY-5-OMe/MT/DT. (B) Synthesis for ZY-17-OMe/MT/DT. (C) Synthesis for ZY-15-OMe/MT/DT. (D) Synthesis for ZY-12-OMe/MT/DT. Reagents and conditions: (1a) KOtBu, DMF, rt, overnight; (1b) HCl, CH_2_Cl_2_, MeOH, rt, 12 h; (1c) (CH_2_O)_*n*_, Me_2_HTACN, MeCN, reflux, 16 h; (1d) (CH_2_O)_*n*_, Me_2_HTACN, MeCN, reflux, 24 h; (2a) NaOMe, DMF, rt; (2b) HCl, CH_2_Cl_2_, MeOH, rt, overnight; (2c) (CH_2_O)_*n*_, Me_2_HTACN, MeCN, reflux; (2d) (CH_2_O)_*n*_, Me_2_HTACN, MeCN, reflux, 24 h; (3a) NaOMe, DMF, r, 24 h; (3b) Pd(PPh_3_)_4_, K_2_CO_3_, toluene, ethanol, reflux; (3c) HCl, CH_2_Cl_2_, MeOH, rt, 12 h; (3d) (CH_2_O)_*n*_, Me_2_HTACN, MeCN, reflux, overnight; (3e) (CH_2_O)_*n*_, Me_2_HTACN, MeCN, reflux 24 h; (4a) Pd(PPh_3_)_4_, K_2_CO_3_, toluene, ethanol, reflux, 6 h; (4b) HCl, CH_2_Cl_2_, MeOH, rt, overnight; (4c) (CH_2_O)_*n*_, Me_2_HTACN, MeCN, reflux, 16 h; (4d) (CH_2_O)_*n*_, Me_2_HTACN, MeCN, reflux, 24 h.

These 12 compounds were synthesized following a streamlined synthetic process (Scheme 1).^25^ For ZY-5-OMe/MT/DT and ZY-17-OMe/MT/DT compounds (Scheme 1A and B), the Horner–Wadsworth–Emmons (HWE) olefination reaction was used to form (*E*)-selective olefins between the MOM-protected benzyl diethyl phosphonate and different aldehyde groups under basic conditions. The free hydroxyl group was obtained after MOM deprotection using HCl, followed by a Mannich reaction with paraformaldehyde and Me_2_HTACN to generate the corresponding final “OMe” or “MT” compounds.^26,27^ Interestingly, an additional Mannich reaction on the “MT” compounds can be performed to add a second Me_2_TACN group on the unsubstituted *ortho* position, using paraformaldehyde and Me_2_HTACN to obtain the “DT” compounds. For ZY-15-OMe/MT/DT (Scheme 1C), due to the presence of an aldehyde group on the R2 component of the compound, we modified the synthesis by first forming the double bond *via* the HWE olefination reaction, followed by the addition of the benzaldehyde by using a Suzuki coupling reaction. In order to study the effect of the position of the double bond relative to the heterocycles, we also synthesized ZY-12-OMe/MT/DT (Scheme 1D) with similar structures to ZY-5-OME/MT/DT except for the position of the double bonds. Due to the structural differences, the synthetic steps for ZY-12-OMe/MT/DT were modified accordingly. First, a Suzuki coupling was performed between MOM-protected boron pinacol esters and thiophene bromide, followed by the HWE olefination reaction to synthesize the second intermediate containing the double bond. The MOM deprotection and the Mannich reaction were performed to obtain the final “OMe” or “MT” compounds. Similar to the synthesis of other “DT” compounds, ZY-12-DT was synthesized through an additional Mannich reaction.

### Fluorescence binding assays

**Fig. 2:**
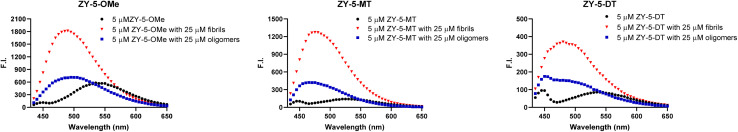
Fluorescence turn-on effects with Aβ_42_ oligomers and fibrils. Left ZY-5-OMe, middle ZY-5-MT right ZY-5-DT. Black: compound only; Blue: compound + Aβ_42_ oligomers; Red: compound + Aβ_42_ fibrils; [compound] = 5 μM; [Aβ_42_ oligomers] = 25 μM; [Aβ_42_ fibrils] = 25 μM.

To investigate whether these amphiphilic compounds can interact with different Aβ species, we recorded the fluorescence intensity changes in the absence and presence of Aβ_42_ fibrils and oligomers, respectively, which were prepared as previously reported.^28^ These compounds, such as the ZY-5 series exhibit a fluorescence turn-on effect when binding to Aβ species, along with a dramatic blue shift of the emission wavelength (Fig. 2 and S3†). The enhanced fluorescence intensity and blue shift are possibly due to the restriction in a rotation of the aromatic rings and the changes of hydrophobicity at the Aβ binding sites. In addition, the compounds also showed excellent selectivity over human serum albumin (HSA), and most compounds showed minimal enhanced fluorescence intensity in the presence of HSA (Fig. S4†). Surprisingly, the ZY-17 series of compounds had a significant turn-on effect towards HSA. The compounds ZY-17-OMe and ZY-17-DT showed an even higher fluorescence intensity increase in the presence of HSA than for Aβ_42_ fibrils (Fig. S4†). Comparing the ZY-17 series with the other series of compounds, we believe that the thiophene ring in the stilbene-like binding scaffold is essential for the selectively toward Aβ species, while the more hydrophobic tri-benzene rings might lead to unspecific binding to other proteins; such a design principle will be key for the development of second-generation compounds with increased Aβ-binding selectivity.

**Fig. 3:**
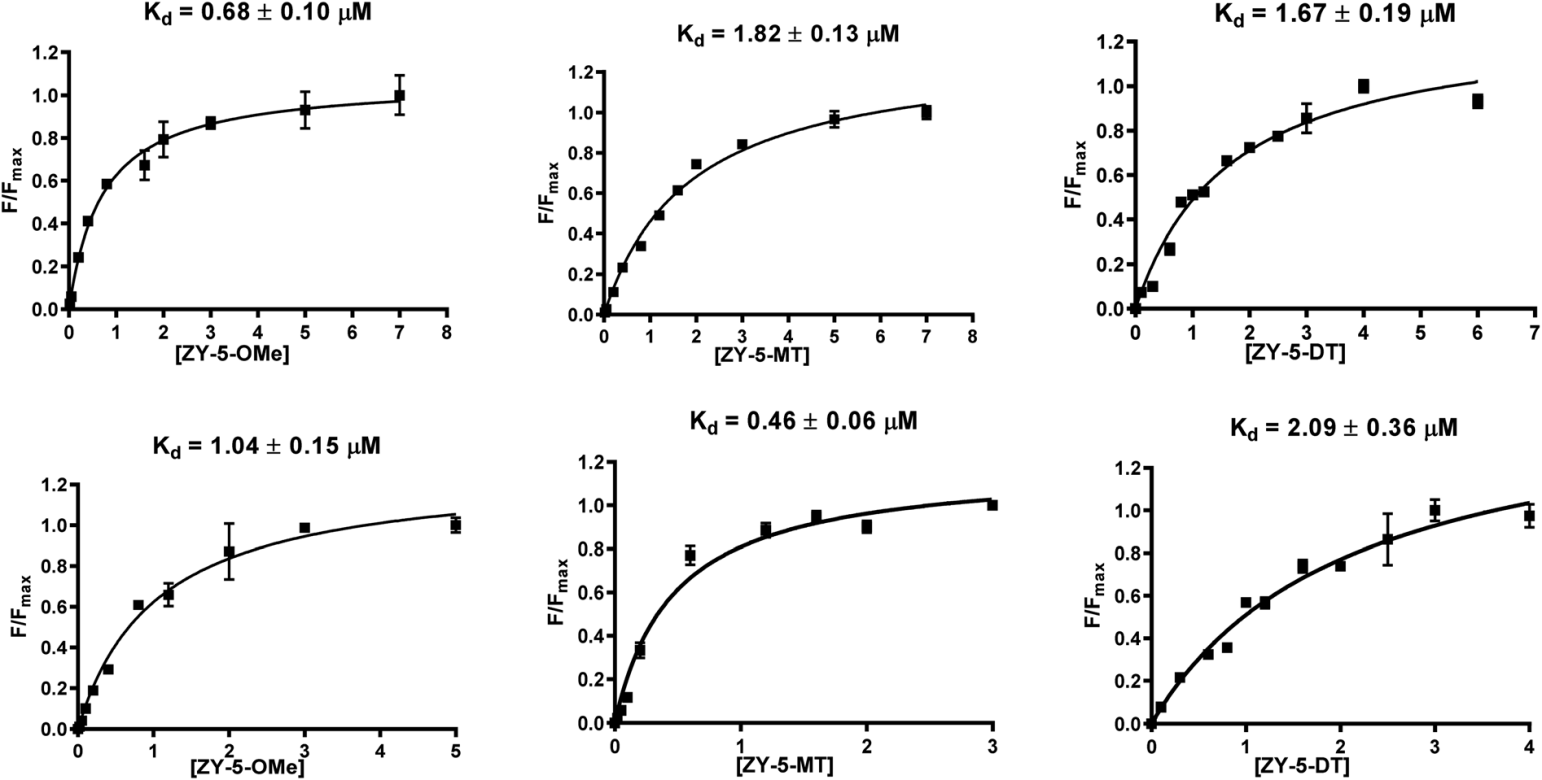
Binding constant measurements of ZY-5-OMe (left), ZY-5-MT (middle), ZY-5-DT (right) with Aβ_42_ fibrils (top), and oligomers (bottom). The measurements were performed in 10 mM phosphate buffered saline (PBS), pH 7.4. The *K*_d_ curves were fitted in GraphPad Prism with one site-specific binding model. Equation: *Y* = *B*_max_ × *X*/(*K*_d_ + *X*). [Aβ_42_ fibrils] = [Aβ_42_ oligomers] = 5 μM.

Inspired by the observed fluorescence turn-on effect, we then measured the binding constants (*K*_d_ values) toward Aβ_42_ fibrils and oligomers using fluorescence saturation assays. One site-specific binding model was used for all the compounds to rank the binding affinities, while there is the potential that some compounds might have multiple binding sites. Representative *K*_d_ curves and *K*_d_ values obtained for the ZY-5 series are shown in Fig. 3 (for other compounds, see Fig. S5 and S6†). *K*_d_ values for all twelve compounds provide a direct comparison of their interaction with fibrils and oligomers (Table 1). Even though the *K*_d_ values are tentative, by performing the same type of fluorescence titration assays, the affinity ranking orders can be used to select oligomer-specific compounds for further studies. Thioflavin T (ThT) was shown to have a binding ratio of 30 to 40 (*i.e.* 1 ligand per 35 Aβ_42_ monomers), while the binding ratios for our amphiphilic compounds are mostly between 2 and 10 (*i.e.* 1 ligand per 5 Aβ_42_ monomers, Table S2†), which is similar to some halogenated benzothiazole or benzofuran derivatives, such as TZDM, TZPI, and BF1.^29^ The different binding ratios suggest that these compounds would bind to a different but a higher density binding site *vs.* ThT. For diagnostic purposes, targeting the higher density binding site may help to generate enhanced *in vivo* signals from imaging agents bound to Aβ_42_ aggregates.^29^ Considering the difference between the soluble Aβ_42_ oligomers and the insoluble Aβ_42_ fibrils, we consider the extra hydrophilic interactions between the amphiphilic compounds and Aβ aggregates, such as hydrogen bonding and cation–π interactions, would contribute to targeting the higher density binding site, which would be even more beneficial for oligomer binding and *in vivo* targeting.^29^ It is worth noting that some compounds have high binding affinity to Aβ_42_ oligomers evidenced by the low *K*_d_ values (*e.g.*, ZY-17-OMe: *K*_d_ = 0.12 μM, ZY-12-OMe: *K*_d_ = 0.32 μM). These values are comparable to the *K*_d_ values of other reported oligomer-selective probes: CRANAD-102 (7.5 ± 10 nM),^30^ BD-Oligo (*K*_d_ = 0.48 μM),^31^ and F-SLOH (*K*_d_ = 0.66 μM).^32^ More interestingly, six of the compounds (ZY-5-MT, ZY-12-OMe, ZY-12-DT, ZY-15-OMe, ZY-17-OMe, and ZY-17-DT) showed relatively lower *K*_d_ values for Aβ oligomers than Aβ fibrils, indicating their higher binding affinity to oligomers over fibrils. Except for ZY-5-OMe, the other compounds bearing methoxy groups ZY-12-OMe, ZY-15-OMe, and ZY-17-OMe showed higher affinity toward Aβ oligomer suggesting that the methoxy group might increase the compounds' interactions with Aβ oligomers. Moreover, the ZY-5 and ZY-12 series bind to Aβ species differently despite their similar chemical structures. More specifically, ZY-5-MT showed a high affinity to both fibrils and oligomers, with an increased selectivity to Aβ oligomers (of 3.9 times higher than that for Aβ fibrils). In contrast, ZY-12-MT exhibited low affinity to both fibrils and oligomers. Taken together, we consider the different structures developed along with the *K*_d_ values will be beneficial for developing more oligomer-specific compounds.

**Table 1:** Summary of *K*_d_ values of amphiphilic compounds binding to Aβ_42_ fibrils and oligomers

Compounds	Fibrils (μM)	Oligomers (μM)
ZY-5-OMe	0.68	1.04
ZY-5-MT	1.82	0.46
ZY-5-DT	1.67	2.09
ZY-12-OMe	0.39	0.32
ZY-12-MT	4.19	6.27
ZY-12-DT	1.38	0.67
ZY-15-OMe	1.24	0.47
ZY-15-MT	0.53	2.03
ZY-15-DT	1.27	4.55
ZY-17-OMe	0.18	0.12
ZY-17-MT	0.94	2.08
ZY-17-DT	2.25	1.03

### Fluorescence imaging of 5xFAD mouse brain sections

**Fig. 4:**
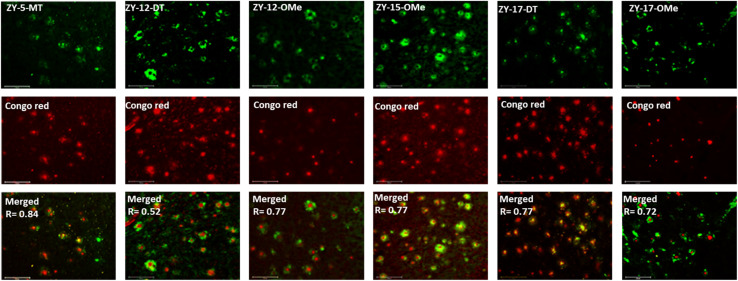
Fluorescence microscopy images of 5xFAD mice brain sections co-incubated with amphiphilic compounds (top), Congo red (middle) and merged images (bottom, along with the Parson's correlation coefficients *R*). Concentrations: [amphiphilic compounds] = 5 μM, [Congo red] = 2.5 μM; scale bar: 125 μm.

**Fig. 5:**
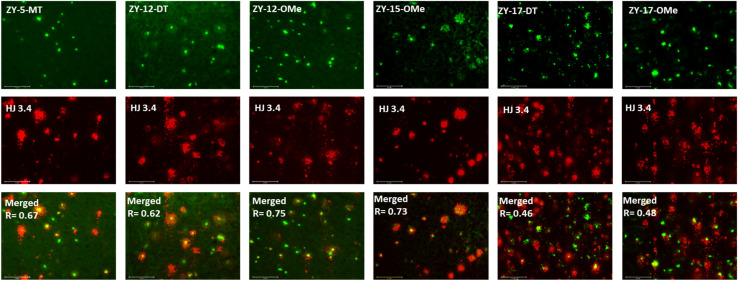
Fluorescence microscopy images of 5xFAD mice brain sections co-incubated with amphiphilic compounds (top), HJ 3.4 (middle) and merged images (bottom, along with the Pearson's correlation coefficients *R*). Concentrations: [amphiphilic compounds] = 5 μM, [CF594-HJ 3.4]: 1 μg mL^−1^; scale bar: 125 μm.

In order to confirm that the compounds can also bind to native Aβ species, brain sections collected from 9-month-old 5xFAD transgenic mice have been employed in the fluorescence imaging studies. 5xFAD transgenic mice were shown to develop AD pathologies at a young age, and aggregated Aβ species were commonly observed in brain sections from 5xFAD transgenic mice.^33^ The brain sections were first incubated with our compounds, followed by Congo Red (CR), which is a well-established fluorescent probe that can bind to Aβ plaques.^34^ The treated brain sections were then imaged using fluorescence microscope. The six compounds with high binding affinities for Aβ_42_ oligomers based on *K*_d_ measurements showed well-defined fluorescence staining signals and good colocalization with Congo Red (CR), as indicated by the Pearson's correlation coefficients (Fig. 4 and S7†). Interestingly, these compounds tend to bind preferentially to the periphery of the amyloid plagues, while CR binds to the dense core region of the plaques. Immunostaining with the CF594-conjugated HJ 3.4 antibody (CF594-HJ3.4) was also performed to confirm that these six compounds can bind to Aβ species specifically in the brain sections (Fig. 5). Moreover, the log *D* values of the compounds were also measured by octanol-PBS partition assays.^35^ These twelve compounds exhibit similar log *D* values ranging from 0.8 to 1.2 (Table S3†), indicating their ability to cross the blood–brain barrier (BBB) for potential *in vivo* applications.^36,37^

### Molecular docking studies

**Fig. 6:**
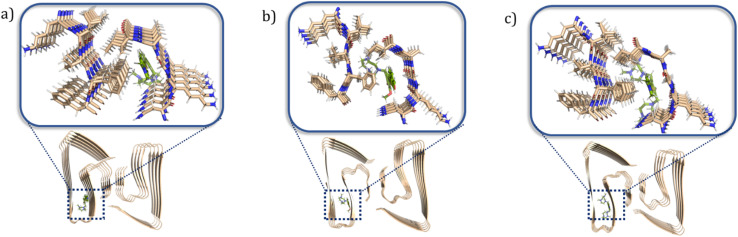
Calculated binding modes of amphiphilic compounds to Aβ_42_ fibrillar structure (5OQV). (a) ZY-5-MT (b) ZY-15-OMe (c) ZY-12-DT.

To better understand at a molecular level why some compounds showed a higher binding affinity to Aβ_42_ oligomers than fibrils, a series of docking studies were performed using the Schrödinger program Glide.^38^ Schröder and coworkers reported a Aβ_42_ fibrils structure (PDB ID: 5OQV) obtained by cryo-electron microscopy in 2017, and the structure had been widely used as a molecular docking model to study the binding of small molecules to Aβ_42_ fibrils.^39–41^ The structures of Aβ_42_ oligomers are less defined due to their heterogeneous and aggregation-prone nature. Recently, Aβ_42_ tetramers (PDB ID: 6RHY) in membrane-mimicking conditions were prepared and reported based on NMR spectroscopy and mass spectrometry by Carulla and coworkers.^42^ According to their molecular dynamic studies, the tetramer dimerizes and forms pore-like structures within the membranes. This structure could be a critical model for understanding how the compounds disrupt the interactions between the toxic oligomers and cell membranes. In our docking studies, compounds ZY-5-MT, ZY-12-DT, ZY-15-OMe were investigated due to their higher binding affinity towards Aβ oligomers *vs.* Aβ fibrils. Even though ZY-17-DT and ZY-17-OMe also showed higher affinities towards Aβ oligomers, they were not chosen due to their decreased selectivity towards HSA. The docking scores and glide e-model energies were summarized in Tables S4 and S5.† When compounds ZY-5-MT, ZY-15-OMe, and ZY-12-DT were docked onto the structure of Aβ_42_ fibrils' structure, neither hydrogen bond interactions nor π–π interactions were observed. Instead, these compounds bind to the Aβ_42_ fibrils by inserting into the pocket formed by the residues KLVFF and residues NKGAI (Fig. 6a–c), similar to ThT – a well-known compound used for Aβ fibril binding.^43,44^ Even though the binding ratio determination indicates that our compounds and ThT would bind to different sites, it was demonstrated that the two binding sites are spatially close to each other *via* fluorescent resonance energy transfer (FRET) measurements.^29^ Therefore, we consider that the stilbene-like fragments of the compounds bind similar to ThT, and mainly interacting with the β-sheet structures. In addition, the TACN azamacrocycle would reach out and interact with the polar side chains, and the combination of both hydrophobic and hydrophilic fragments should lead to slightly different binding pockets and different binding ratios.

**Fig. 7:**
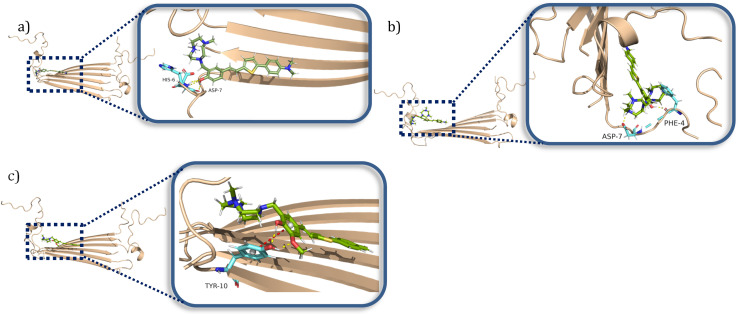
Calculated binding modes of amphiphilic compounds to Aβ_42_ tetramer structure (6RHY). (a) ZY-5-MT (b) ZY-12-DT (c) ZY-15-OMe.

When considering the docking results with the Aβ_42_ tetramers, the compounds exhibit more interactions with these oligomeric structures. ZY-5-MT interacts with His6 and Asp7 *via* hydrogen bonds and salt bridges (Fig. 7a). On the other hand, ZY-12-DT interacts with Phe4 and Asp7 instead of His6 and Asp7 (Fig. 7b). ZY-15-OMe binds to another amino acid – Tyr10 – through hydrogen bonds and π–π interactions (Fig. 7c). Since the compound ZY-5-MT (with the highest selectivity for Aβ oligomers) shows interactions with amino acids His6 and Asp7, we posited that targeting these two residues would increase the binding affinity and selectivity to Aβ oligomers.

### Modulation of Cu^2+^–Aβ_42_ neurotoxicity

**Fig. 8:**
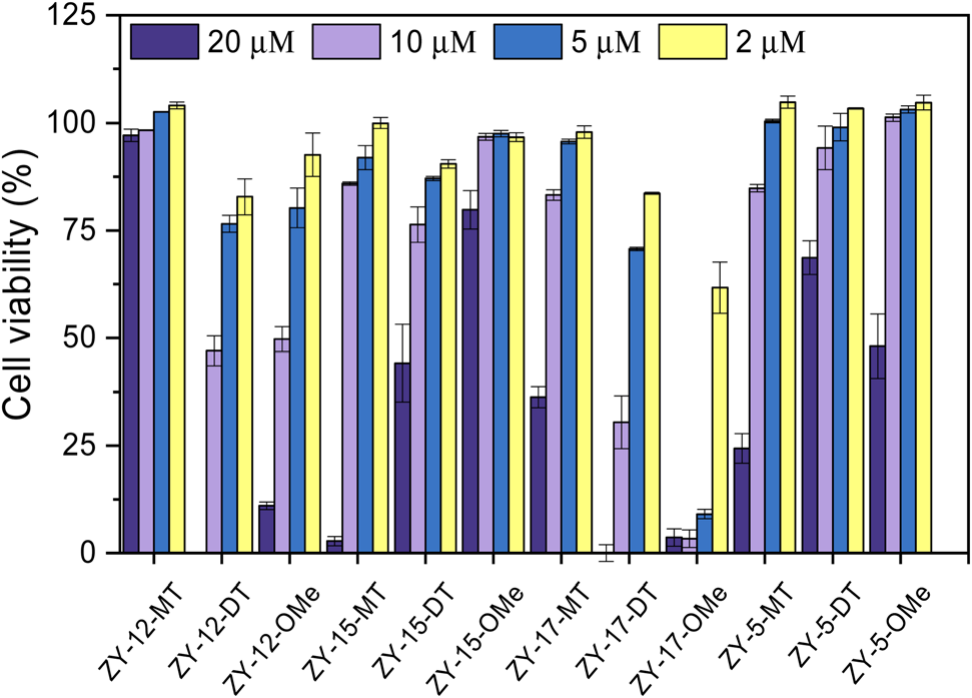
Toxicity of the amphiphilic compounds at different concentrations in mouse neuroblastoma Neuro2A (N2a) cells.

After confirming these amphiphilic compounds can bind to Aβ species both *in vitro* and *ex vivo*, we next investigated whether these compounds could attenuate the toxicity of the Cu–Aβ species, as the Cu^2+^ ions were reported to promote the formation of neurotoxic soluble Aβ_42_ oligomers.^45,46^ Firstly, the Alamar Blue cell viability assay was used to measure the cytotoxicity of the compounds at different concentrations ranging from 20 μM to 2 μM in mouse neuroblastoma N2a cells (Fig. 8). Some compounds (ZY-12-MT, ZY-15-MT, ZY-15-OMe, ZY-17-MT, ZY-5-MT, ZY-5-DT, and ZY-5-OMe) exhibited no significant cytotoxicity (indicated by >80% cell viability) up to 10 μM. Hence, these compounds are good candidates for the Cu^2+^–Aβ_42_-induced cytotoxicity studies (see below). For ZY-12-OMe, the compound was quite toxic even at 10 μM (cell viability less than 50%), yet at 5 μM it exhibited less cytotoxicity (more than 75% cell viability). Considering its high binding affinity to both Aβ fibrils and oligomers, we also further tested its ability to alleviate Cu^2+^–Aβ_42_-induced toxicity. Unfortunately, some of the compounds showed a high binding affinity to oligomers, such as ZY-12-OMe, ZY-12-DT, ZY-17-DT, and ZY-17-OMe, exhibited higher cytotoxicity than others. Additionally, ZY-17-OMe, which has the highest binding affinity to oligomers, showed the highest toxicity. The underlying mechanism is unclear, but we propose that these compounds might perform similarly to the toxic oligomers that bind to some cell membrane receptors or insert into the membrane lipids to form porous channels.

**Fig. 9:**
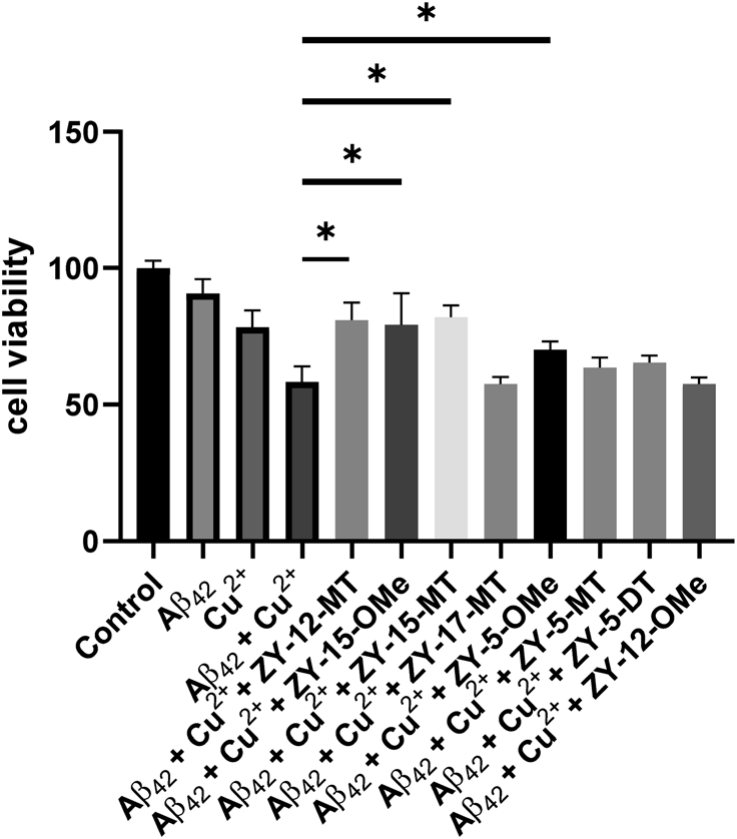
Cell viability results upon incubation of Neuro2A cells with monomeric Aβ_42_ in the presence or absence of metal ions and amphiphilic compounds. Concentrations: [Aβ_42_] = 40 μM, [Cu^2+^] = 40 μM, [ZY-12-MT] = [ZY-15-OMe] = [ZY-15-MT] = [ZY-17-MT] = [ZY-5-OMe] = [ZY-5-MT] = [ZY-5-DT] = 10 μM, [ZY-12-OMe] = 5 μM. The error bars represent the standard deviation from five independent experiments, and the statistical analysis was evaluated according to one-way ANOVA (*p < 0.05).

Since our compounds exhibit high binding affinity to both Aβ fibrils and oligomers and contain TACN group(s) that can potentially bind to Cu and disrupt the Cu–Aβ_42_ interaction,^47^ it is essential to study their roles in alleviating Cu^2+^–Aβ_42_-induced toxicity. As mentioned above, compounds that are not cytotoxic at 10 μM, including ZY-12-MT, ZY-15-MT, ZY-15-OMe, ZY-17-MT, ZY-5-MT, ZY-5-DT, ZY-5-OMe, and ZY-12-OMe, were chosen for this study. Firstly, during the control studies, we observed that monomeric Aβ_42_ led to negligible neurotoxicity. Nevertheless, in the presence of both Cu^2+^ and monomeric Aβ_42_, there was a significant cell death, which is likely due to the Cu^2+^ associated neurotoxic Aβ_42_ oligomers formation.^46^ We observed compounds ZY-12-MT, ZY-15-OMe, ZY-15-MT, and ZY-5-OMe could significantly increase cell viability, while the other ones could not reduce the neurotoxicity of the Cu^2+^–Aβ_42_ species (Fig. 9). Furthermore, the Cu-chelating fragments Me_2_HTACN or Me_3_TACN were not able to alleviate the neurotoxicity of the Cu^2+^–Aβ_42_ species (Fig. S8†), confirming the neuroprotective effect of the developed amphiphilic compounds is not solely due to the copper chelation ability and that the hydrophobic fragment also plays an essential role. Interestingly, when compared to ZY-5-MT, even though ZY-15-OMe is less selective toward Aβ oligomers, it can alleviate Cu^2+^–Aβ_42_-induced toxicity, likely due to its interaction with Tyr10 *via* hydrogen bond and π–π interactions. According to the docking results, ZY-12-MT, ZY-15-OMe, ZY-15-MT, and ZY-5-OMe interact with Aβ_42_ tetramers mainly through residues His6, Asp7, Tyr10 (*via* hydrogen bonds), and Tyr10 (*via* π–π interactions). Some reports have also shown that His6, Asp7, and Tyr10 are potentially involved in the Cu–Aβ interactions, which explains why the compounds are able to attenuate the Cu–Aβ toxicity.^48^

### Modulating the Aβ–cell membrane interactions

**Fig. 10:**
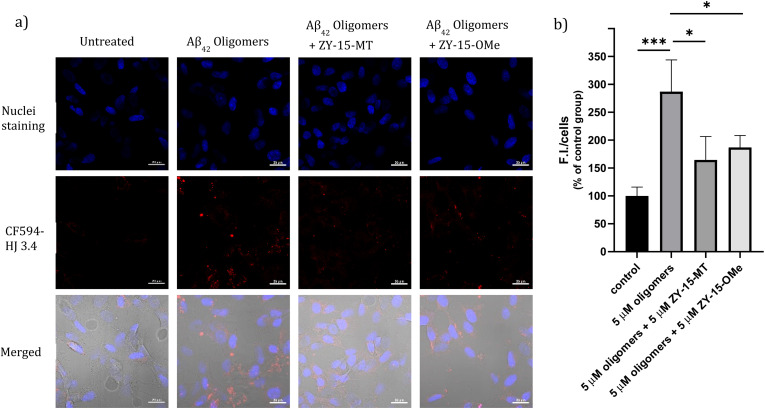
(a) SH-SY5Y cells were treated with 5 μM Aβ oligomers in the presence or absence of 5 μM ZY-15-MT or 5 μM ZY-15-OMe for 24 h before imaging. Red and blue fluorescence indicate the Aβ oligomers and nuclei, respectively. Scar bar, 20 μm. (b) Three independent experiments were subjected for the statistical analysis and analyzed by one-way ANOVA (*P < 0.05, ***P < 0.001).

**Fig. 11:**
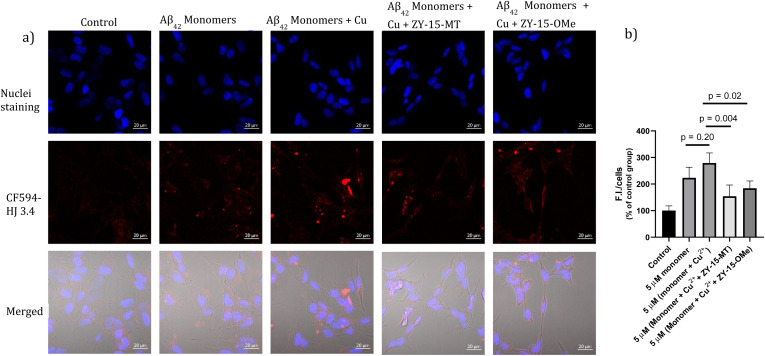
(a) SH-SY5Y cells were treated with 5 μM Aβ monomers in the presence or absence of 5 μM Cu^2+^, 5 μM (Cu^2+^ + ZY-15-MT) or 5 μM (Cu^2+^ + ZY-15-OMe) for 48 h before imaging. Red and blue fluorescence indicate the Aβ species and nuclei, respectively. Scar bar, 20 μm. (b) Three independent experiments were subjected for the statistical analysis and analyzed by one-way ANOVA (*P < 0.05, **P < 0.01).

Encouraged by the ability of the developed amphiphilic compounds to attenuate the neurotoxicity of Cu–Aβ species, we sought out to study the possible molecular mechanisms for this beneficial effect. The Aβ oligomers were reported to interact with cell membranes in various ways, such as binding to receptors on cell membranes,^49,50^ inserting into membranes,^51^ or even showing cellular uptake *via* endocytosis.^52^ The abnormal interactions between Aβ oligomers and neuron cells, which could disrupt the neuronal ion homeostasis and neuron cell membrane integrity, might be why Aβ oligomers are highly neurotoxic.^53–55^ Moreover, molecules that can disrupt interactions between oligomers and cell membranes are promising candidates for drug development.^56,57^ Thus, we proceeded to probe the interactions of the Aβ_42_ oligomers with SH-SY5Y cellular membranes, in the absence and presence of the compounds, *via* confocal microscopy. While all four compounds, ZY-12-MT, ZY-15-OMe, ZY-15-MT, and ZY-5-OMe, are not toxic towards SH-SY5Y cells (Fig. S9a†), ZY-15-MT and ZY-15-OMe were chosen for the cell imaging experiments, since they can rescue cell viability to a higher extent, and also exhibit a higher affinity for Aβ_42_ oligomers. Moreover, a lower concentration of Aβ_42_ (5 μM) or Aβ_42_–Cu (5 μM) were chosen to avoid significant cell death (Fig. S9b†). Before investigating the neuroprotective effect of our compounds, we have also confirmed that Aβ aggregates interact tightly with the cell membranes, as shown *via* co-staining with a membrane specific dye (Fig. S10†). Subsequently, SH-SY5Y cells were treated with Aβ oligomers or a combination of Aβ oligomers and compounds for 24 h, followed by the immunofluorescence staining with the CF594-labeled anti-Aβ antibody HJ 3.4 (Fig. 10) and nuclei staining, shown in the red and blue channels, respectively. Compared to the untreated group, the Aβ oligomers were found mainly bound to cell membranes. Moreover, in the presence of both ZY-15-MT and ZY-15-OMe, there were fewer numbers of Aβ oligomers bound to the cell membranes, as shown in the red channel (Fig. 10a). Interesting, ZY-15-MT is able to decrease the numbers of the Aβ oligomers binding to the cell membranes to a larger extent (about 50%, Fig. 10b), even though ZY-15-MT shows a lower affinity to oligomers than ZY-15-OMe. To understand this unusual behavior, we posited that the Aβ oligomers would start the fibrilization process when incubated in the cell media. ZY-15-MT, which exhibits a higher affinity to Aβ fibrils than ZY-15-OMe, might be able to bind to the more aggregated Aβ species and can also decrease their interactions with cell membranes. Therefore, we proceeded to probe if our compounds could prevent the binding of Aβ fibrils to cell membranes. When SH-SY5Y cells were treated with Aβ fibrils or a combination of fibrils and compounds for 24 h, both ZY-15-MT and ZY-15-OMe were not able to significantly decrease the numbers of the Aβ fibrils binding to cell membranes, while ZY-15-MT seems to decrease the interactions between Aβ fibrils and SH-SY5Y more than ZY-15-OMe (Fig. S11†). These results further suggest that both ZY-15-MT and ZY-15-OMe are more potent to alleviate the neurotoxicity of the Aβ oligomeric species. Given these encouraging results, we moved to study if the amphiphilic compounds can further control the interactions between Cu–Aβ species and cell membranes. Firstly, when SH-SY5Y cells were treated with Aβ monomers for 48 h, the aggregated Aβ species interacting with cell membranes were observed (Fig. 11a). Moreover, in the presence of Cu^2+^, even though there were only slightly increased interactions with membranes, larger aggregated Aβ species were observed compared to the group only with Aβ monomers treated (Fig. 11a and b). However, these aggregates are still smaller than the insoluble fibrils (Fig. S11†), indicating they are still soluble oligomeric Aβ species. Excitingly, in the presence of either compound, the Cu–Aβ species showed significantly decreased interactions with cell membranes (Fig. 11a and b), which might explain why the compounds can attenuate the toxicity of the Cu–Aβ aggregates. Taken together, we believe these findings suggest the developed amphiphilic compounds are neuroprotective by controlling the interactions between the Aβ oligomers and cell membranes, which we consider is a novel approach for the development of AD therapeutics.

## Conclusions

In conclusion, we have designed and synthesized twelve amphiphilic compounds and studied their binding affinity toward Aβ_42_ species *in vitro* and *ex vivo*. Six of the compounds showed higher binding affinity towards Aβ_42_ oligomers, with compound ZY-5-MT showing the highest selectivity toward Aβ_42_ oligomers over Aβ_42_ fibrils. Interestingly, the less sterically hindered and less hydrophilic compounds are more selective towards Aβ_42_ oligomers in the ZY-5 and ZY-15 series, as evidenced by the DT compounds showing lower binding affinity towards both Aβ_42_ fibrils and Aβ_42_ oligomers compared to their ZY-MT or ZY-OMe analogs. However, when the stilbene backbone was changed, by replacing the thiophene with phenyl or switching the double bond position, both DT compounds were more selective towards Aβ_42_ oligomers in the ZY-12 and ZY-17 series. These structural studies indicate the hindrance of the molecules may not be the major factor, and we believe that the combination of hydrophobic fragments and hydrophilic groups is critical for the development of Aβ_42_ oligomer-selective compounds. More importantly, according to molecular docking studies, we found that targeting amino acids His6 and Asp7 might increase the binding affinity and selectivity toward Aβ_42_ oligomers. Based on cellular studies, we found that compounds with a higher binding affinity toward Aβ_42_ oligomers exhibit higher neurotoxicity, and most of them interact mainly with the amino acids His6 and Asp7. The only exception is compound ZY-15-OMe, which maintains its selectivity to Aβ_42_ oligomers *via* hydrogen bonds and π–π interactions with Tyr10, and, while exhibiting lower inherent cell toxicity, it was able to alleviate the Cu–Aβ_42_ neurotoxicity. Finally, confocal microscopy imaging studies show that both ZY-15-MT and ZY-15-OMe were able to decrease the interactions between Aβ_42_ oligomers and SH-SY5Y cell membranes, demonstrating their ability to target Aβ_42_ oligomers. These studies strongly suggest that developing such amphiphilic compounds could be an effective strategy to differentiate between Aβ_42_ oligomers and Aβ_42_ fibrils. We believe these encouraging results will help design Aβ_42_ oligomer-selective lead compounds to be used for AD therapeutic agent development.

## Data availability

General methods, synthetic details, fluorescence imaging studies, molecular docking studies, and any additional experimental details are available in the ESI.† Any additional data that support the findings of this study are available from the corresponding author upon request.

## Author contributions

L. M. M. directed the overall project. Z. Y. and L. M. M. conceived and designed the experiments. Z. Y. performed the chemical synthesis and *in vitro* characterization. Z. Y. and W. G. performed the cell imaging studies and analyzed data. Z. Y., S. P. and H.-J. C. performed the brain section imaging staining studies. Z. Y. and L. S. performed molecular docking studies. Z. Y. and L. M. M. wrote the paper with input from all authors.

## Conflicts of interest

The authors declare no competing financial interest.

## Supplementary Material

SC-013-D2SC02654F-s001
