# Supplementary material for: Amphiphilic stilbene derivatives attenuate the neurotoxicity of soluble Aβ42 oligomers by controlling their interactions with cell membranes
Source: Chem Sci. 2022 Oct 19;13(43):12818–30. doi: 10.1039/d2sc02654f (PMC9645390; doi:10.1039/d2sc02654f)
Supplement: SC-013-D2SC02654F-s001 [file SC-013-D2SC02654F-s001.pdf]

Electronic Supplementary Material (ESI) for Chemical Science.
This journal is © The Royal Society of Chemistry 2022

Supplementary Information

for

Amphiphilic Stilbene Derivatives Attenuate the Neurotoxicity of Soluble $A\beta_{42}$ Oligomers by Controlling Their Interactions with Cell Membranes

Zhengxin Yu,[a] Weijie Guo,[b] Shrey Patel,[a] Hong-Jun Cho,[a] Liang Sun,[a] and Liviu M. Mirica[a,c *]

[a] Department of Chemistry, Beckman Institute for Advanced Science and Technology, The Neuroscience Program, University of Illinois at Urbana-Champaign, 600 S. Mathews Avenue, Urbana, Illinois 61801, United States

[b] Department of Biochemistry, University of Illinois at Urbana-Champaign, 600 S. Mathews Avenue, Urbana, Illinois 61801, United States

[c] Hope Center for Neurological Disorders, Washington University School of Medicine, St. Louis, MO 63110, United States

* e-mail: mirica@illinois.edu

Table of Contents

1. General methods S2
2. Experimental details S3
3. Synthetic details S6
4. Optical properties S15
5. Fluorescence turn-on studies S17
6. K_d measurements and binding ratio S19
7. 5xFAD mouse brain section fluorescence imaging S22
8. Log D measurements S23
9. Docking scores and Glide e-model energies S24
10. The effect of copper chelators on Cu^{2+}-$A\beta_{42}$ neurotoxicity S25
11. Cell toxicity studies in SH-SY5Y cells S26
12. Cell membrane staining S27
13. $A\beta_{42}$ Fibrils and cell membrane interactions S28
14. NMR spectra S29
15. References S41

1. General methods

All reagents and solvents were purchased from commercial sources and used without further purification unless otherwise stated. 1,4-dimethyl-1,4,7-triazacyclononane (Me_2HTACN) or 1,4,7-trimethyl-1,4,7-triazacyclononane (Me_3TACN) were synthesized according to reported procedures.[1] HRMS data were obtained on a high-resolution electrospray ionization mass spectrometry (HR-ESI-MS, Thermo Scientific™ LTQ Orbitrap XL™ Hybrid Ion Trap-Orbitrap) (Thermo Scientific, USA). UV−visible spectra were recorded on a Varian Cary 50 Bio spectrophotometer, and fluorescence emission spectra were measured by a SpectraMax M2e plate reader (Molecular Devices, USA). 5xFAD transgenic mice overexpressing mutant human APP (695) with the Swedish (K670N, M671L), Florida (I716V), and London (V717I) were purchased from Jackson Laboratories (USA). Monoclonal anti-Aβ-antibody HJ 3.4 (anti-$A\beta_{1-13}$)[2] was obtained from Dr. Holtzman lab in the department of neurology at Washington University. The antibody was directly labeled with CF™ 594 dye using Mix-n-Stain™ CF™ 594 Antibody Labeling Kit purchased from Millipore Sigma (USA). Fluorescence images for brain sections were visualized using an Invitrogen EVOS FL Auto 2 Imaging System (Thermo Fisher, USA). Colocalization analysis and determination of the Pearson′s correlation coefficient was performed with the imaging software Fiji. Mouse neuroblastoma N2a cell line and human neuroblastoma SH-SY5Y cell line were purchased from Millipore Sigma (USA). Confocal cell imaging studies were performed on a Zeiss LSM 880 confocal microscope, cell images were analyzed with Fiji and Zeiss Zen lite software. Compound purification was performed on a Teledyne Isco Combiflash Rf+. ^{1}H and ^{13}C NMR spectra were recorded on Varian 400, Varian 500, or Carver B500 spectrometers. Spectra were analyzed and visualized with MestReNova (15.0). All other data analysis was performed using GraphPad Prism (8.0) or Origin 2020.

2. Experimental details

Preparation of the Aβ_{42} Fibrils and Aβ_{42} Oligomers

Aβ_{42} fibrils: 1 mg of commercial Aβ_{42} monomer powder (from GL biochem) was dissolved in 1 mL 1,1,1,3,3,3-hexafluoro-2-propanol (HFIP) at room temperature and incubated for 1h. The resulting solution was divided equally into two Eppendorf tubes. The solution in each Eppendorf tube containing 0.5 mg Aβ_{42} monomer was then evaporated overnight and dried by vacuum centrifuge to generate monomeric films. 100 µM Aβ_{42} fibrils solution was prepared by dissolving the 0.5 mg monomeric films in 1.1 mL PBS buffer (with final DMSO concentration less than 1%) and stiring at 37 °C for 3 days.

Aβ_{42} oligomers: The 0.5 mg monomeric films were dissolved in 1.1 mL PBS buffer (with final DMSO concentration less than 1%) and incubated at 4 °C overnight. The resulting solution was then centrifuged at 3000 rpm for 15 mins to remove the insoluble aggregates. The clear supernatant was then used for the following experiments. Aβ_{42} oligomers concentration was checked by measuring the absorbance of the supernatant at 280 nm with the molar extinction coefficient of 1480 M^{-1} cm^{-1}.

Fluorescence Spectral Testing of Compounds with Aβ_{42} Fibrils and Aβ_{42} Oligomers

The formation of Aβ_{42} fibrils and oligomers was confirmed by the ThT fluorescence turn-on assay. The fluorescence spectra of ThT in PBS (100 µL PBS, 10.0 µM) were recorded as the baseline. After that, either Aβ_{42} fibrils or oligomers were added to ThT in PBS. The final volume of the solution mixture should be 100 µL, in which the final concentrations of ThT and Aβ_{42} species were10.0 µM and 25 µM, respectively.

For the interactions between Aβs and compounds, the fluorescence spectra of the compounds' solution (100 µL PBS, 5.0 µM) were recorded as the baseline without adding various Aβ_{42} species. After that, either Aβ_{42} fibrils or oligomers were added to the compounds' solutions in PBS. The final volume of the solution mixture should be 100 µL, in which the final concentrations of the compound and Aβ_{42} species were 5.0 µM and 25 µM, respectively.

In Vitro Saturation Binding Studies with Aβ_{42} Fibrils and Aβ_{42} Oligomers

To a solution of increasing concentrations of compounds, a fixed concentration of the generated aggregated Aβ_{42} fibrils or Aβ_{42} oligomers solutions (5 to 15 µM) was added to yield a total volume of 100 µL. Nonspecific binding was determined without compounds. The mixture was incubated for 15 min at room temperature. All fluorescence data were obtained on a SpectraMax M2e plate reader (Molecular Devices). The fluorescent intensity was measured at the corresponding emission wavelength of each compound, and the K_d binding curves were generated by GraphPad Prism 8.0 with one site-specific binding model. Equation: $Y = B_{max}*X/ (K_d +X)$. For the binding ratio analysis, the concentration of bound compounds were calculated from the cross point between the plateau and the initial slope. The binding ratio = [bound compounds]/[Aβ].

Cytotoxicity Studies

Alamar Blue assay was chosen for the cytotoxicity studies.[3] Mouse neuroblastoma Neuro2A (N2A) cells were grown with DMEM/10% FBS in a petri dish at 37 °C in a humidified atmosphere with 5% CO_2. Then N2A cells were seeded in a 96-well plate (1.0×10^4/well). After 24 h incubation, cell media was changed to DMEM/N-2, and N2A cells were incubated for another 1 h. Then the cells were treated with different concentrations of compounds for 40 h, Alamar Blue solution (10 μL) was added, and the cells were incubated for another 90 min at 37 °C. The fluorescence intensity of each well was measured at 590 nm (excitation wavelength = 560 nm).

For the Cu^{2+}-Aβ-induced toxicity studies, cells were treated monomeric $A\beta_{42}$, Cu^{2+}, monomeric $A\beta_{42}$ + $Cu^{2+,}$ and monomeric $A\beta_{42}$ + Cu^{2+} + compounds, monomeric Aβ42 + Cu^{2+} + copper chelators (Me_2HTACN or Me_3TACN), respectively. After 40 h incubation, Alamar Blue solution (10 μL) was added, and the cells were incubated for another 90 min at 37 °C. The fluorescence intensity of each well was measured at 590 nm (excitation wavelength = 560 nm).

Histological Staining of 5×FAD Mice Brain Sections

5xFAD transgenic mice brain sections were blocked with bovine serum albumin (2% BSA in PBS, pH 7.4, 10 min). Then the mice brain sections were transferred to a PBS solution of the compound and incubated for 1 h. After which, the brain sections were transferred to a PBS solution of Congo Red or antibody HJ 3.4 (Professor David Holtzman, 1 μg/ml) and incubated for another 1 h. Then, the brain sections were treated with BSA again (5 min) followed by washing with PBS (3 × 2 min), DI water (3 × 2 min). Finally, the mice brain sections were mounted with non-fluorescent mounting media, and the images were obtained by using EVOS FL Auto 2. ImageJ Fiji program was used for colocalization analysis and determination of the Pearson's correlation coefficient. The primary antibodies were labeled with dye CF594 via Mix-n-Stain™ CF™ 594 Antibody Labeling Kit (Sigma Aldrich).

Log D measurements

The Log D value was measured following a published procedure using slight modifications.[4] In general, compounds in 0.5 mL octanol was subjected to partition with 0.5 mL octanol-saturated PBS. The whole mixture was stirred vigorously for 5 min, and centrifuged at 2,000 rpm for 5 min. The top octanol layer was separated for later fluorescence measurement. The remaining PBS layer was partitioned with 0.5 mL PBS-saturated octanol, and the second top octanol layer was then separated after vigorous stirring and centrifuging at 2,000 rpm for another 5 min. The two octanol layers' fluorescence spectra were recorded. The log D value was calculated by the fluorescence intensity ratio at the compounds' emission wavelength for the above two octanol extractions.

Molecular Docking

Molecular docking studies were performed with the Schrödinger Suite software. Protein structure $A\beta_{42}$ tetramers (PDB ID: 6RHY) and the $A\beta_{42}$ fibrils (PDB ID: 5OQV) were imported from the

RCSB database and optimized by minimal minimization with the OPLS3 force field using the Protein Preparation Wizard program. The amphiphilic molecules were prepared using Ligprep, and the pH was set as 7.0 ± 2.0 using Epik. The resulting different protonation states of the compounds were obtained and used for the docking studies. The grid size was set to include the whole optimized protein structure in each direction. The final molecular docking was performed using Glide. The calculated poses were ranked by both the docking score and Glide e-model energy, and the structures with the best docking scores and Glide e-model energies were rendered in PyMol.[5]

Cell Imaging Procedures

For oligomers and fibrils imaging:
Human neuroblastoma SH-SY5Y cells were grown with DMEM/10% FBS at 37 °C in a humidified atmosphere with 5% CO2, and an 8-well μ-slides (ibidi) chambered coverslip was seeded the SH-SY5Y cells. At about 80% confluency (24 h after seeding), the cells were treated with DMSO as control group, 5 μM oligomers/fibrils and 5 μM (oligomers/fibrils + compounds), respectively. After 24 h incubation, the media was removed from each well. The cells were then fixed with 200 μL 3.7% formaldehyde for 15 min and blocked with 200 μL 3% BSA for 10 min. 3% BSA was then removed from the cells and replaced with 100 μL CF594-HJ 3.4 (1 μg/mL in 3% BSA). The cells were incubated with CF594-HJ 3.4 for 2 h at room temperature and were washed with PBS (5 x 100 μL). Before imaging, the cells in each well were stained with 100 μL NucBlue reagent (Thermo Fisher Scientific) for 15 min and mounted with non-fluorescent mounting media. 5-10 images of each well were taken, and the images were processed and analyzed by Fiji and Zeiss Zen lite software. Three individual replicates were conducted and subjected to the statistical analysis.

For monomers + Cu^{2+} imaging:
SH-5Y5Y were seeded as mentioned above, and cells were treated with DMSO as control group, 5 μM monomers, 5 μM (monomers + $CuCl_2$) and 5 μM (monomers + $CuCl_2$ + compounds) respectively. After 48 h incubation, cells were treated and imaged the same way as mentioned above.

For cell membrane staining:
BioTracker 490 green cytoplasmic membrane dye (Millipore Sigma, USA) was used according to the manufacture procedures.

3. Synthetic details

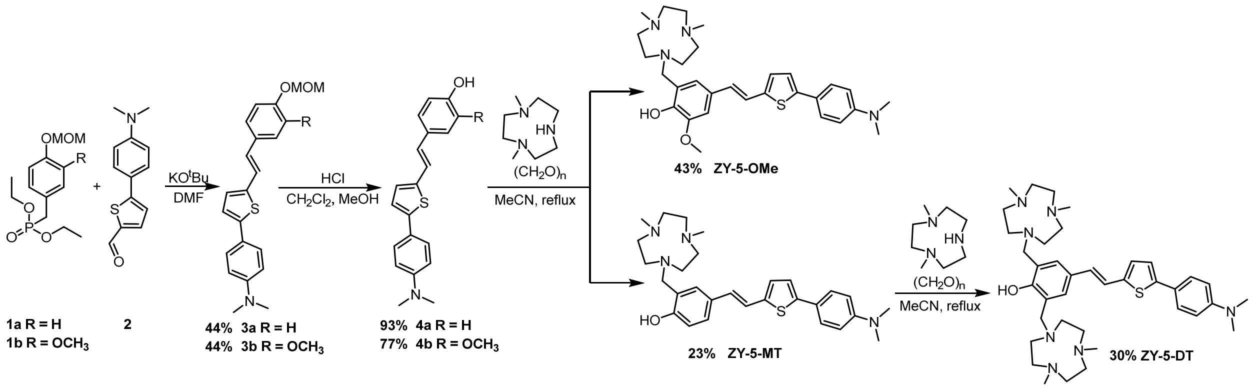

Scheme S1. Synthetic route for ZY-5-MT, ZY-5-DT and ZY-5-OMe.

*Compound **3a, 3b***: Compound **1a** (74 mg, 0.26 mmol, 1.5 eq) or **1b** (70 mg, 0.22 mmol, 2.0 eq) was added to a solution of compound **2** (40 mg, 0.17 mmol or 25 mg, 0.11 mmol, 1.0 eq) in 5 mL DMF. Then potassium tert-butoxide (39 mg, 0.35 mmol, 2.0 eq or 30 mg, 0.27 mmol, 2.5 eq) was added to the solution slowly, and the solution color turned dark red. The whole reaction mixture was stirred at room temperature overnight. Then the mixture was diluted with dichloromethane, and the organic solution was washed with brine 3 times. The organic layer was dried over $MgSO_4$ and concentrated. The residue was purified by flash column chromatography on silica gel (eluent: hexane and ethyl acetate, 3:1). Compound **3a** (28 mg) and **3b** (19 mg) were both obtained in 44% isolated yield.

3a: ^{1}H NMR (400 MHz, $CDCl_3$) δ (ppm): 7.57-7.46 (m, 2H), 7.43-7.33 (m, 2H), 7.12-6.99 (m, 4H), 6.95 (d, J = 3.7 Hz, 1H), 6.83 (d, J = 16.2 Hz, 1H), 6.73 (d, J = 8.4 Hz, 2H), 5.19 (s, 2H), 3.50 (s, 3H), 3.00 (s, 6H).
3b: ^{1}H NMR (500 MHz, $CDCl_3$): δ (ppm): 7.49 (d, J = 8.8 Hz, 2H), 7.12 (d, J = 8.3 Hz, 1H), 7.08 (d, J = 16.0 Hz, 1H), 7.05 (d, J = 3.6 Hz, 1H), 7.02 (d, J = 2.0 Hz, 1H), 6.99 (dd, J = 8.3, 2.0 Hz, 1H), 6.96 (d, J = 3.7 Hz, 1H), 6.84 (s, 1H), 6.73 (d, J = 8.4 Hz, 2H), 5.24 (s, 2H), 3.94 (s, 3H), 3.53 (s, 3H), 2.99 (s, 6H).

*Compound **4a** and **4b***: Concentrated hydrogen chloride (2 mL) was added to a solution of **3a** (28 mg, 0.077 mmol) or **3b** (19 mg, 0.097 mmol) in dichloromethane (5 mL) and methanol (5 mL). The resulting mixture was stirred at room temperature for 12 h. The mixture was diluted with dichloromethane, and the organic solution was washed with a saturated sodium bicarbonate solution. The organic layer was dried over $MgSO_4$ and concentrated. The residue was purified by flash column chromatography on silica gel (eluent: hexane and ethyl acetate, 3:1). Compound **4a** (23 mg, 0.072 mmol) and **4b** (13 mg, 0.036 mmol) were obtained in 93% and 77% isolated yield, respectively.

4a: ^{1}H NMR (500 MHz, $(CD_3)_2CO$) δ (ppm): 7.50 (d, J = 8.6 Hz, 2H), 7.40 (d, J = 9.0, 2H), 7.16 (d, J = 16.1, 1H), 7.13 (d, J = 3.6 Hz, 1H), 7.01 (d, J = 3.7 Hz, 1H), 6.85 (m, 3H), 6.77 (d, J = 8.8 Hz, 2H), 2.97 (d, J = 1.5 Hz, 6H). ^{13}C NMR (500 MHz, $(CD_3)_2CO$) δ (ppm): 206.15, 157.45, 150.27, 143.12, 140.56, 128.64, 127.55, 127.01, 126.21, 122.41, 121.11, 119.27, 115.67, 112.50,

39.57.
4b: ^{1}H NMR (400 MHz, $CDCl_3$) δ (ppm): 7.49 (d, J = 8.3 Hz, 2H), 7.11-6.93 (m, 4H), 6.95 (d, J = 3.8 Hz, 1H), 6.89 (d, J = 8.6 Hz, 1H), 6.81 (d, J = 16.0 Hz, 1H), 6.76-6.65 (d, J = 8.3 Hz, 2H), 3.95 (s, 3H), 2.99 (s, 6H).

*Compound **ZY-5-MT** and **ZY-5-OMe***: Paraformaldehyde (3 mg, 0.010 mmol) was added to a solution of 1,4-dimethyl-1,4,7-triazacyclononane (15 mg, 0.095 mmol) in MeCN (10 mL), and the mixture was refluxed for 30 min. Then a solution of compound **4a** (23 mg, 0.072 mmol) or **4b** (13 mg, 0.036 mmol) in MeCN (5 mL) was added to the reaction mixture, and the solution was further refluxed for another 16 h. The solvent was removed under vacuum, and the resulting residue was purified by C-18 reversed-phase column (eluent: MeCN/H_2O with 0.1% TFA, gradient wash from 10:90 to 30:70). The crude product collected from the reversed-phase column was then neutralized with saturated $NaHCO_3$ solution, extracted by dichloromethane (3x50 mL). The organic solvent was dried over $MgSO_4$ and concentrated to yield the final products **ZY-5** (8.0 mg) and **ZY-5-OMe** (8.3 mg) in 23% and 43% isolated yield, respectively.

ZY-5-MT: ^{1}H NMR (500 MHz, $CDCl_3$) δ (ppm): 7.53-7.49 (m, 2H), 7.31 (dd, J = 8.7, 2.2 Hz, 1H), 7.13 (d, J = 2.2 Hz, 1H), 7.07-7.00 (m, 2H), 6.95-6.90 (m, 2H), 6.81 (d, J = 16.0 Hz, 1H), 6.77-6.72 (m, 2H), 3.85 (s, 2H), 3.01 (s, 6H), 2.90 (dd, J = 6.7, 3.6 Hz, 4H), 2.78-2.69 (m, 7H), 2.49 (s, 6H). ^{13}C NMR (126 MHz, $CDCl_3$) δ (ppm): 157.93, 150.09, 143.34, 141.02, 128.28, 127.38, 127.30, 127.10, 126.71, 126.60, 123.58, 123.05, 121.30, 119.43, 117.06, 112.67, 60.33, 56.81, 52.77, 45.82, 40.62, 32.08, 29.85, 29.52, 22.84, 14.27.
HRMS: calculated exact mass = 491.2872 for $[M+H]^+$, found 491.2849.

ZY-5-OMe: ^{1}H NMR (400 MHz, CD_3CN) δ (ppm): 7.55 -7.45 (m, 2H), 7.22 (dd, J = 16.1, 0.7 Hz, 1H), 7.19 -7.07 (m, 2H), 7.01 (d, J = 3.8 Hz, 1H), 6.97 (d, J = 2.0 Hz, 1H), 6.90-6.68 (m, 3H), 3.91 (s, 3H), 3.89 (s, 2H), 2.97 (s, 6H), 2.81 (m, 12H), 2.49 (s, 6H).
HRMS: calculated exact mass = 521.2972 for $[M+H]^+$, found 521.2950.

*Compound **ZY-5-DT***: Paraformaldehyde (5 mg, 0.167 mmol) was added to a solution of 1,4-dimethyl-1,4,7-triazacyclononane (20 mg, 0.127 mmol) in MeCN (10 mL), and the mixture was refluxed for 30 min. Then a solution of compound **ZY-5-MT** (23 mg, 0.047 mmol) in MeCN (5 mL) was added to the reaction mixture, which was refluxed for another 24 h. The solvent was removed under vacuum, and the resulting residue was purified by C-18 reversed-phase column (eluent: MeCN/H_2O with 0.1% TFA, gradient wash from 10:90 to 30:70). The crude product collected from the reversed-phase column was then neutralized with saturated $NaHCO_3$ solution, extracted by dichloromethane (3x50 mL). The organic solvent was dried over $MgSO_4$ and concentrated to yield the final product **ZY-5-DT** (9.3 mg) in 30% isolated yield.

ZY-5-DT: ^{1}H NMR (500 MHz, $CDCl_3$) δ (ppm): 7.53-7.48 (m, 2H), 7.22 (s, 2H), 7.07 (m, 2H), 6.97 (d, J = 3.7 Hz, 1H), 6.80- 6.71 (m, 3H), 3.96 (s, 4H), 3.02 (m, 14H), 2.97 (m, 8H), 2.91-2.83 (m, 8H), 2.57 (s, 12H). ^{13}C NMR (126 MHz, $CDCl_3$) δ (ppm): 154.73, 149.03, 142.79, 139.19, 127.84, 127.54, 126.11, 125.58, 125.08, 123.46, 121.64, 120.19, 119.41, 111.47, 57.23, 53.58, 52.65, 51.01, 43.42, 39.43, 30.91, 28.68, 28.35, 21.68, 13.11.
HRMS: calculated exact mass = 660.4373 for $[M+H]^+$, found 660.4402.

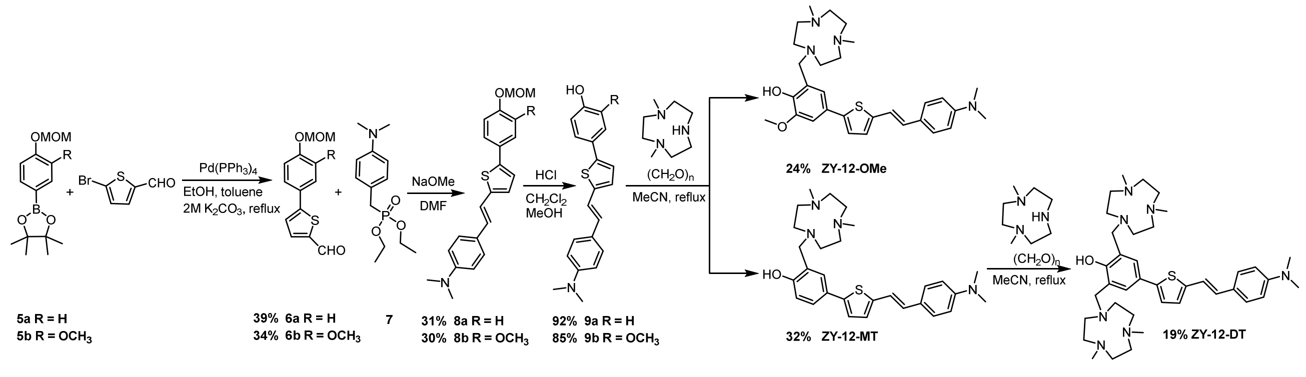

Scheme S2. Synthetic route for ZY-12-MT, ZY-12-DT and ZY-12-OMe.

*Compound **6a** and **6b***: (4-bromobenzaldehyde) (300 mg, 1.57 mmol, 1.3 eq) and **5a** (327mg, 1.24 mmol, 1 eq) or **5b** (365 mg, 1.24 mmol, 1.0 eq) were dissolved in a mixture of 10 ml ethanol and 10 ml toluene. 2 ml of an aqueous K_2CO_3 (2M) solution was added to the reaction mixture followed by the addition of $Pd(PPh_3)_4$ (70 mg, 0.062 mmol, 0.05 eq). Then the mixture was stirred and refluxed for 6 h under a nitrogen atmosphere. The solvent was removed under vacuum, and the residue was washed with brine and extracted with dichloromethane. Then the organic layer was dried over $MgSO_4$ and concentrated. The residue was then purified by flash column chromatography on silica gel (eluent: hexane and ethyl acetate, 5:1). Compounds **6a** (120 mg) and **6b** (117 mg) were obtained in 39% and 34% isolated yield, respectively.

6a: ^{1}H NMR (500 MHz, $CDCl_3$) δ (ppm): 9.89 (s, 1H), 7.74 (d, J = 4.0, 1H), 7.63 (d, J = 8.8 Hz, 2H), 7.33 (d, J = 3.9, 1H), 7.12 (d, J = 8.8 Hz, 2H), 5.25 (s, 2H), 3.52 (s, 3H), 1.27 (s, 3H).
6b: ^{1}H NMR (500 MHz, $CDCl_3$) δ (ppm): 9.89 (s, 1H), 7.74 (d, J = 3.9, 1H), 7.34 (d, J = 4.0, 1H), 7.25 (d, J = 2.0 Hz, 1H), 7.22 (s, 1H), 7.18 (d, J = 1.9 Hz, 1H), 5.29 (s, 2H), 3.97 (s, 3H), 3.55 (s, 3H). ^{13}C NMR (126 MHz, $CDCl_3$) δ (ppm): 182.94, 154.62, 150.30, 148.04, 142.14, 137.78, 132.40, 128.82, 127.76, 123.76, 119.68, 116.69, 110.23, 95.58, 56.59, 56.30, 29.95.

*Compound **8a** and **8b***: **6a** (90 mg, 0.36 mmol, 1.0 eq) or **6b** (100 mg, 0.36 mmol, 1.0 eq) was added to a solution of compound **7** (diethyl (4-(dimethylamino)benzyl)phosphonite) (100 mg, 0.36 mmol, 1.0 eq) in DMF (5 mL). Then sodium methoxide (59 mg, 1.09 mmol, 3.0 eq) was added to the reaction vessel in portions, and the reaction mixture was stirred at room temperature overnight. After which, dichloromethane was added to the reaction mixture, and the organic layer was washed with brine 3 times. The organic layer was then dried over $MgSO_4$ and concentrated. The residue was purified by flash column chromatography on silica gel (eluent: hexane and ethyl acetate, 3:1). Compound **8a** (41 mg, 0.11 mmol) and **8b** (43 mg) were obtained in 31% and 30% yield, respectively.

8a: ^{1}H NMR (500 MHz, $CDCl_3$) δ (ppm): 7.55 (d, J = 8.8 Hz, 2H), 7.40 (d, J = 8.7 Hz, 2H), 7.12 (d, J = 3.7 Hz, 1H), 7.08 (d, J = 8.7 Hz, 2H), 7.03 (d, J = 16.0 Hz, 2H), 6.95 (d, J = 3.7 Hz, 1H), 6.89 (d, J = 16.0 Hz, 1H), 6.74 (d, J = 8.6 Hz, 4H), 5.23 (s, 2H), 3.53 (s, 4H), 3.02 (s, 6H).
8b: ^{1}H NMR (500 MHz, $CDCl_3$) δ (ppm): 7.45-7.37 (m, 2H), 7.23-7.15 (m, 3H), 7.14 (d, J = 3.6 Hz, 1H), 7.04 (d, J = 16.0 Hz, 1H), 6.96 (d, J = 3.7 Hz, 1H), 6.90 (d, J = 16.0 Hz, 1H), 6.79-6.71 (m, 2H), 5.40-5.27 (m, 2H), 3.98 (s, 3H), 3.57 (s, 3H), 3.19-2.83 (s, 6H).

*Compound **9a** and **9b***: Concentrated hydrogen chloride (2 mL) was added to a solution of **8a** (60 mg, 0.164 mmol) or **8b** (42 mg, 0.106 mmol) in dichloromethane (5 mL) and methanol (5 mL). The resulting mixture was stirred at room temperature overnight. The solvent was then removed under vacuum, and dichloromethane was added to the residue. The organic solution was washed with NaHCO3 solution and brine. The organic layer was dried over $MgSO_4$ and concentrated. The residue was then purified by flash column chromatography on silica gel (eluent: hexane and ethyl acetate, 1:1). Compound **9a** (50 mg) and **9b** (30 mg) were obtained in 92% and 85% isolated yield, respectively.

9a: ^{1}H NMR (500 MHz, $(CD_3)_2CO$) δ (ppm): 7.51 (d, J = 8.3 Hz, 2H), 7.41 (d, J = 8.5, 2H), 7.17 (d, J = 3.7 Hz, 1H), 7.12 (d, J = 16.1 Hz, 1H), 6.99 (d, J = 3.7 Hz, 1H), 6.92-6.83 (m, 3H), 6.74 (d, J = 8.4 Hz, 2H), 2.97 (s, 6H).
9b: ^{1}H NMR (500 MHz, $CDCl_3$) δ (ppm): 7.40 (d, J = 8.7 Hz, 2H), 7.17 (dd, J = 8.2, 2.0 Hz, 1H), 7.12-7.08 (m, 2H), 7.04 (d, J = 16.0 Hz, 1H), 6.98-6.93 (m, 2H), 6.88 (d, J = 16.0 Hz, 1H), 6.74 (d, J = 8.4 Hz, 2H), 3.99 (s, 3H), 3.02 (s, 6H).

*Compound **ZY-12-MT and ZY-12-OMe***: Paraformaldehyde (5 mg, 0.167 mmol) was added to a solution of 1,4-dimethyl-1,4,7-triazacyclononane (20 mg, 0.127 mmol) in MeCN (10 mL), and the solution was refluxed for 30 min. Then a solution of compound **9a** (33 mg, 0.103 mmol) or **9b** (30 mg, 0.085 mmol) in MeCN (5 mL) was added to the reaction mixture, and it was further refluxed overnight. The solvent was then removed under vacuum, and the resulting residue was purified by C-18 reversed-phase column (eluent: MeCN/H2O with 0.1% TFA, gradient wash from 10:90 to 30:70 and 50:50). The crude product collected from the reversed-phase column was then neutralized with saturated NaHCO3 solution, extracted by dichloromethane (3x50 mL). The organic solvent was dried over MgSO4 and concentrated to yield the final product. **ZY-12-MT** and **ZY-12-OMe** were obtained in 32 % and 24 % isolated yield, respectively.

ZY-12-MT: ^{1}H NMR (500 MHz, $CDCl_3$) δ (ppm): 7.34 (d, J = 8.0 Hz, 1H), 7.29 (d, J = 8.5 Hz, 2H), 7.17 (s, 1H), 7.09 (d, J = 7.8 Hz, 1H), 6.97 (d, J = 3.6 Hz, 1H), 6.92 (d, J = 16.0 Hz, 1H), 6.83 (d, J = 3.6 Hz, 1H), 6.75 (d, J = 16.0 Hz, 1H), 6.63 (d, J = 8.5 Hz, 2H), 3.79 (s, 2H), 2.92 (s, 6H), 2.80 (m, 4H), 2.67 (m, 8H), 2.46 (s, 6H). ^{13}C NMR (126 MHz, $CDCl_3$) δ (ppm): 148.99, 141.05, 140.98, 128.85, 128.80, 126.94, 126.85, 126.31, 125.31, 124.86, 124.51, 124.48, 122.69, 120.84, 116.91, 116.10, 111.45, 111.07, 39.44, 30.91, 28.69, 28.35, 21.68, 13.11.
HRMS: calculated exact mass = 491.2845 for $[M+H]^+$, found 491.2821.

ZY-12-OMe: ^{1}H NMR (500 MHz, $CDCl_3$) δ (ppm): 7.37 (d, J = 8.6 Hz, 2H), 7.06 (d, J = 3.7 Hz, 1H), 7.04 (d, J = 2.0 Hz, 1H), 7.01 (d, J = 16.0 Hz, 1H), 6.92 (d, J = 3.7 Hz, 1H), 6.89 (d, J = 2.0 Hz, 1H), 6.85 (d, J = 16.0 Hz, 1H), 6.72 (d, J = 8.7 Hz, 2H), 3.95 (s, 3H), 3.87 (s, 2H), 3.00 (m, 10H), 2.81-2.71 (m, 4H), 2.63 (m, 4H), 2.42 (s, 6H). ^{13}C NMR (126 MHz, $CDCl_3$) δ (ppm): 148.99, 147.23, 146.58, 141.25, 141.07, 127.00, 126.31, 124.82, 124.43, 124.14, 122.22, 120.98, 117.20, 116.87, 111.44, 107.39, 59.69, 57.25, 56.92, 54.98, 52.26, 45.58, 39.42, 28.68.
HRMS: calculated exact mass = 521.2950 for $[M+H]^+$, found 521.2943.

*Compound **ZY-12-DT***: Paraformaldehyde (5 mg, 0.167 mmol) was added to a solution of 1,4-dimethyl-1,4,7-triazacyclononane (20 mg, 0.127 mmol) in MeCN (10 mL), and was heated under reflux for 30 min. Then a solution of compound **9a** (33 mg, 0.103 mmol) or **9b** (30 mg, 0.085

mmol) in MeCN (5 mL) was added to the reaction mixture, and it was further refluxed overnight. The solvent was removed under vacuum, and the resulting residue was purified by C-18 reversed-phase column (eluent: MeCN/H2O with 0.1% TFA, gradient wash from 10:90 to 30:70 and 50:50). The crude product collected from the reversed-phase column was then neutralized with saturated NaHCO3 solution, extracted by dichloromethane (3x50mL). The organic solvent was dried over MgSO4 and concentrated to yield the final product. **ZY-12-DT** were obtained in 19 % isolated yield.

ZY-12-DT: ^{1}H NMR (500 MHz, $CDCl_3$) δ (ppm): 7.37 (d, J = 8.8 Hz, 2H), 7.30 (s, 2H), 7.06 (d, J = 3.7 Hz, 1H), 7.01 (d, J = 16.1 Hz, 1H), 6.92 (d, J = 3.7 Hz, 1H), 6.84 (d, J = 16.0 Hz, 1H), 6.72 (d, J = 8.9 Hz, 2H), 3.88 (s, 4H), 3.00 (s, 6H), 3.00-2.92 (m, 8H), 2.89 -2.77 (m, 16H), 2.45 (s, 12H). ^{13}C NMR (126 MHz, $CDCl_3$) δ (ppm): 157.18, 150.19, 142.44, 141.52, 130.05, 128.39, 127.49, 126.63, 126.00, 125.41, 125.25, 124.16, 122.17, 117.82, 112.56, 58.57, 56.09, 52.60, 45.38, 40.55, 32.04, 31.05, 29.78, 29.39, 22.81, 14.25.
HRMS: calculated exact mass = 660.4424 for $[M+H]^+$, found 660.4427.

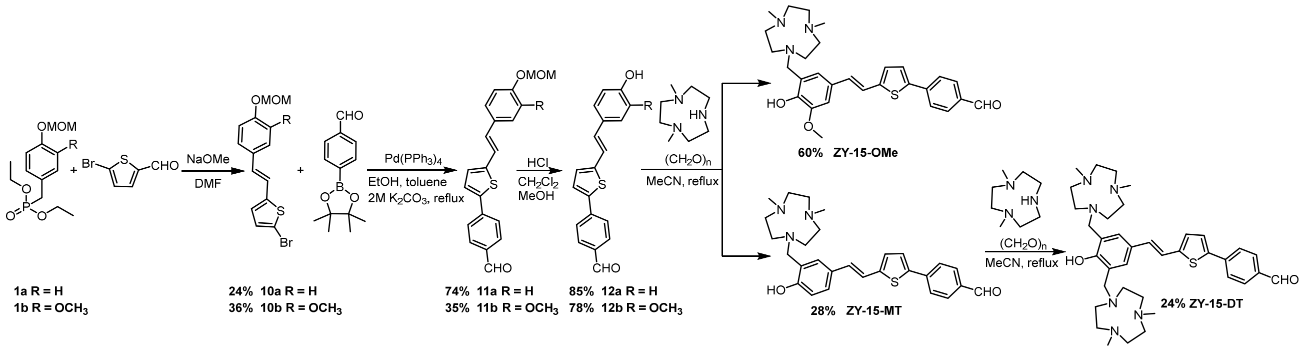

Scheme S3. Synthetic route for ZY-15-MT, ZY-15-DT and ZY-15-OMe.

*Compound **10a** and **10b***: 5-bromothiophene-2-carbaldehyde (265 mg, 1.39 mmol, 1.0 eq) was added to a solution of compound **1a** (400 mg, 1.39 mmol, 1 eq) or **1b** (440 mg, 1.39 mmol, 1.0 eq) in DMF (5ml). Then sodium methoxide (234 mg, 2.10 mmol, 1.5 eq) was added to the reaction vessel in portions, and the reaction mixture was stirred at room temperature for 24 hr. After which, dichloromethane was added to the reaction mixture, and the organic layer was washed with brine 3 times. The organic layer was then dried over $MgSO_4$ and concentrated. The residue was purified by flash column chromatography on silica gel (eluent: hexane and ethyl acetate, 5:1). Compound **10a** (108 mg) and **10b** (178 mg) were obtained in 24% and 36% isolated yield, respectively.

10a: ^{1}H NMR (500 MHz, $CDCl_3$) δ (ppm): 7.38 (d, J = 8.4 Hz, 2H), 7.07-6.96 (m, 3H), 6.95 (d, J = 3.8 Hz, 1H), 6.81-6.74 (m, 2H), 5.20 (s, 2H), 3.50 (s, 3H), 2.18 (s, 3H). ^{13}C NMR (500 MHz, $CDCl_3$) δ (ppm): 157.32, 145.06, 130.72, 128.55, 127.83, 125.97, 119.87, 116.73, 94.60, 56.31, 31.19.
10b: ^{1}H NMR (500 MHz, $CDCl_3$) δ (ppm): 7.08 (dd, J = 8.4, 1.2 Hz, 1H), 7.02- 6.92 (m, 3H), 6.91 (dd, J = 3.9, 1.3 Hz, 1H), 6.78-6.69 (m, 2H), 5.21 (s, 2H), 3.90 (s, 3H), 3.49 (s, 3H).

*Compound **11a** and **11b***: (4-(4,4,5,5-tetramethyl-1,3,2-dioxaborolan-2-yl)benzaldehyde) (71mg, 0.31 mmol, 1.0 eq) and compound **10a** (100 mg, 0.31 mmol, 1.0 eq) or **10b** (70 mg, 0.30 mmol,

1.0 eq) was dissolved in a mixture of solvent of 10 ml ethanol and 10 ml toluene. 2 ml of an aqueous K_2CO_3 (2M) solution was added to the reaction mixture followed by the addition of $Pd(PPh_3)_4$ (18 mg, 0.015 mmol, 0.05 eq). The mixture was then stirred overnight under reflux. The solvent was removed under vacuum, and the residue was washed with brine and extracted with dichloromethane. Then the organic layer was collected, dried over $MgSO_4$, and concentrated. The residue was purified by flash column chromatography on silica gel (eluent: hexane and ethyl acetate, 6:1). The compounds **11a** (80 mg) and **11b** (44 mg) were obtained in 74% and 35% isolated yield, respectively.

11a: ^{1}H NMR (400 MHz, $CDCl_3$) δ (ppm): 10.00 (s, 1H), 7.89 (d, J = 8.4 Hz, 2H), 7.75 (d, J = 8.4 Hz, 2H), 7.42 (d, J = 8.7 Hz, 2H), 7.38 (d, J = 3.8 Hz, 1H), 7.09 (d, J = 16.1 Hz, 1H), 7.06-7.01 (m, 3H), 6.95 (d, J = 16.1 Hz, 1H), 5.20 (s, 2H), 3.50 (s, 3H).
11b: ^{1}H NMR (400 MHz, $CDCl_3$) δ (ppm): 9.99 (s, 1H), 7.88 (d, J = 8.2 Hz, 2H), 7.74 (d, J = 8.3 Hz, 2H), 7.38 (d, J = 3.8 Hz, 1H), 7.14 (d, J = 8.1 Hz, 1H), 7.10 (d, J = 16.0 Hz, 1H), 7.04 (m, 4H), 6.94 (d, J = 16.0 Hz, 1H), 5.26 (s, 2H), 3.95 (s, 3H), 3.53 (s, 3H).

*Compound **12a** and **12b***: Concentrated hydrogen chloride (2 mL) was added to a solution of **11a** (30 mg, 0.086 mmol) or **11b** (40 mg, 0.105 mmol) in a mixture of dichloromethane (5 mL) and methanol (5 mL). The resulting mixture was stirred at room temperature for 12 h. The solvent was removed under vacuum, and dichloromethane was added to the residue. The organic solution was washed with $NaHCO_3$ solution. The organic layer was dried over $MgSO_4$ and concentrated. The residue was then purified by flash column chromatography on silica gel (eluent: hexane and ethyl acetate, 3:1). Compound **12a** (22 mg) and **12b** (27 mg) were obtained in 85% and 78% isolated yield, respectively.

12a: ^{1}H NMR (400 MHz, $(CD_3)_2CO$) δ (ppm): 10.02 (s, 1H), 7.94 (d, J = 8.3 Hz, 2H), 7.89 (d, J = 8.3 Hz, 2H), 7.59 (d, J = 3.8 Hz, 1H), 7.45 (d, J = 8.6 Hz, 3H), 7.23 (d, J = 16.2 Hz, 1H), 7.16 (d, J = 3.9 Hz, 1H), 7.01 (d, J = 16.1 Hz, 1H), 6.85 (d, J = 8.6 Hz, 2H).
12b: ^{1}H NMR (500 MHz, $CDCl_3$) δ (ppm): 10.02 (s, 1H), 7.93-7.88 (m, 2H), 7.79-7.74 (m, 2H), 7.40 (d, J = 3.7 Hz, 1H), 7.11-7.06 (m, 2H), 7.03 (m, 2H), 6.98-6.91 (m, 2H), 3.98 (s, 3H). ^{13}C NMR (126 MHz, $CDCl_3$) δ (ppm): 191.42, 146.80, 145.99, 144.93, 140.41, 140.01, 134.93, 130.52, 129.58, 129.29, 126.87, 125.75, 125.60, 120.60, 119.35, 114.73, 108.21, 55.96.

*Compound **ZY-15-MT** and **ZY-15-OMe***: Paraformaldehyde (3 mg, 0.100 mmol) was added to a solution of 1,4-dimethyl-1,4,7-triazacyclononane (10 mg, 0.063 mmol) in MeCN (10 mL), and the solution was refluxed for 30 min. Then a solution of compound **12a** (22 mg, 0.071 mmol) or **12b** (20 mg, 0.059 mmol) in MeCN (5mL) was added to the reaction mixture, and it was heated under reflux overnight. The solvent was removed under vacuum, and the resulting residue was purified by C-18 reversed-phase column (eluent: MeCN/H_2O with 0.1% TFA, gradient wash from 10:90 to 30:70 to 50:50). The crude product collected from the reversed-phase column was then neutralized with saturated $NaHCO_3$ solution, extracted by dichloromethane (3x50 mL). The organic solvent was dried over $MgSO_4$ and concentrated to yield the final products. Compounds **ZY-15-MT** (9 mg) and **ZY-15-OMe** (18 mg) were obtained in 28% and 60% isolated yield, respectively.

ZY-15-MT: ^{1}H NMR (500 MHz,$CDCl_3$) δ (ppm): 9.91 (s, 1H), 7.83-7.74 (d, J = 8.3 Hz, 2H), 7.65 (d, J = 8.2 Hz, 2H), 7.28 (d, J = 3.8 Hz, 1H), 7.23 (dd, J = 8.3, 2.2 Hz, 1H), 7.19 (s, 1H), 7.07 (d, J = 2.2 Hz, 1H), 7.00-6.89 (m, 2H), 6.82 (d, J = 16.0 Hz, 1H), 3.78 (s, 2H), 2.80 (m, 4H), 2.64 (m, 8H), 2.44 (s, 6H). ^{13}C NMR (126 MHz, $CDCl_3$) δ (ppm): 190.35, 144.26, 139.02, 133.82, 129.45, 128.52, 126.52, 126.43, 125.53, 124.72, 124.50, 122.72, 117.49, 116.06, 58.26, 54.64, 50.40, 44.06, 30.91, 28.68, 28.35, 21.68, 13.11.
HRMS: calculated exact mass = 476.2372 for $[M+H]^+$, found 476.2369.

ZY-15-OMe: ^{1}H NMR (500 MHz, $CDCl_3$) δ (ppm): 10.01 (s, 1H), 7.90 (d, J = 8.3 Hz, 2H), 7.76 (d, J = 8.2 Hz, 2H), 7.39 (d, J = 3.8 Hz, 1H), 7.11-7.04 (m, 2H), 6.99 (d, J = 2.0 Hz, 1H), 6.95-6.81 (m, 2H), 3.96 (s, 3H), 3.87 (s, 2H), 2.96 (m, 4H), 2.91-2.77 (m, 8H), 2.51 (s, 6H). ^{13}C NMR (126 MHz, $CDCl_3$) δ (ppm): 190.35, 147.08, 143.95, 139.30, 138.97, 133.89, 129.47, 128.50, 126.93, 126.72, 125.77, 124.74, 124.55, 122.29, 119.90, 118.06, 107.43, 62.60, 55.06, 51.75, 44.29, 30.89, 29.91, 28.69, 28.35, 21.68, 13.11.
HRMS: calculated exact mass = 506.2477 for $[M+H]^+$, found 506.2464.

*Compound **ZY-15-DT***: Paraformaldehyde (5 mg, 0.167 mmol) was added to a solution of 1,4-dimethyl-1,4,7-triazacyclononane (20 mg, 0.126 mmol) in MeCN (10 mL), and the mixture was heated under reflux for 30 min. Then a solution of compound **ZY-15-MT** (33 mg, 0.069 mmol) in MeCN (5 mL) was added to the reaction mixture, and the solution mixture was refluxed for another 24 h. The solvent was removed under vacuum, and the resulting residue was purified by C-18 reversed-phase column (eluent: MeCN/H_2O with 0.1% TFA, gradient wash from 10:90 to 30:70). The crude product collected from the reversed-phase column was then neutralized with saturated $NaHCO_3$ solution, extracted by dichloromethane (3x50 mL). The organic solvent was dried over $MgSO_4$ and concentrated to yield **ZY-15-DT** (11 mg) in 24% isolated yield.

ZY-15-DT: ^{1}H NMR (500 MHz, $CDCl_3$) δ (ppm): 9.92 (s, 1H), 7.81 (d, J = 8.1 Hz, 2H), 7.67 (d, J = 8.1 Hz, 2H), 7.30 (d, J = 3.7 Hz, 1H), 7.19 (s, 1H), 7.15 (s, 1H), 7.03-6.93 (m, 2H), 6.82 (d, J = 16.0 Hz, 1H), 3.78 (s, 4H), 2.89 (m, 8H), 2.69 (m, 16H), 2.34 (s, 12H). ^{13}C NMR (126 MHz, $CDCl_3$) δ (ppm): 190.35, 156.69, 144.12, 139.01, 133.85, 129.47, 128.37, 126.40, 125.98, 125.61, 124.74, 124.54, 123.05, 117.68, 57.50, 55.65, 52.39, 44.82, 30.91, 28.68, 28.30, 21.71, 13.11.
HRMS: calculated exact mass = 645.3951 for $[M+H]^+$, found 645.3931.

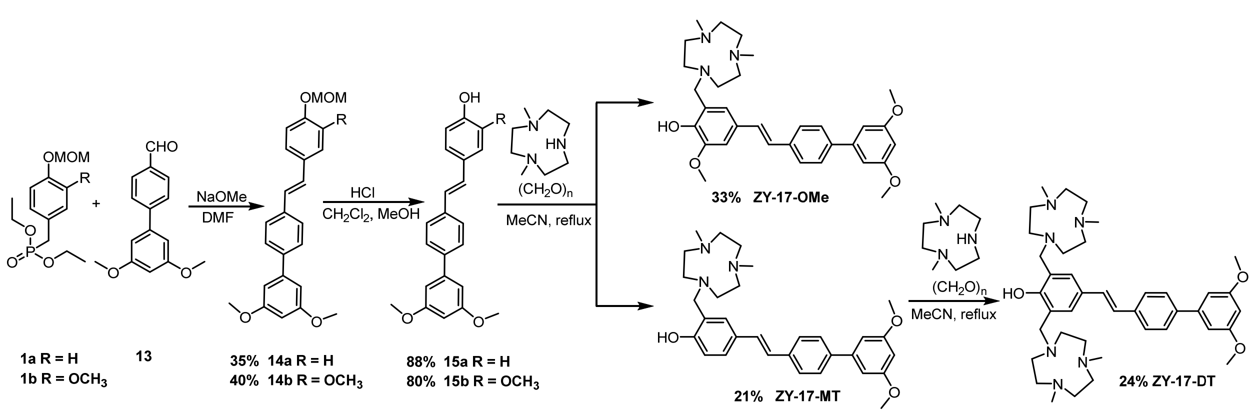

Scheme S4. Synthetic route for ZY-17-MT, ZY-17-DT and ZY-17-OMe.

*Compound **14a** and **14b***: Compound **1a** (59 mg, 0.206 mmol, 1.0 eq) or **1b** (65 mg, 0.206 mmol, 1.0 eq) was added to a solution of compound **13** (50 mg, 0.206 mmol, 1.0 eq) in DMF (5 mL). Then sodium methoxide (33 mg, 0.619 mmol, 3.0 eq) was added to the solution in portions, and the reaction mixture was stirred at room temperature overnight. Then dichloromethane was added to the mixture, and the organic solution was washed with brine 3 times. The organic layer was then dried over $MgSO_4$ and concentrated. The residue was purified by flash column chromatography on silica gel (eluent: hexane and ethyl acetate, 5:1). Compound **14a** (27 mg) and **14b** (34 mg) were obtained in 35% and 40% isolated yield, respectively.

14a: ^{1}H NMR (500 MHz, $CDCl_3$) δ (ppm): 7.53-7.44 (m, 4H), 7.42-7.36 (d, J = 8.6Hz, 2H), 7.03 (d, J = 16.4 Hz, 1H), 6.99-6.89 (m, 3H), 6.68 (d, J = 2.3 Hz, 2H), 6.39 (t, J = 2.3 Hz, 1H), 5.12 (s, 2H), 3.78 (s, 5H), 3.42 (s, 3H). ^{13}C NMR (126 MHz, $CDCl_3$) δ (ppm): 160.06, 155.91, 141.93, 138.92, 135.92, 130.23, 127.26, 126.69, 126.35, 125.62, 125.50, 115.44, 104.18, 98.26, 93.39, 55.01, 54.40.
14b: ^{1}H NMR (500 MHz, $CDCl_3$) δ (ppm): 7.66-7.53 (m, 4H), 7.18 (d, J = 8.2 Hz, 1H), 7.15-7.02 (m, 4H), 6.83-6.77 (s, 2H), 6.51 (s, 1H), 5.29 (s, 2H), 3.99 (s, 3H), 3.88 (s, 6H), 3.57 (s, 3H). ^{13}C NMR (126 MHz, $CDCl_3$) δ (ppm): 161.38, 150.16, 146.67, 143.17, 140.30, 137.08, 132.29, 128.80, 127.65, 127.08, 126.96, 120.10, 116.66, 109.67, 105.47, 99.57, 95.74, 56.51, 56.19, 55.66.

*Compound **15a, 15b***: Concentrated hydrogen chloride (2 mL) was added to a solution of **14a** (50 mg, 0.133 mmol) or **14b** (65 mg, 0.160 mmol) in a mixture of dichloromethane (5 mL) and methanol (5 mL). The resulting mixture was stirred at room temperature overnight. The solvent was then removed under vacuum, and dichloromethane was added to the residue. The organic solution was washed with $NaHCO_3$ solution and brine. The organic layer was dried over $MgSO_4$ and concentrated. The residue was then purified by flash column chromatography on silica gel (eluent: hexane and ethyl acetate, 3:1). Compound **15a** (39 mg) and **15b** (46 mg) were obtained in 88% and 80% isolated yield, respectively.

15a: ^{1}H NMR (500 MHz, $CDCl_3$) δ (ppm): 7.65-7.54 (m, 4H), 7.46 (dd, J = 8.0, 4.0 Hz, 2H), 7.13 (d, J = 16.2 Hz, 1H), 7.03 (d, J = 16.3 Hz, 1H), 6.88 (d, J = 8.5 Hz, 2H), 6.79 (d, J = 2.3 Hz, 2H), 6.51 (t, J = 2.3 Hz, 1H), 3.89 (s, 6H). ^{13}C NMR (126 MHz, $CDCl_3$) δ (ppm): 161.34, 155.63, 143.24, 140.13, 137.26, 130.56, 128.59, 128.23, 127.64, 126.86, 126.36, 115.93, 105.50, 99.55, 55.70.
15b: ^{1}H NMR (126 MHz, $CDCl_3$) δ (ppm): 7.59 (q, J = 8.4 Hz, 4H), 7.15-7.06 (m, 3H), 7.02 (d, J = 16.2 Hz, 1H), 6.96 (d, J = 7.9 Hz, 1H), 6.80 (d, J = 2.3 Hz, 2H), 6.51 (t, J = 2.3 Hz, 1H), 3.98 (s, 3H), 3.89 (s, 6H). ^{13}C NMR (126 MHz, $CDCl_3$) δ (ppm): 160.05, 145.72, 144.65, 141.90, 138.81, 135.92, 128.93, 127.76, 126.34, 125.54, 124.89, 119.52, 113.58, 107.23, 104.16, 98.24, 54.88, 54.39.

*Compound **ZY-17-MT** and **ZY-17-OMe***: Paraformaldehyde (5 mg, 0.167 mmol) was added to a solution of 1,4-dimethyl-1,4,7-triazacyclononane (20 mg, 0.126 mmol) in MeCN (10 mL), and the solution mixture was heated under reflux for 30 min. Then a solution of compound **15a** (30 mg, 0.09 mmol) or **15b** (40 mg, 0.11 mmol) in MeCN (5 mL) was added to the reaction mixture, and it was refluxed overnight. The solvent was removed under vacuum, and the resulting residue was purified by C-18 reversed-phase column (eluent: MeCN/H_2O with 0.1% TFA, gradient wash from 10:90 to 30:70 to 50:50). The crude product collected from the reversed-phase column was then neutralized with saturated $NaHCO_3$ solution, extracted by dichloromethane (3x50 mL). The

organic solvent was dried over $MgSO_4$ and concentrated to yield the final product. Compound **ZY-17-MT** (9 mg) and **ZY-17-OMe** (19 mg) were obtained in 21% and 33% isolated yield, respectively.

ZY-17-MT: ^{1}H NMR (500 MHz, $CDCl_3$) δ (ppm): 7.47 (q, J = 8.3 Hz, 4H), 7.33-7.25 (m, 1H), 7.15-7.05 (m, 2H), 7.01-6.94 (m, 1H), 6.89 (d, J = 16.3 Hz, 1H), 6.68 (d, J = 2.3 Hz, 2H), 6.39 (t, J = 2.2 Hz, 1H), 3.79 (s, 6H), 3.58 (s, 2H), 2.85 (m, 8H), 2.69 (m, 4H), 2.58 (s, 6H). ^{13}C NMR (126 MHz, $CDCl_3$) δ (ppm): 161.21, 158.54, 143.17, 139.72, 137.44, 128.97, 128.29, 127.48, 127.33, 126.59, 125.11, 123.67, 116.96, 105.31, 99.38, 60.63, 57.57, 55.57, 53.04, 46.31.
HRMS: calculated exact mass = 502.3070 for $[M+H]^+$, found 502.3051.

ZY-17-OMe: ^{1}H NMR (500 MHz, $CDCl_3$) δ (ppm): 7.57 (q, J = 8.4 Hz, 4H), 7.11-7.04 (m, 2H), 6.99 (d, J = 16.2 Hz, 1H), 6.90 (d, J = 1.9 Hz, 1H), 6.77 (d, J = 2.3 Hz, 2H), 6.49 (t, J = 2.3 Hz, 1H), 3.96 (s, 3H), 3.88 (s, 6H), 3.00 (m, 4H), 2.90 (m, 8H), 2.57 (s, 6H). ^{13}C NMR (126 MHz, $CDCl_3$) δ (ppm): 161.11, 147.94, 142.89, 139.91, 136.87, 128.72, 127.41, 126.57, 125.95, 123.48, 121.52, 108.65, 105.20, 98.94, 61.64, 58.27, 56.14, 55.45, 54.72, 53.73, 51.50, 44.19, 31.94, 30.94, 29.67, 22.70, 14.14.
HRMS: calculated exact mass = 532.3175 for $[M+H]^+$, found 532.3163.

*Compound **ZY-17-DT***: Paraformaldehyde (5 mg, 0.167 mmol) was added to a solution of 1,4-dimethyl-1,4,7-triazacyclononane (20 mg, 0.126 mmol) in MeCN (10 mL), and the mixture was heated under reflux for 30 min. Then a solution of compound **ZY-17-MT** (23 mg, 0.046 mmol) in MeCN (5 mL) was added to the reaction mixture, and it was refluxed for another 24 h. The solvent was removed under vacuum, and the resulting residue was purified by C-18 reversed-phase column (eluent: MeCN/H_2O with 0.1% TFA, gradient wash from 10:90 to 30:70). The crude product collected from the reversed-phase column was then neutralized with saturated $NaHCO_3$ solution, extracted by dichloromethane (3x50 mL). The organic solvent was dried over $MgSO_4$ and concentrated to yield **ZY-17-DT** (7 mg) in 24% isolated yield.

ZY-17-DT: ^{1}H NMR (500 MHz, $CDCl_3$) δ (ppm): 7.47 (q, J = 8.3 Hz, 4H), 7.19 (s, 2H), 6.98 (d, J = 16.2 Hz, 1H), 6.90 (d, J = 16.3 Hz, 1H), 6.68 (d, J = 2.3 Hz, 2H), 6.39 (t, J = 2.3 Hz, 1H), 3.77 (m, 10H), 2.87 (m, 8H), 2.67 (m, 16H), 2.32 (s, 12H). ^{13}C NMR (126 MHz, $CDCl_3$) δ (ppm): 160.06, 156.24, 141.96, 138.64, 136.17, 128.19, 127.68, 126.61, 126.35, 126.25, 125.72, 125.44, 124.15, 123.19, 104.15, 98.23, 57.54, 55.82, 54.41, 52.58, 44.98, 28.68.
HRMS: calculated exact mass = 671.4649 for $[M+H]^+$, found 671.4651.

4. Optical properties

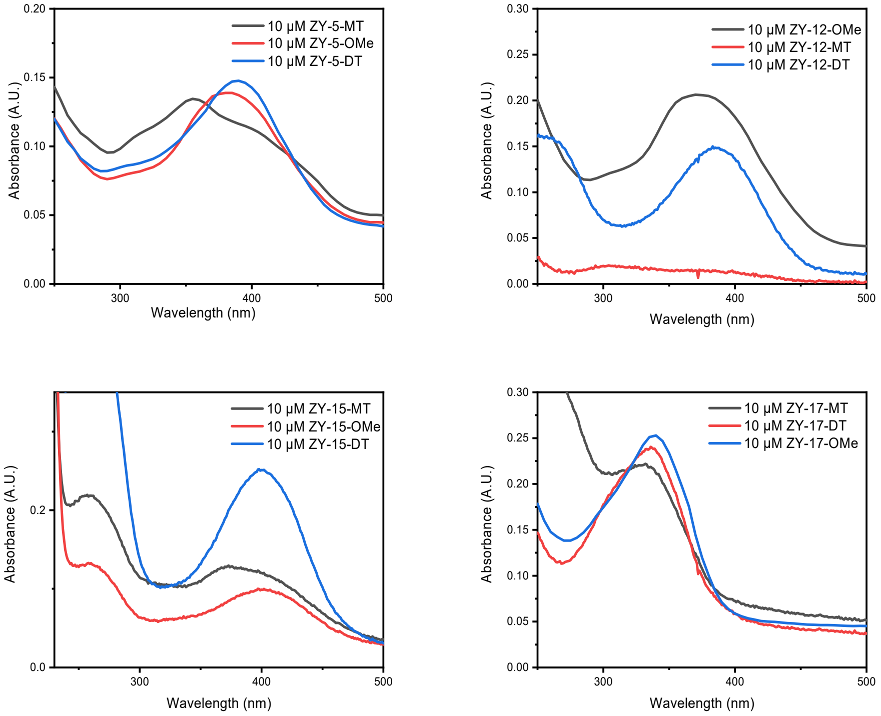

Figure S1. UV-vis spectra of 10 μM compounds in PBS.

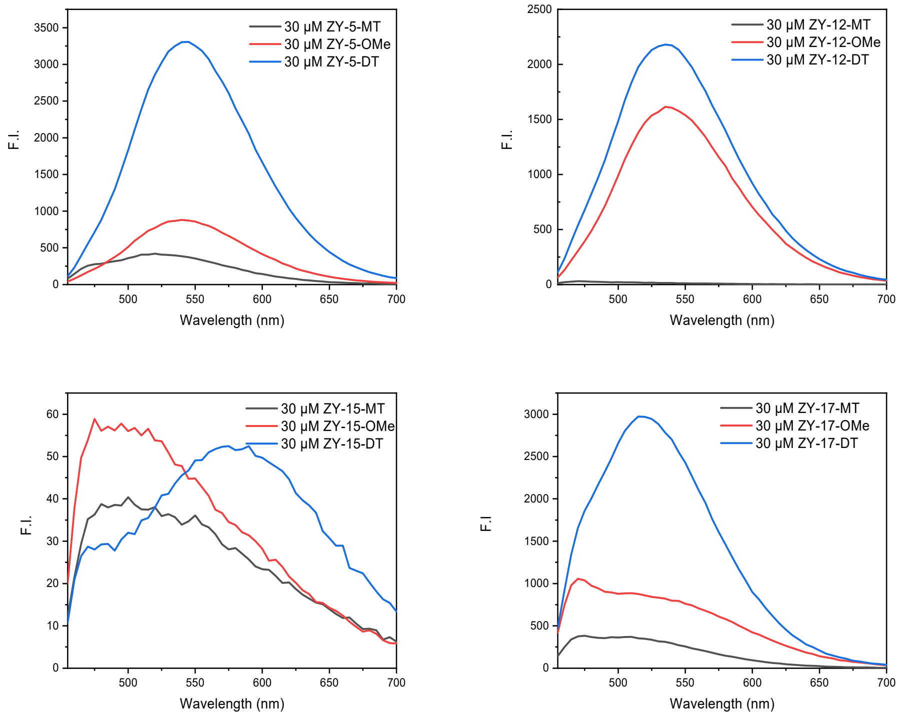

Figure S2. Fluorescence spectra of 30 μM compounds in PBS.

Table S1. Summarized optical properties

Compounds	λ_{ex} (nm)	λ_{em} (nm)
ZY-5-OMe	385	545
ZY-5-MT	355	530
ZY-5-DT	390	545
ZY-12-OMe	370	540
ZY-12-MT	380	445
ZY-12-DT	380	540
ZY-15-OMe	405	500
ZY-15-MT	405	500
ZY-15-DT	405	590
ZY-17-OMe	335	470
ZY-17-MT	335	470
ZY-17-DT	335	520

5. Fluorescence turn-on studies

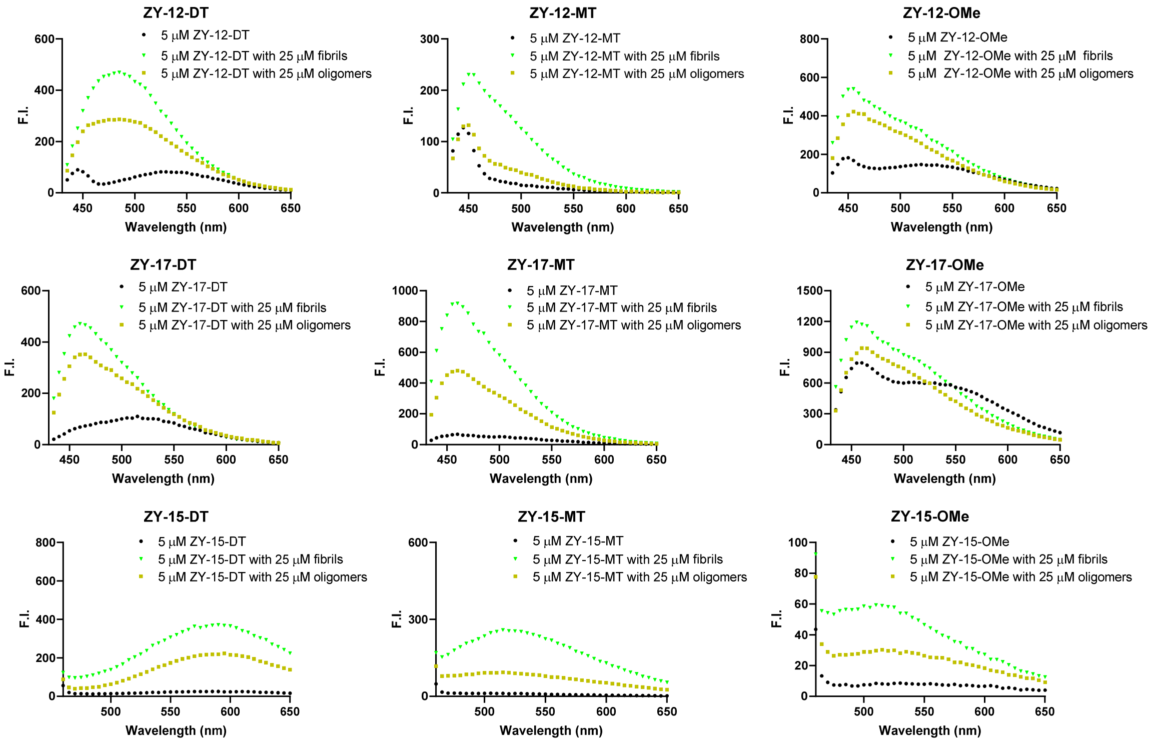

Figure S3. Fluorescence turn-on effects of amphiphilic compounds with Aβ_{42} fibrils and oligomers. [compounds] = 5 μM, [Aβ_{42} fibrils] = 25 μM, [Aβ_{42} oligomers] = 25 μM.

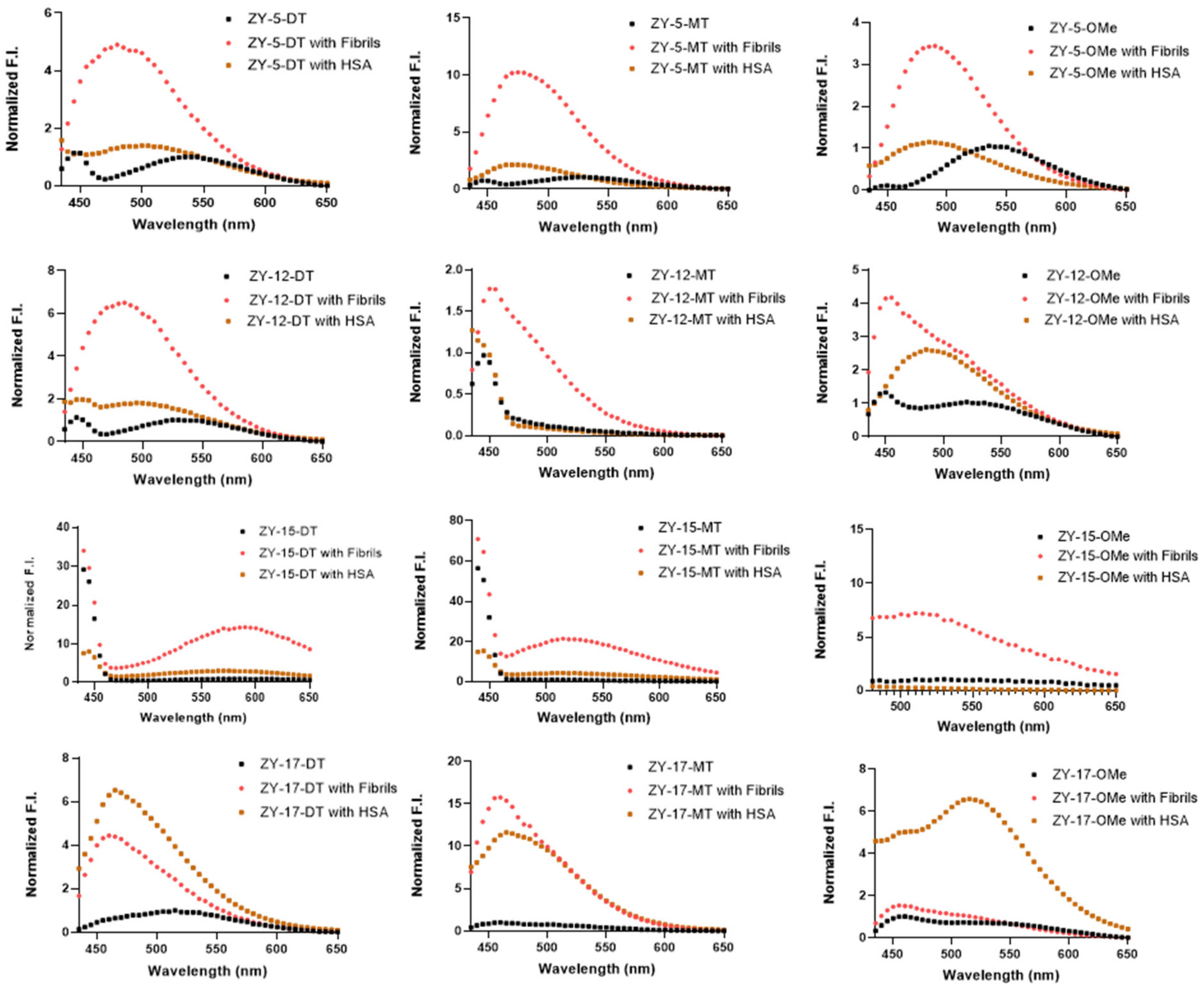

Figure S4. Fluorescence turn-on effects of amphiphilic compounds with HAS; [compound] = 5 µM, [Aβ_{42} fibrils] = 25 µM, [HSA] = 1 mg/mL.

6. K_d measurements and binding ratio

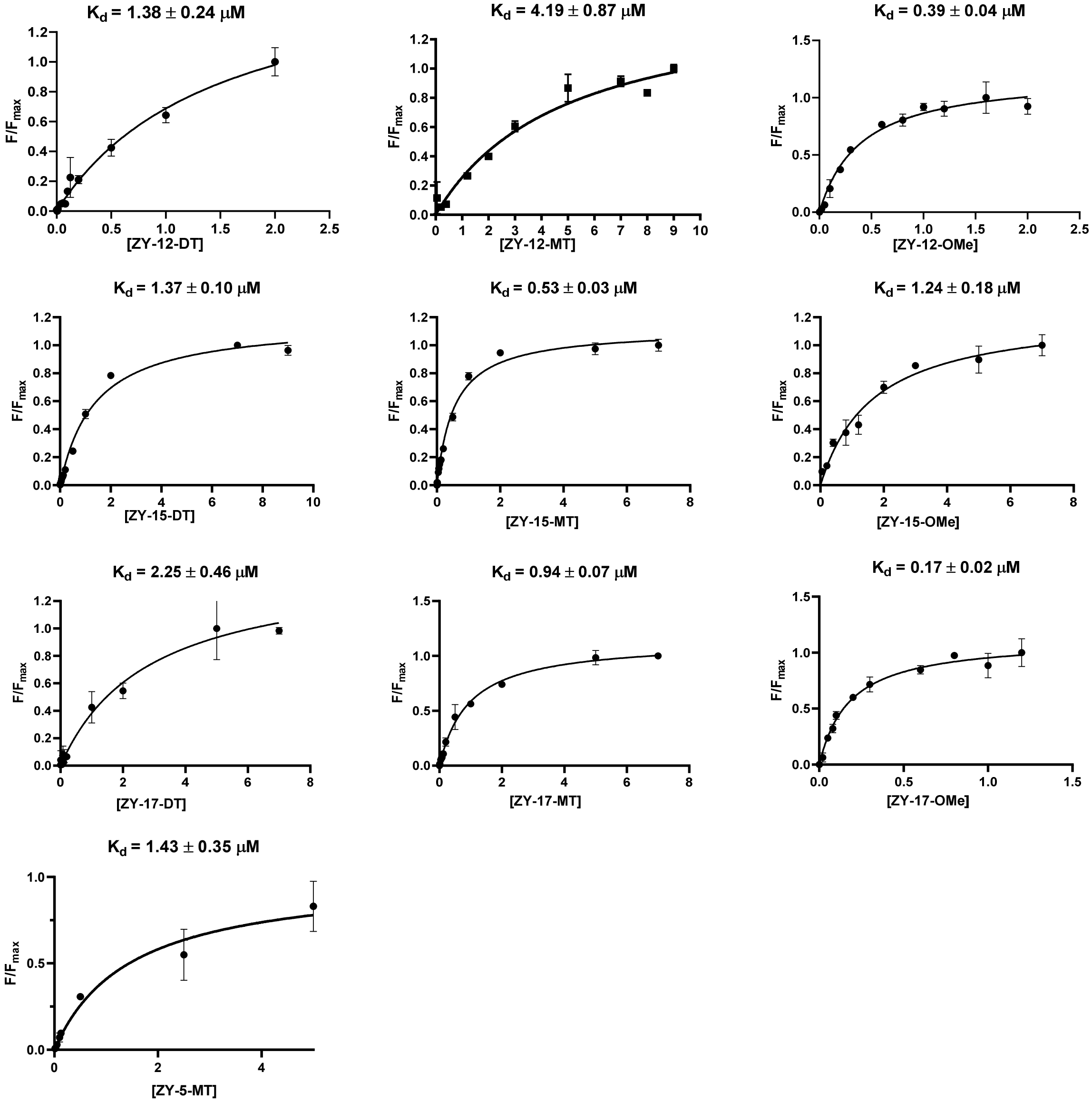

Figure S5. Binding constants of amphiphilic compounds with Aβ_{42} fibrils measured by fluorescence saturation assays. The measurements were performed in 10 mM phosphate buffered saline (PBS), pH 7.4. For ZY-12-DT, ZY-12-OMe, ZY-15-DT, ZY-15-MT, ZY-15-OMe and ZY-17-MT, [Aβ_{42} fibrils] = 5 μM. For ZY-12-MT and ZY-17-DT, [Aβ_{42} fibrils] = 8 μM, for ZY-17-OMe [Aβ_{42} fibrils] = 7 μM. Another trial for [ZY-5-MT] was also obtained, [Aβ_{42} fibrils] = 5 μM.

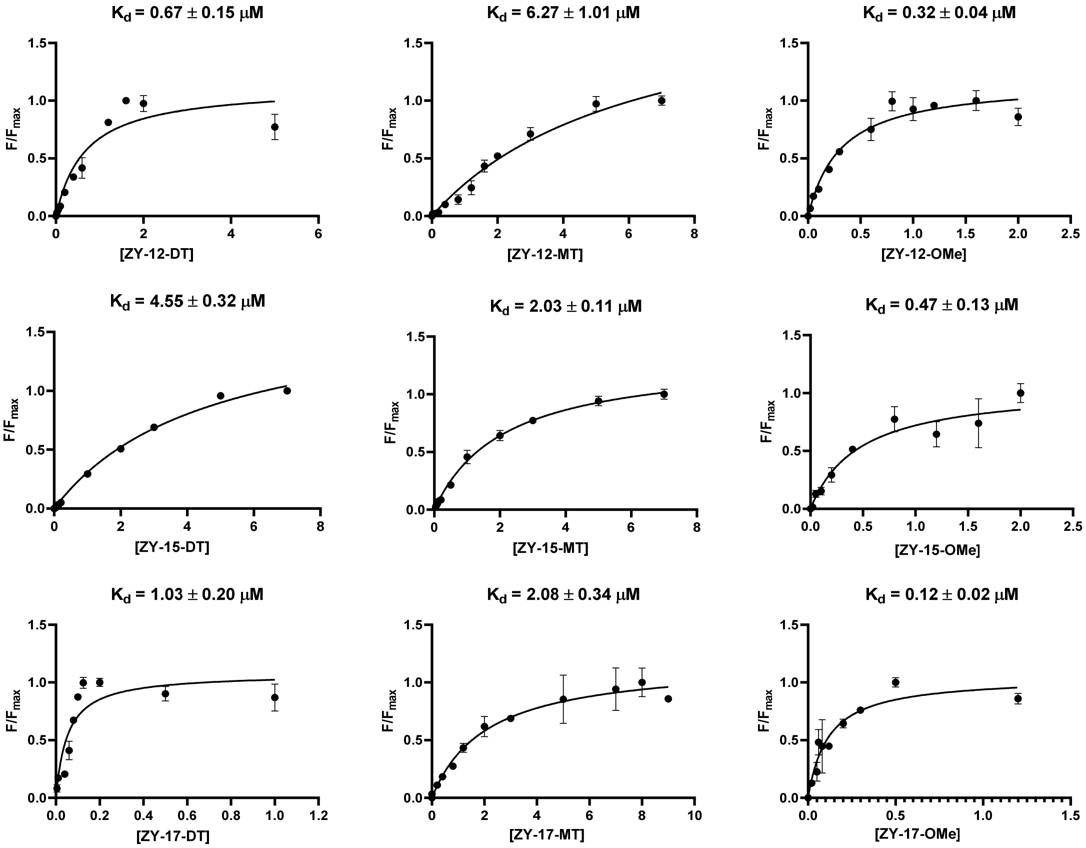

Figure S6. Binding constants of amphiphilic compounds with Aβ_{42} oligomers measured by fluorescence saturation assays. The measurements were performed in 10 mM phosphate buffered saline (PBS), pH 7.4. For ZY-12-DT, ZY-12-OMe, ZY-15-MT, ZY-15-OMe, ZY-17-DT and ZY-17-MT, [Aβ_{42} oligomers] = 5 μM. For ZY-12-MT [Aβ_{42} oligomers] = 15 μM. For ZY-17-OMe and ZY-15-DT [Aβ_{42} oligomers] = 7 μM.

Table S2. Summary of the binding ratio of amphiphilic compounds to $A\beta_{42}$ fibrils and oligomers.

Compounds	Fibrils	Oligomers
ZY-5-OMe	5.6	4.0
ZY-5-MT	2.1	4.9
ZY-5-DT	1.6	1.7
ZY-12-OMe	8.5	10.2
ZY-12-MT	1.3	1.0
ZY-12-DT	3.5	3.7
ZY-15-OMe	3.0	8.1
ZY-15-MT	10.0	2.5
ZY-15-DT	2.9	1.2
ZY-17-OMe	21.9	46.7
ZY-17-MT	5.5	2.0
ZY-17-DT	1.1	33.3

7. 5xFAD mouse brain section fluorescence imaging

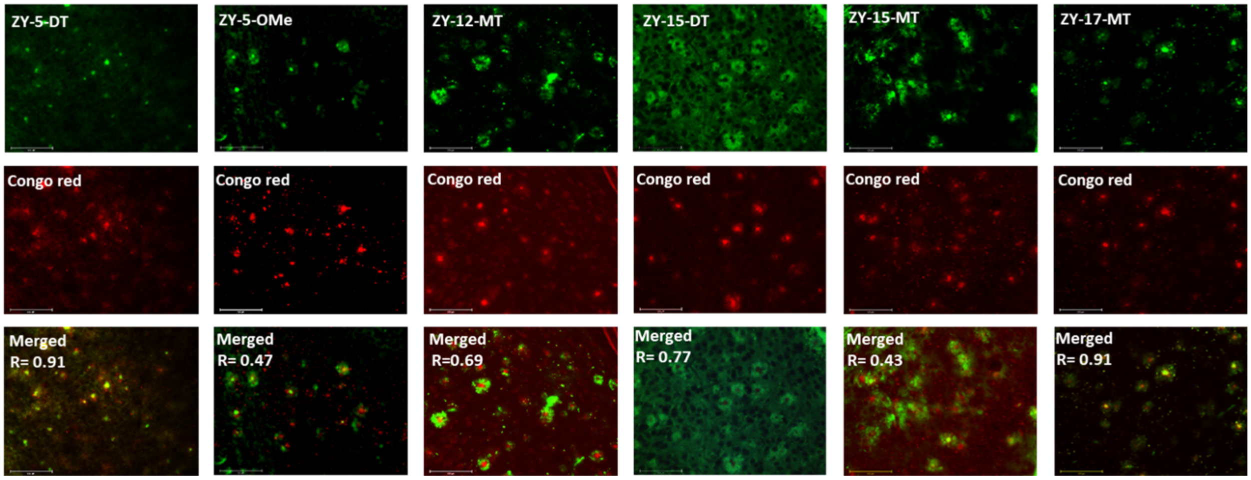

Figure S7. Fluorescence microscopic images of 5xFAD mice brain sections co-incubated with other six amphiphilic compounds (top), Congo Red (middle) and merged images (bottom, along with the Pearson's correlation coefficients R). Concentrations: [amphiphilic compounds] = 5 µM, [Congo Red] = 2.5 µM (scale bar: 125 µm).

8. Log D measurements

Table S3. Log D values for the amphiphilic compounds.

Compounds	Log D
ZY-5-OMe	1.183 ± 0.046
ZY-5-MT	0.997 ± 0.031
ZY-5-DT	1.026 ± 0.122
ZY-12-OMe	1.166 ± 0.026
ZY-12-MT	0.830 ± 0.035
ZY-12-DT	1.127 ± 0.045
ZY-15-OMe	1.053 ± 0.002
ZY-15-MT	1.107 ± 0.021
ZY-15-DT	1.157 ± 0.026
ZY-17-OMe	0.732 ± 0.019
ZY-17-MT	0.810 ± 0.079
ZY-17-DT	0.606 ± 0.032

9. Docking scores and Glide e-model energies

Table S4. Docking scores and Glide e-model energies of compounds with Aβ_{42} fibrils (PDB ID: 5OQV)

Compounds	Docking Scores	Glide e-model energies (kcal/mol)
ZY-5-MT	-7.300	-74.829
ZY-12-DT	-8.754	-84.515
ZY-15-OMe	-7.771	-77.207

Table S5. Docking scores and Glide e-model energies of compounds with Aβ_{42} oligomers (PDB ID:6RHY)

Compounds	Docking Scores	Glide e-model energies (kcal/mol)
ZY-5-MT	-4.866	-47.851
ZY-12-DT	-2.949	-55.908
ZY-15-OMe	-5.906	-50.350

10. The effect of copper chelators on Cu^{2+}-Aβ_{42} neurotoxicity

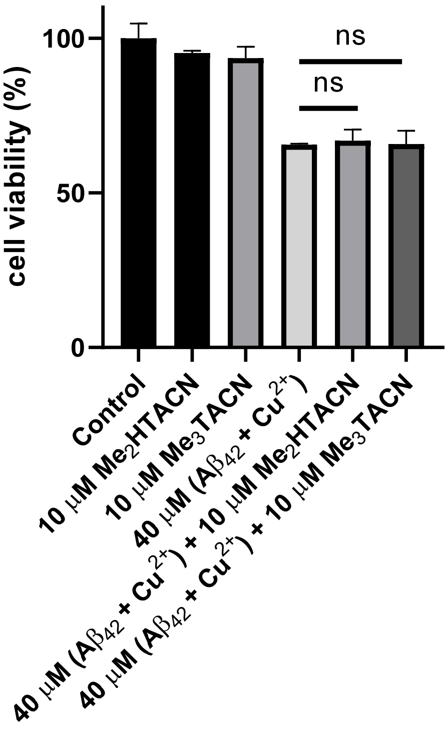

Figure S8. Cell viability results upon incubation of Neuro2A cells with 10 μM copper chelators (Me_2HTACN or Me_3TACN) in the presence or absence of 40 μM (Aβ_{42} + Cu^{2+}). The error bars represent the standard deviation from five independent experiments, and the statistical analysis was evaluated according to one-way ANOVA.

11. Cell toxicity studies in SH-SY5Y cells

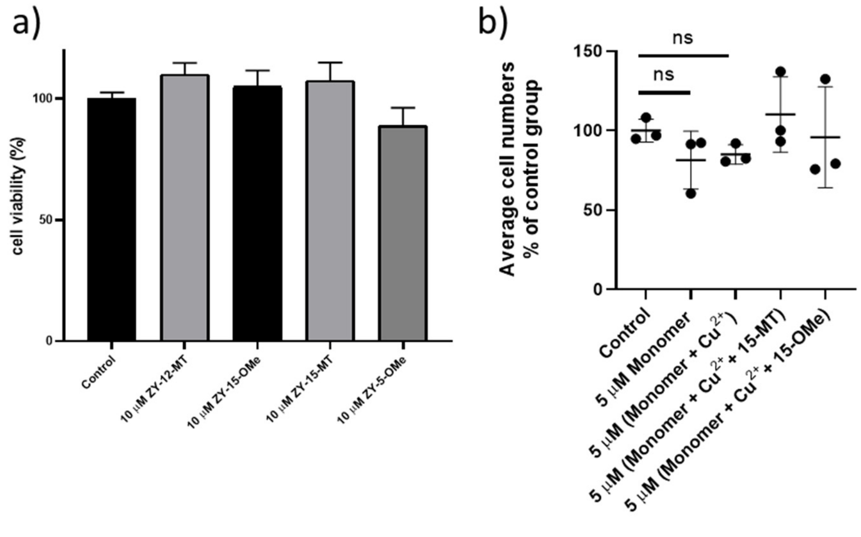

Figure S9. Cell toxicity studies in SH-SY5Y cells. a) [ZY-12-MT] = [ZY-15-OMe] = [ZY-15-MT] = [ZY-5-OMe] = 10 μM. b) Average cell numbers in presence of 5 μM $A\beta_{42}$ monomer, 5 μM ($A\beta_{42}$ monomer + Cu^{2+}), 5 μM ($A\beta_{42}$ monomer + Cu^{2+} + ZY-15-MT) and 5 μM ($A\beta_{42}$ monomer + Cu^{2+} + ZY-15-OMe).

12. Cell membrane staining

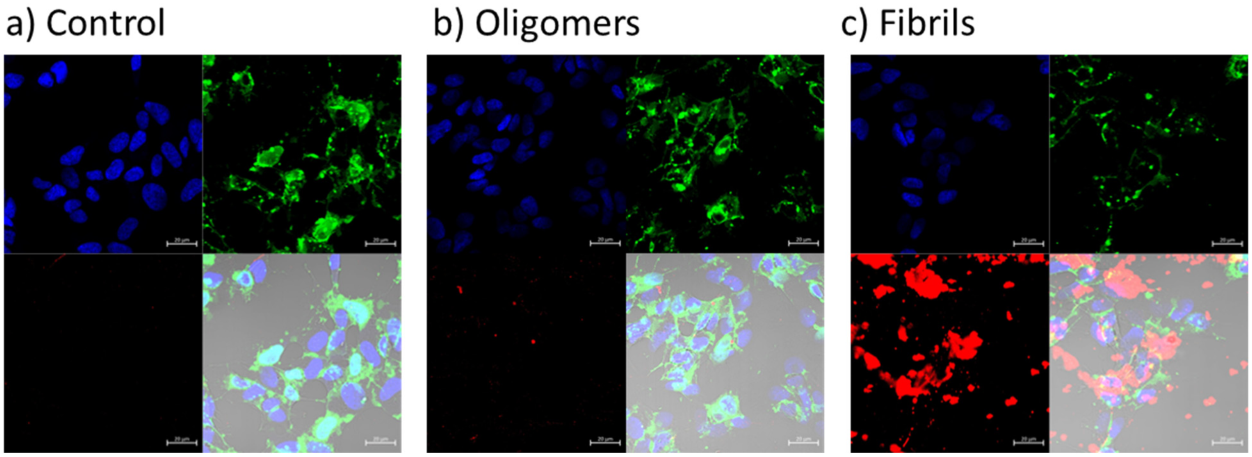

Figure S10. Cell membranes staining in the presence or absence of Aβ aggregates. a) control group no oligomers and fibrils. b) 5 μM oligomers. c) 5 μM fibrils. Blue, green, and red fluorescence indicate nuclei, cell membranes and Aβ aggregates, respectively. Scale bar, 20 μm.

13. Aβ42 Fibrils and cell membrane interactions

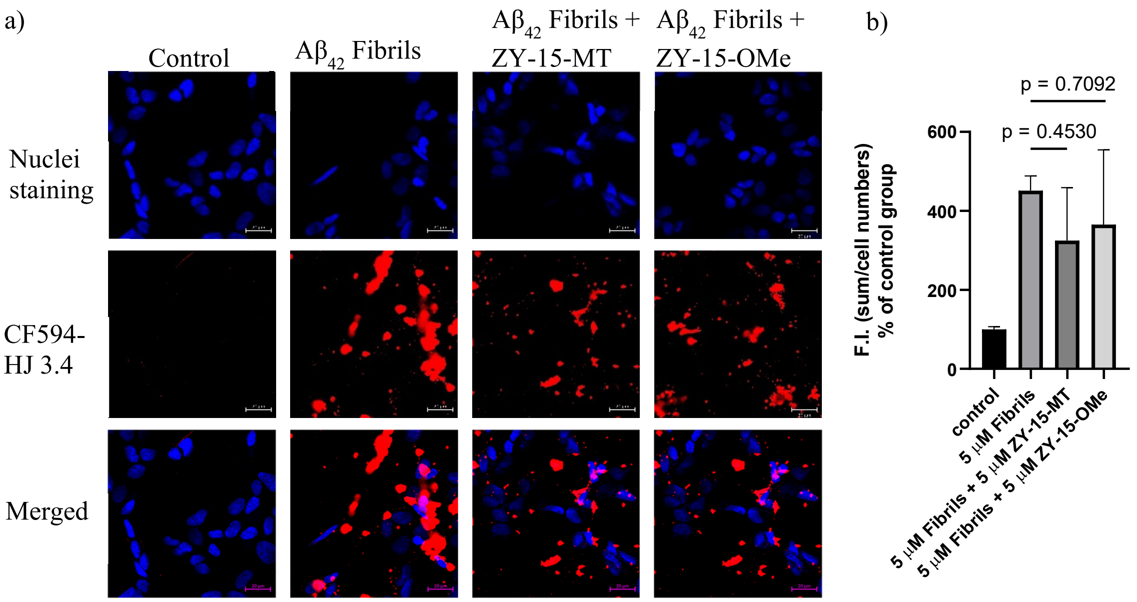

Figure S11. a) SH-SY5Y cells were treated with 5 μM fibrils in the presence or absence of 5 μM ZY-15-MT or 5 μM ZY-15-OMe for 24 h hours before imaging. Red and blue fluorescence indicate the fibrils and nuclei, respectively. Scale bar, 20 μm. b) Three independent experiments were subjected for the statistical analysis and analyzed by one-way ANOVA.

14. NMR spectra

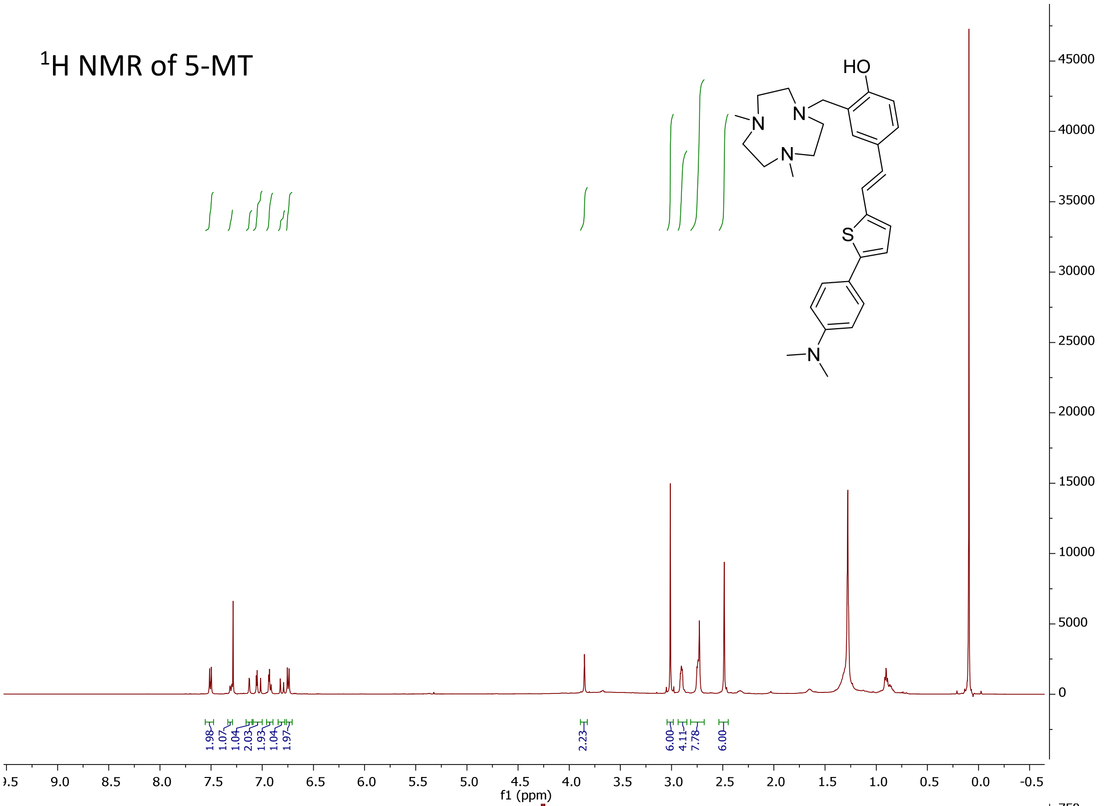

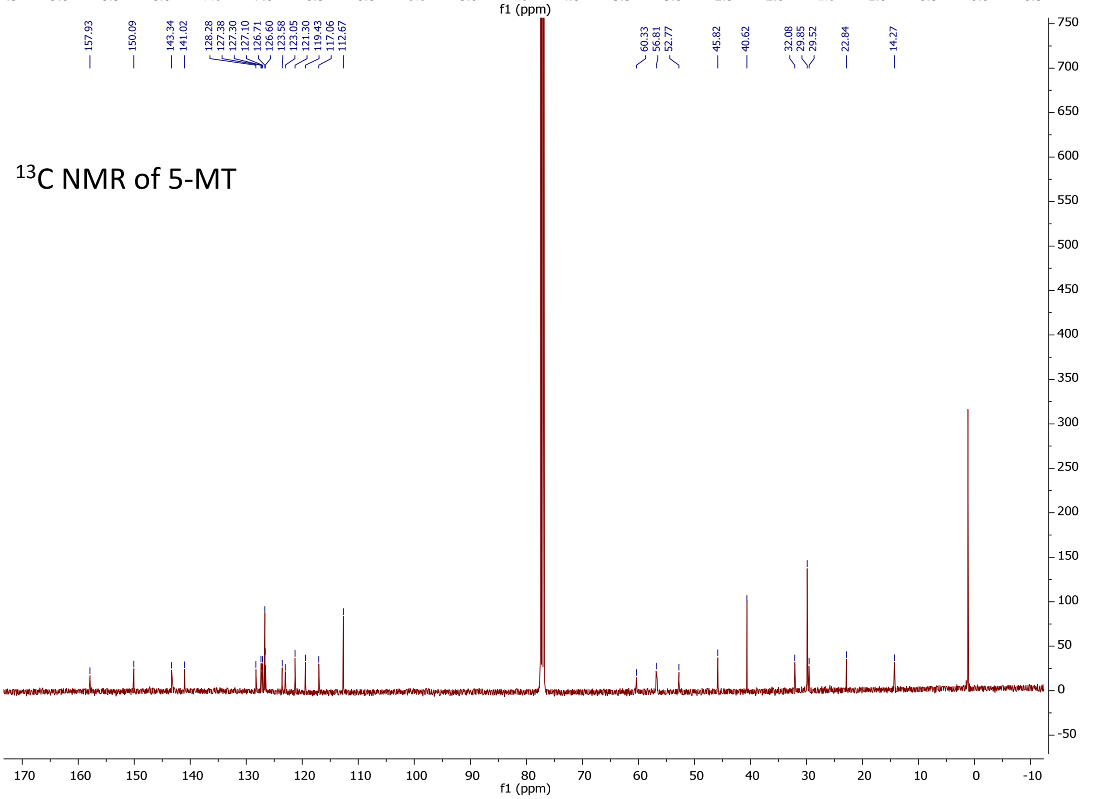

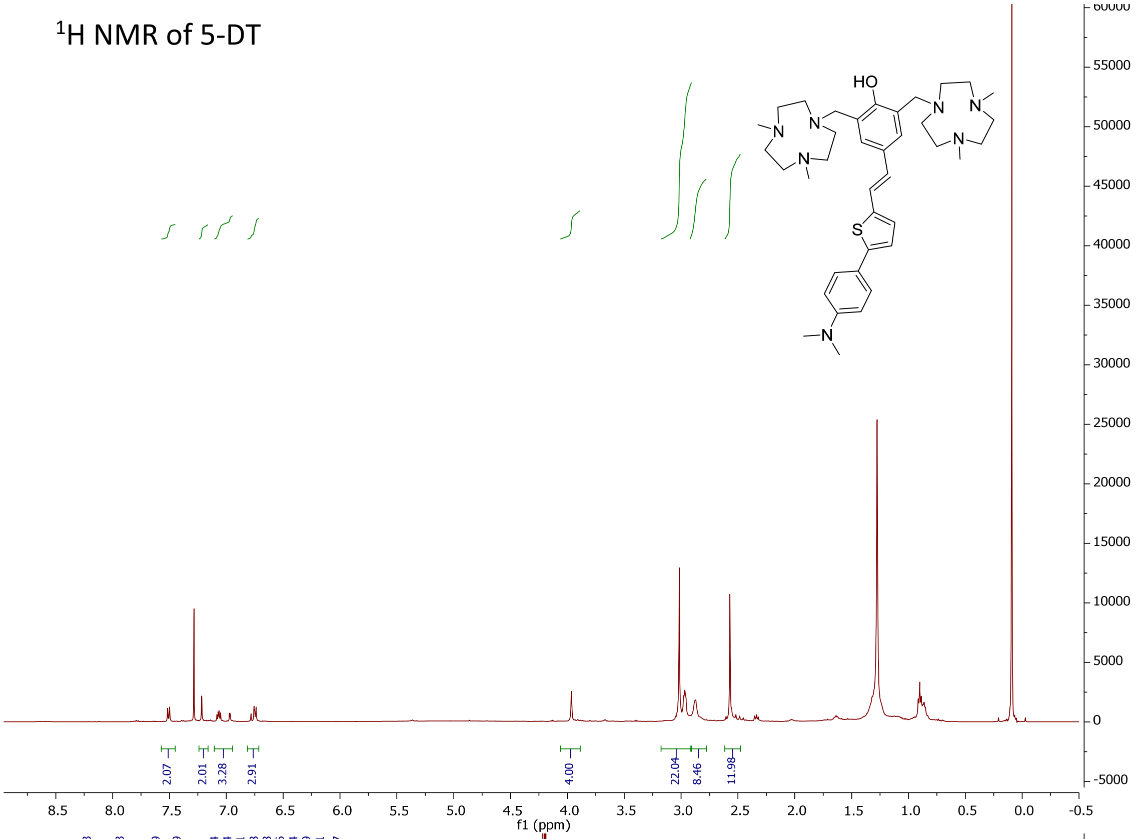

^{1}H NMR of 5-DT
HO
N
N
N
N
N
N
S
N
2.07
2.01
3.28
2.91
4.00
22.04
8.46
11.98
f1 (ppm)

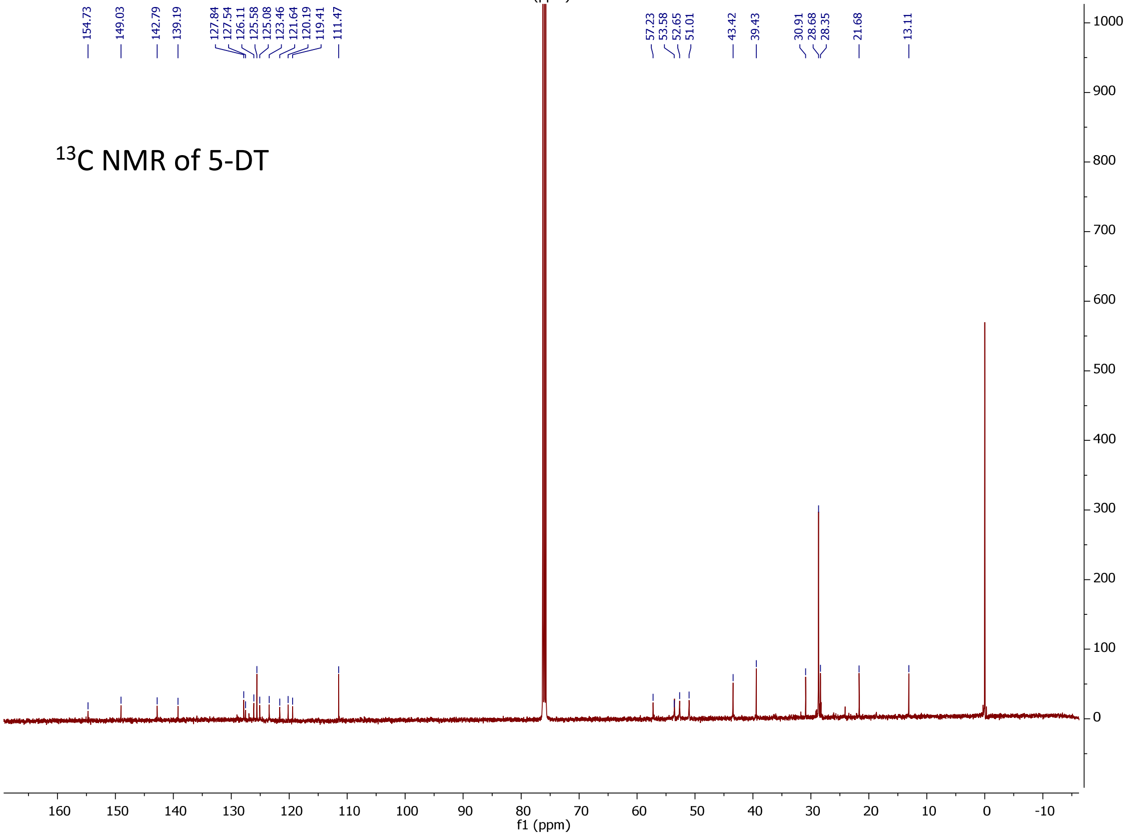

154.73
149.03
142.79
139.19
127.84
127.54
126.11
125.58
125.08
123.46
121.64
120.19
119.41
111.47
57.23
53.58
52.65
51.01
43.42
39.43
30.91
28.68
28.35
21.68
13.11
^{13}C NMR of 5-DT
f1 (ppm)

^{1}H NMR of 5-OMe

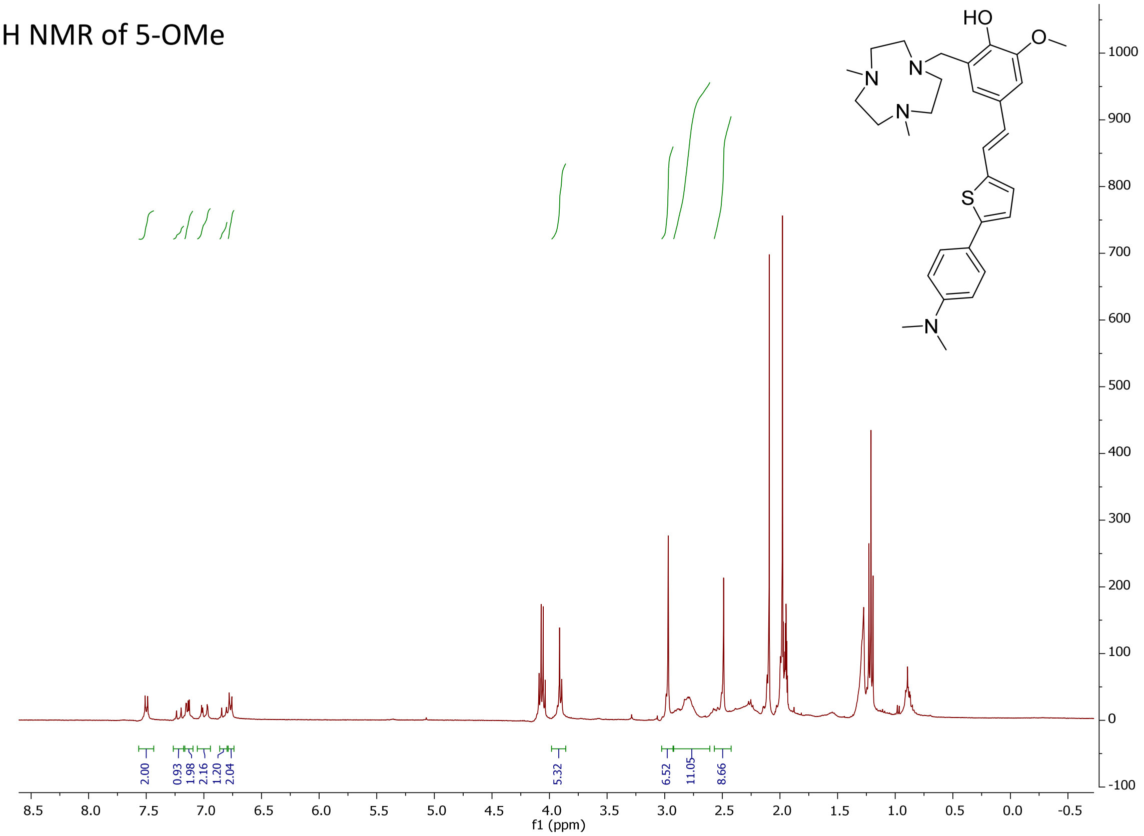

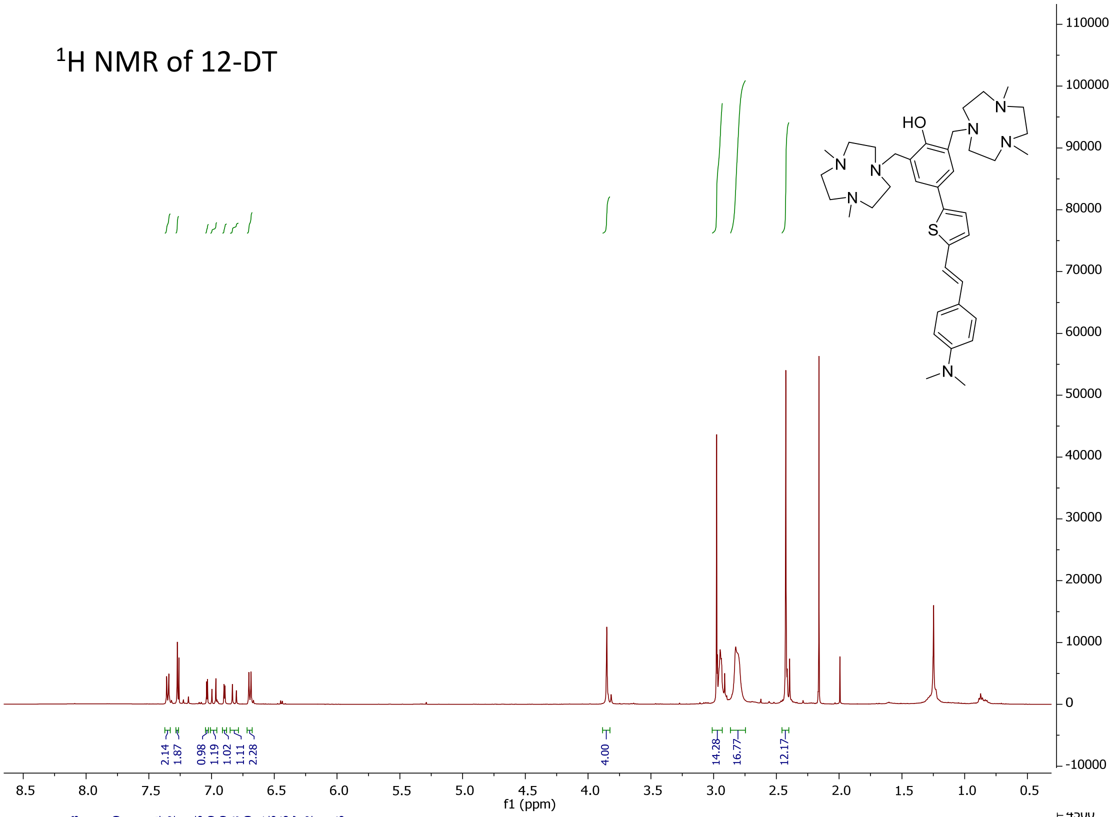

^{1}H NMR of 12-DT
HO
N
N
N
N
N
N
S
N
2.14
1.87
0.98
1.19
1.02
1.11
2.28
4.00
14.28
16.77
12.17
f1 (ppm)

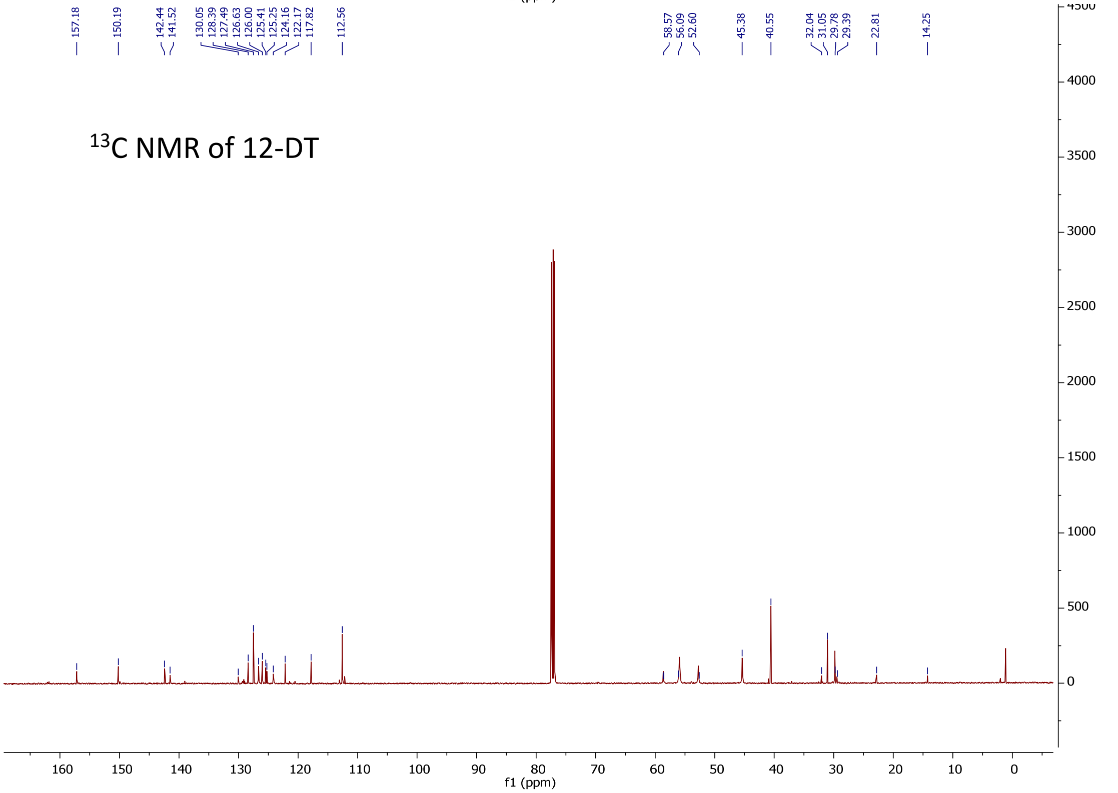

^{13}C NMR of 12-DT
157.18
150.19
142.44
141.52
130.05
128.39
127.49
126.63
126.00
125.41
125.25
124.16
122.17
117.82
112.56
58.57
56.09
52.60
45.38
40.55
32.04
31.05
29.78
29.39
22.81
14.25
f1 (ppm)

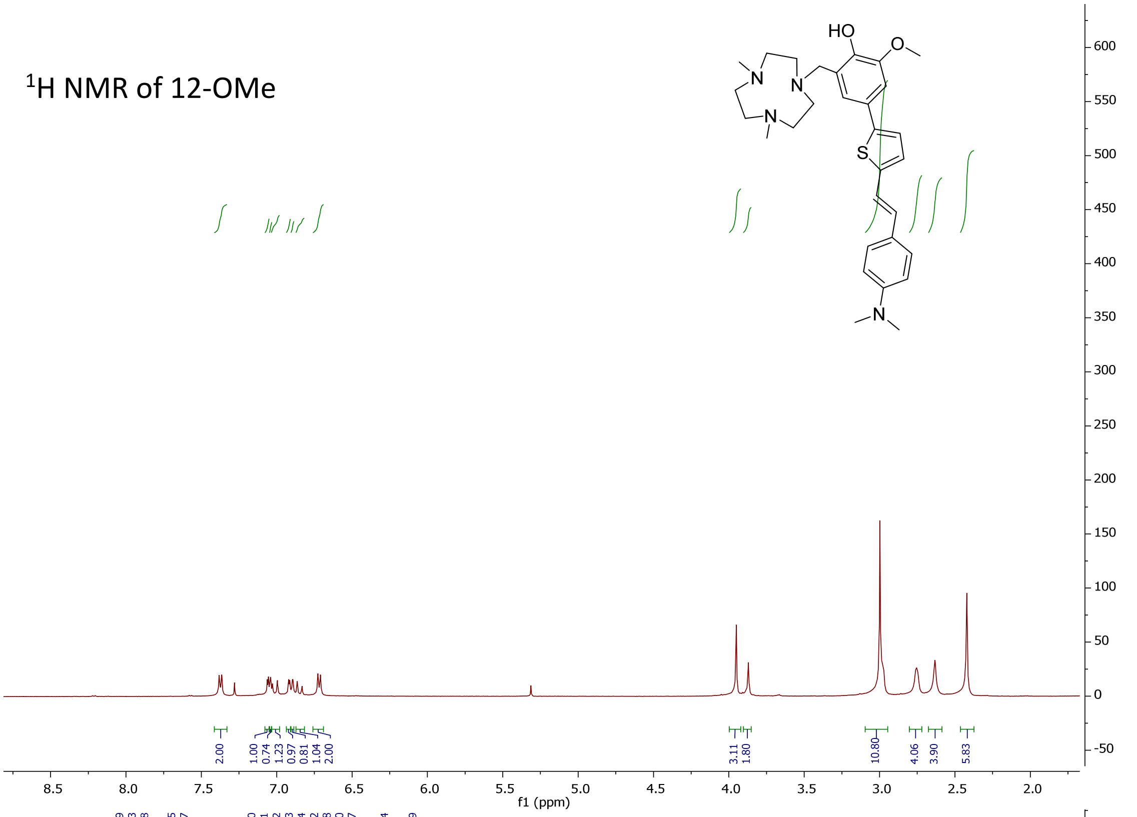

^{1}H NMR of 12-OMe
HO
O
N
N
N
S
N
2.00
1.00
0.74
1.23
0.97
0.81
1.04
2.00
3.11
1.80
10.80
4.06
3.90
5.83
f1 (ppm)

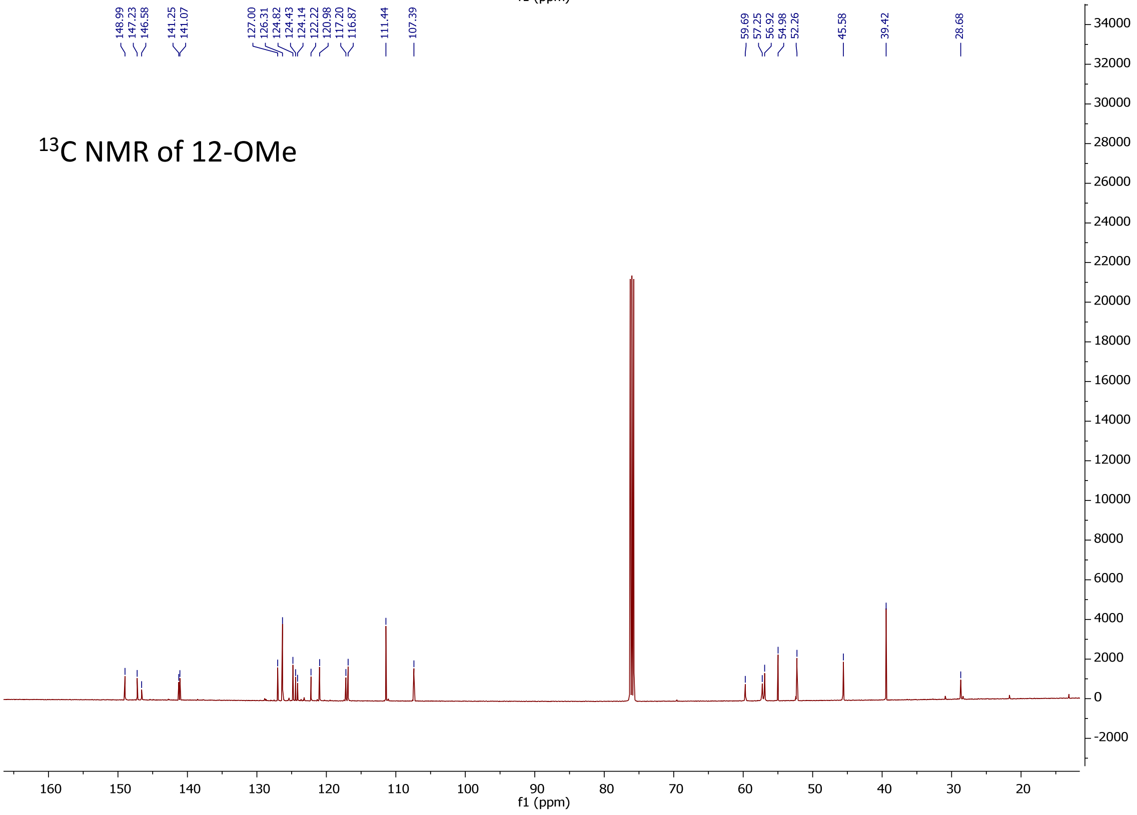

148.99
147.23
146.58
141.25
141.07
127.00
126.31
124.82
124.43
124.14
122.22
120.98
117.20
116.87
111.44
107.39
59.69
57.25
56.92
54.98
52.26
45.58
39.42
28.68
^{13}C NMR of 12-OMe
f1 (ppm)

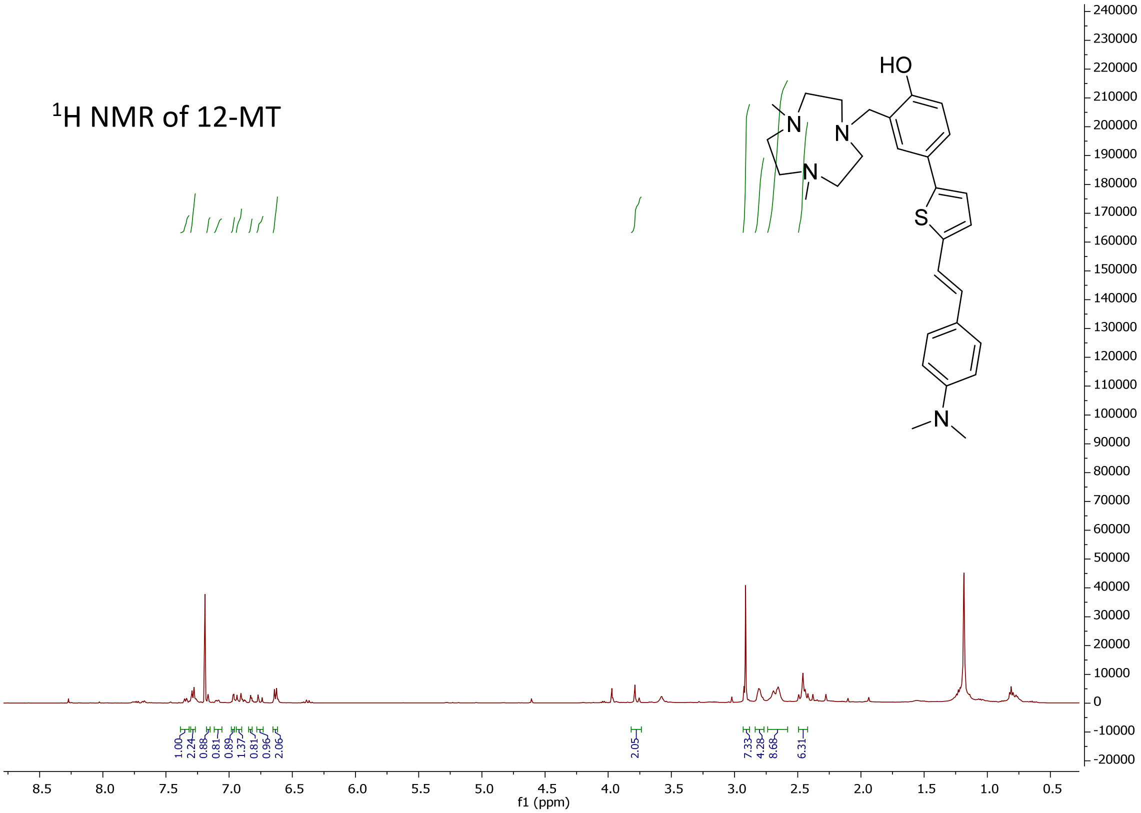

¹H NMR of 12-MT
HO
N
N
N
S
N
1.00
2.24
0.88
0.81
0.89
1.37
0.81
0.96
2.06
2.05
7.33
4.28
8.68
6.31
f1 (ppm)

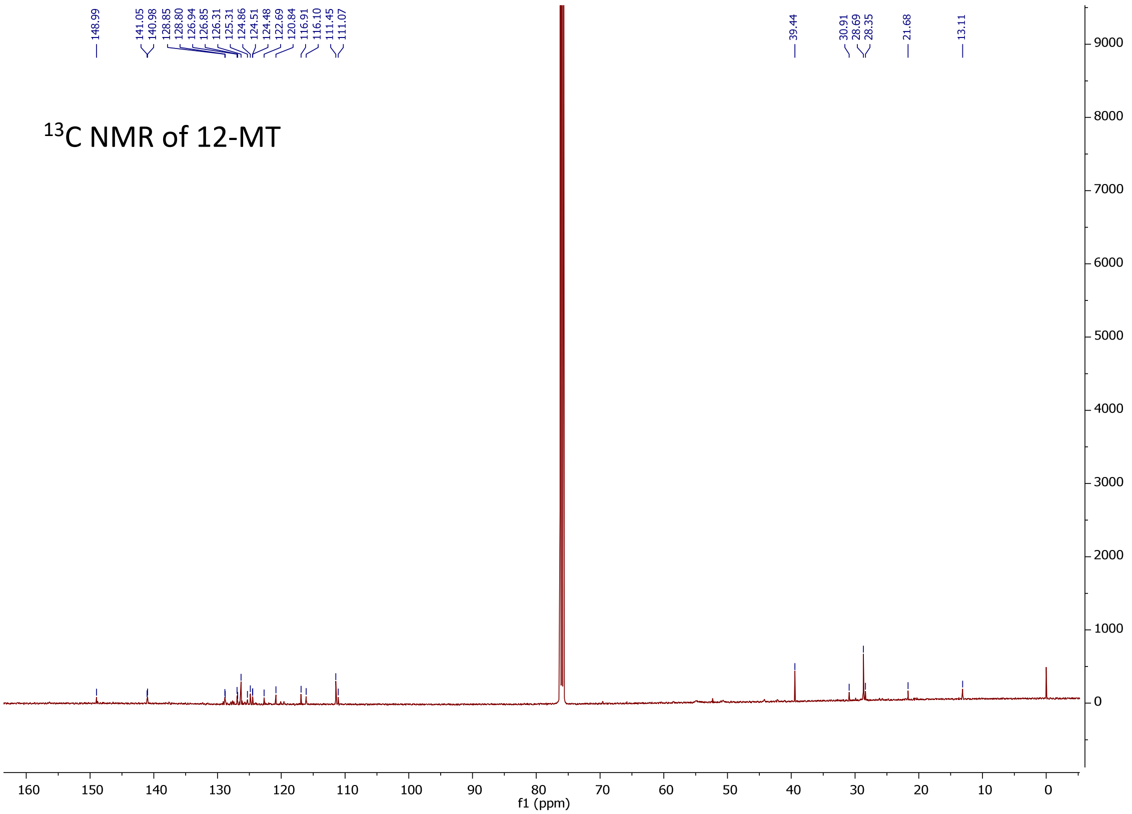

¹³C NMR of 12-MT
148.99
141.05
140.98
128.85
128.80
126.94
126.85
126.31
125.31
124.86
124.51
124.48
122.69
120.84
116.91
116.10
111.45
111.07
39.44
30.91
28.69
28.35
21.68
13.11
f1 (ppm)

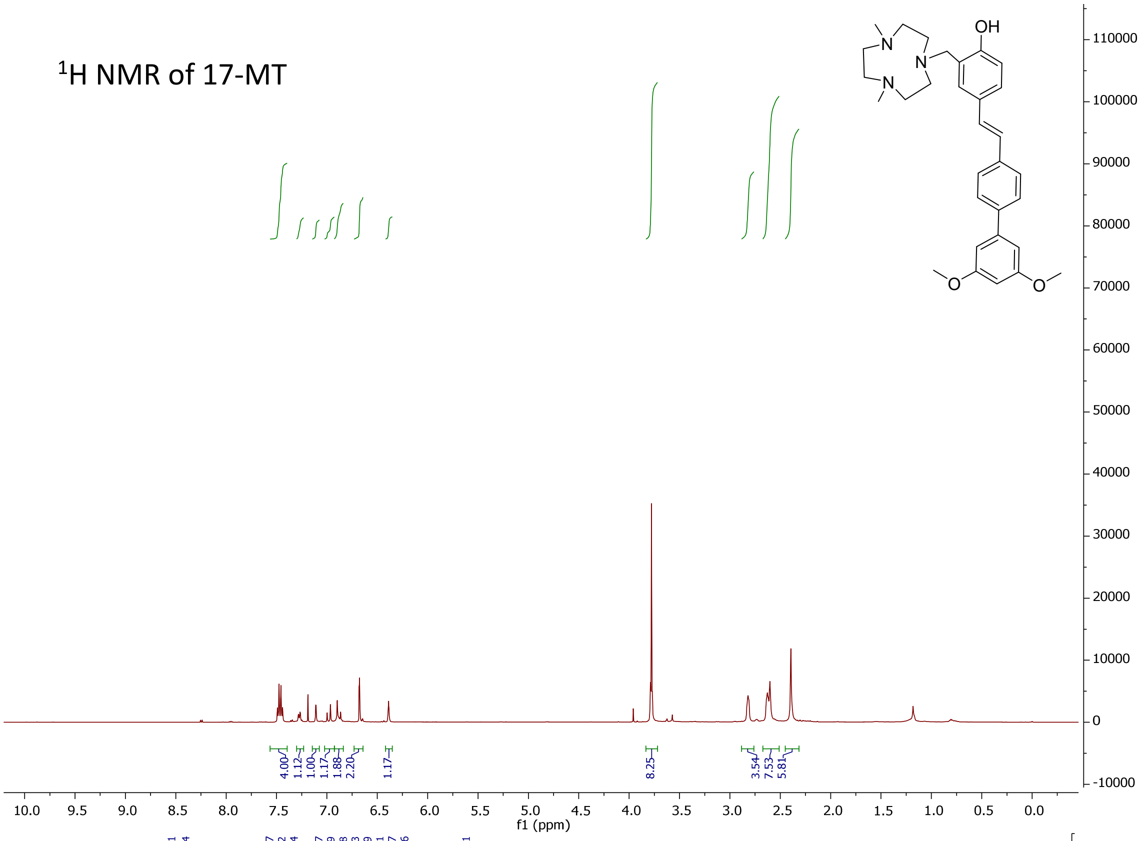

^{1}H NMR of 17-MT
OH
N
N
N
O
O
4.00
1.12
1.00
1.17
1.88
2.20
1.17
8.25
3.54
7.53
5.81
f1 (ppm)

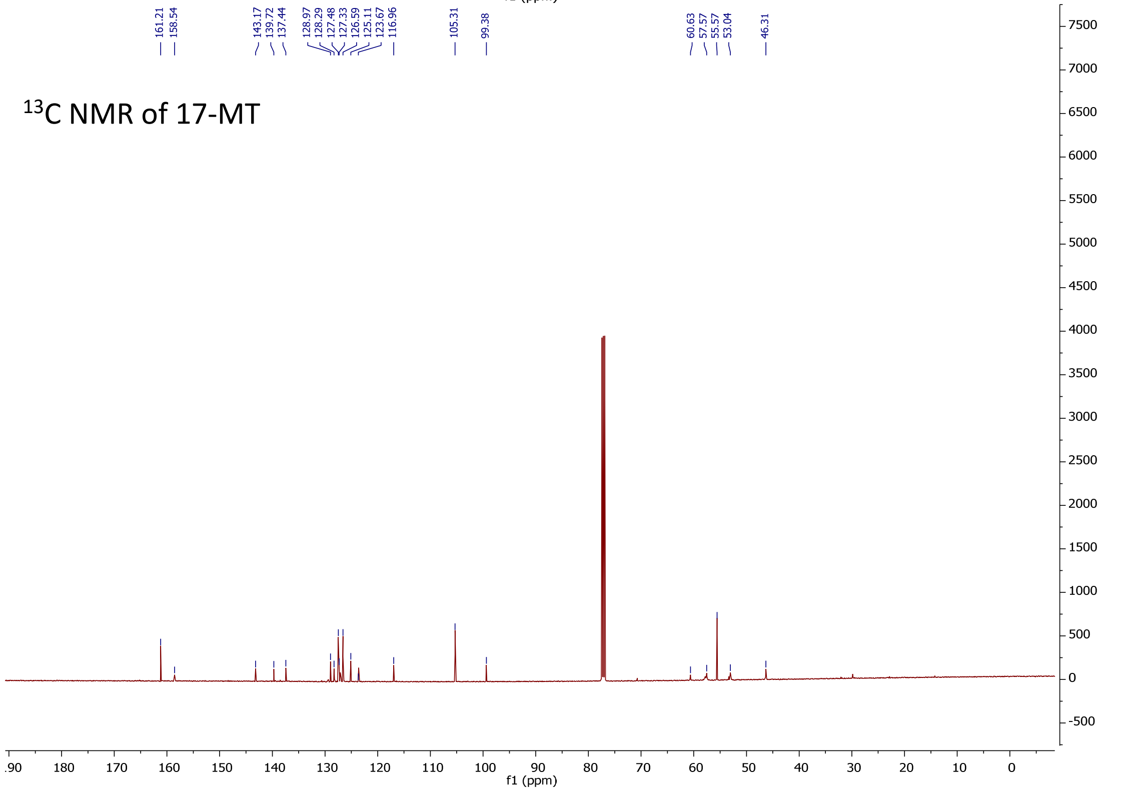

^{13}C NMR of 17-MT
161.21
158.54
143.17
139.72
137.44
128.97
128.29
127.48
127.33
126.59
125.11
123.67
116.96
105.31
99.38
60.63
57.57
55.57
53.04
46.31
f1 (ppm)

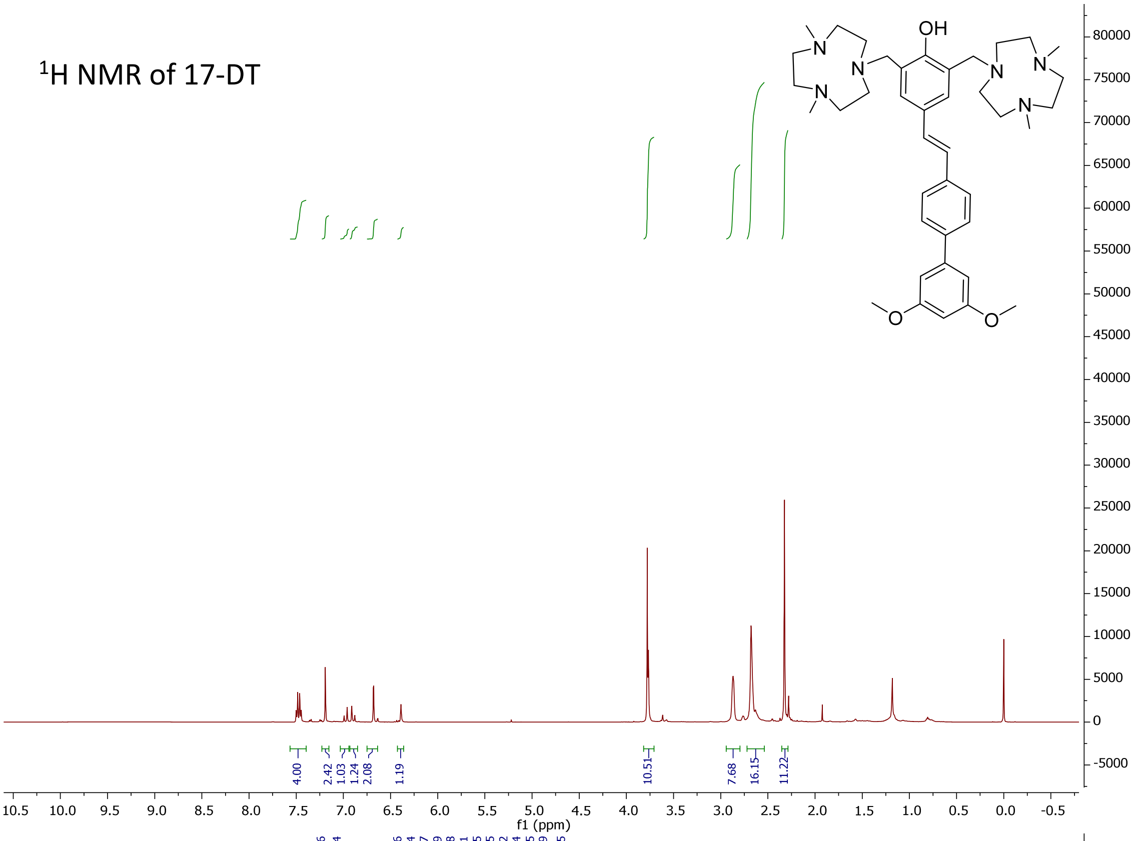

1H NMR of 17-DT
OH
N
4.00
2.42
1.03
1.24
2.08
1.19
10.51
7.68
16.15
11.22
f1 (ppm)

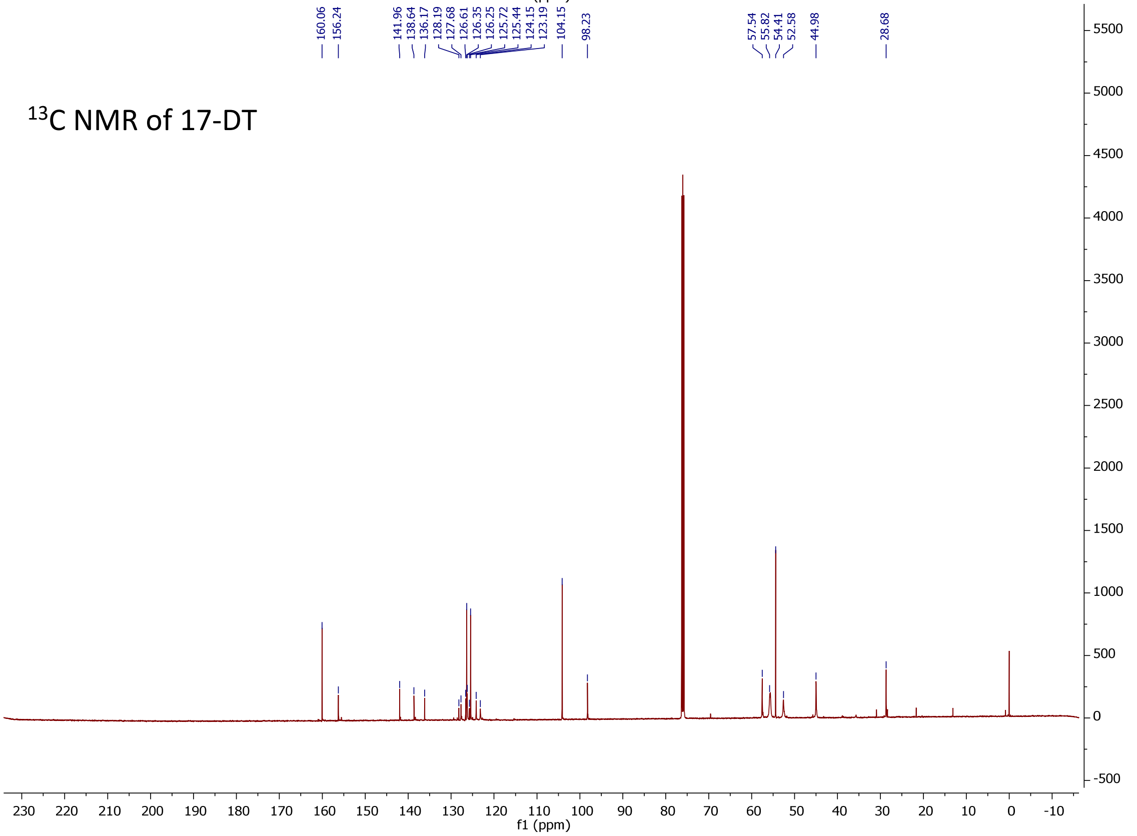

13C NMR of 17-DT
160.06
156.24
141.96
138.64
136.17
128.19
127.68
126.61
126.35
126.25
125.72
125.44
124.15
123.19
104.15
98.23
57.54
55.82
54.41
52.58
44.98
28.68
f1 (ppm)

^{1}H NMR of 17-OMe

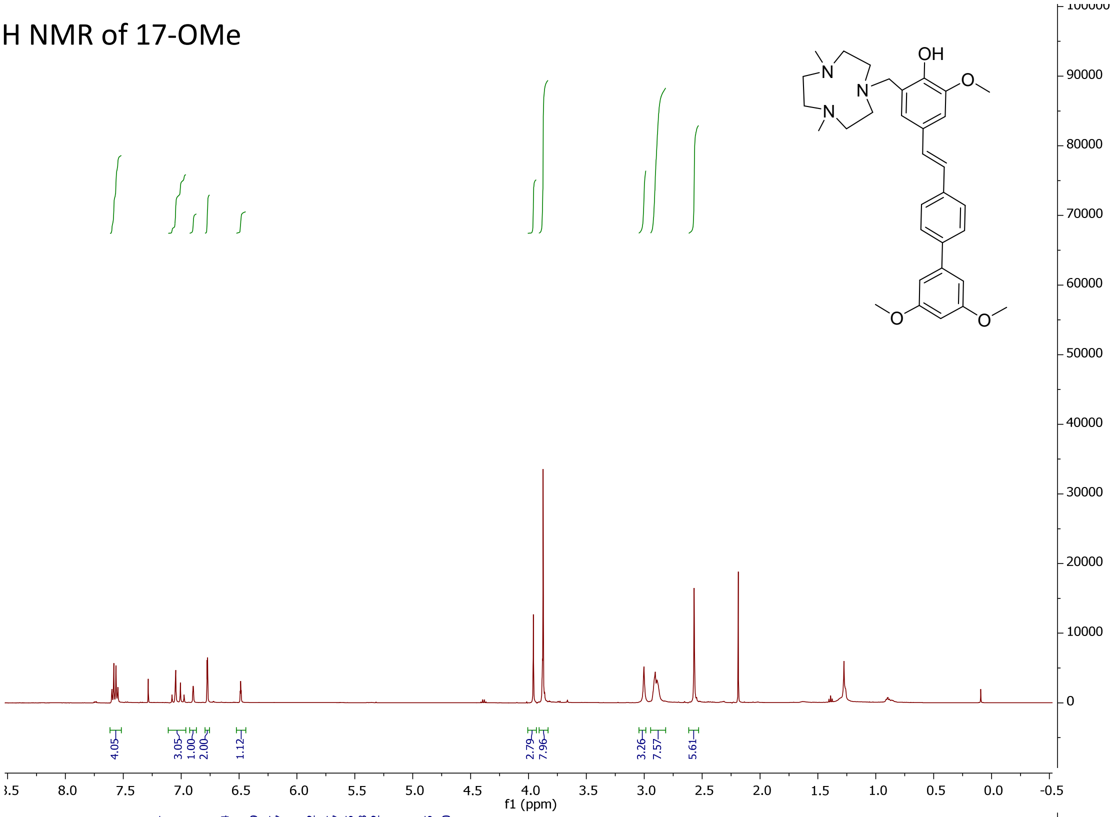

^{13}C NMR of 17-OMe

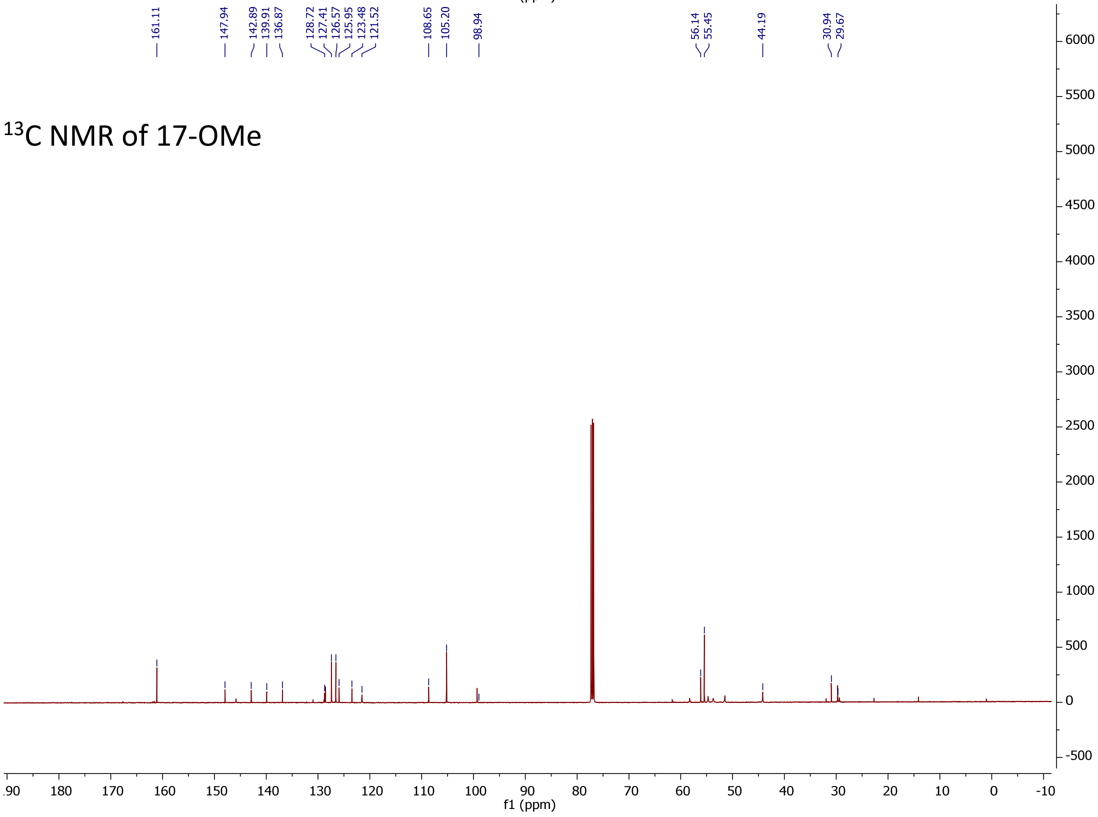

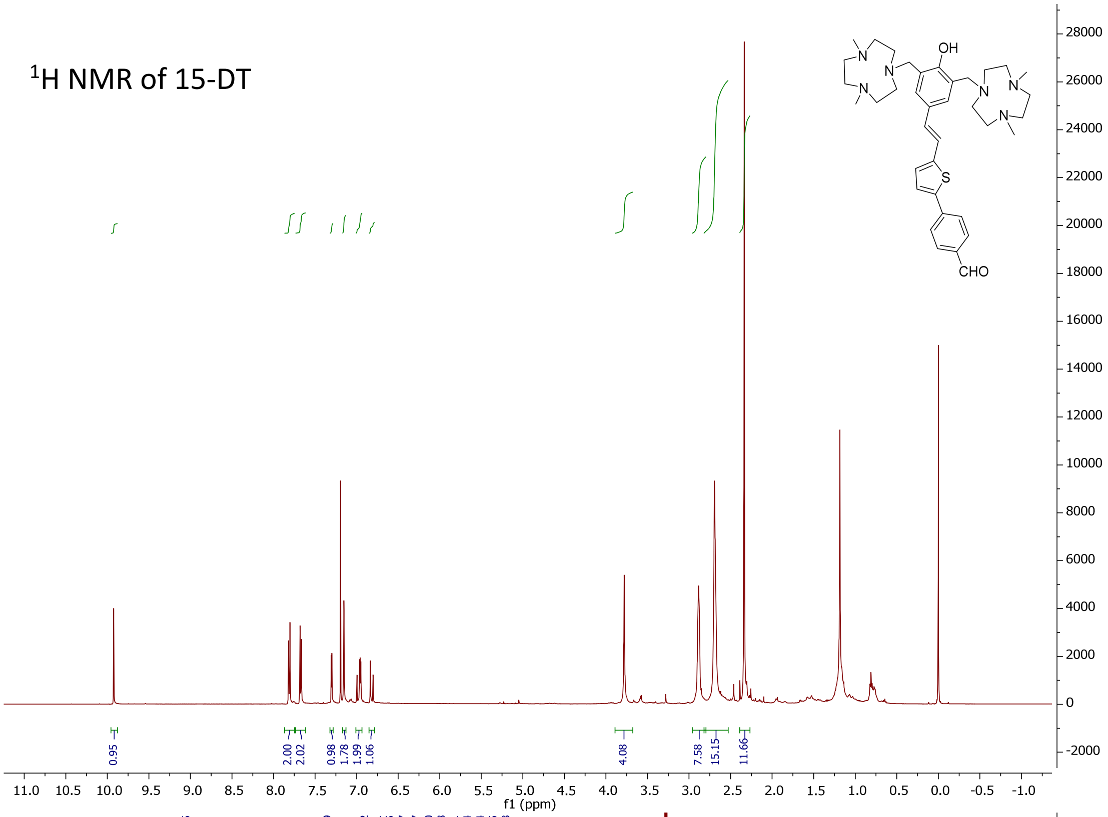

^1H NMR of 15-DT
f1 (ppm)

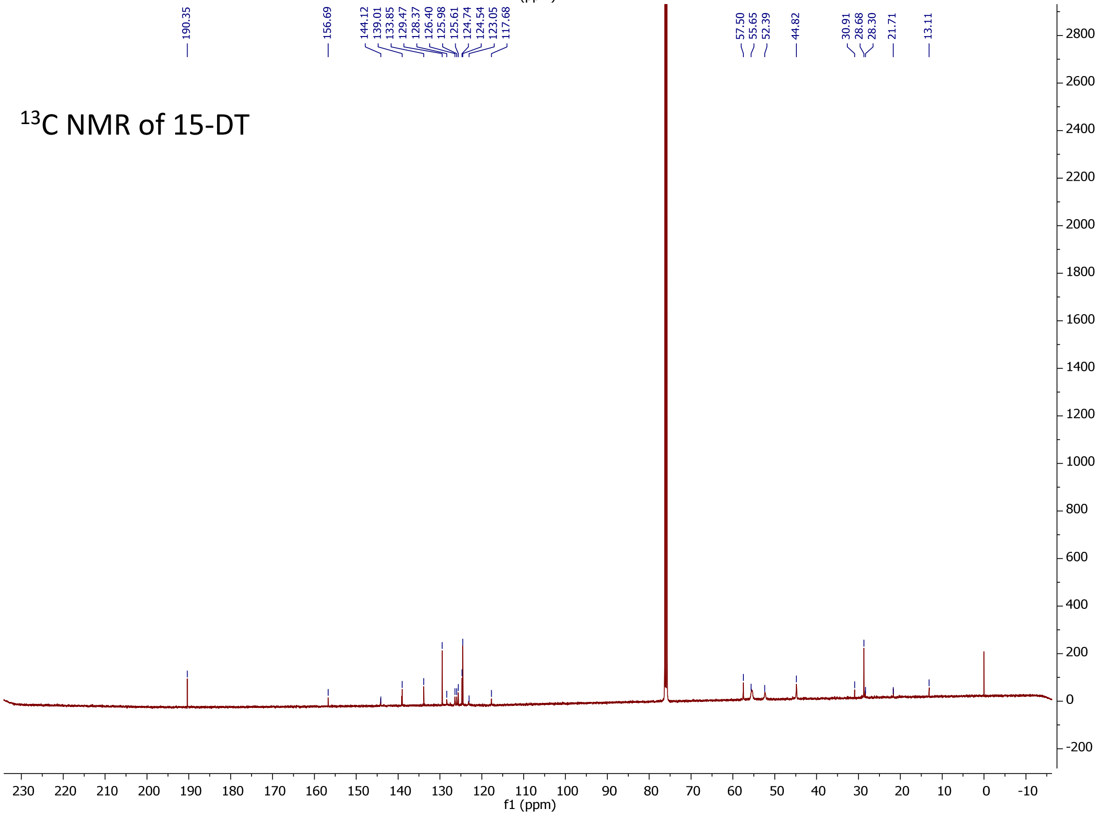

^{13}C NMR of 15-DT
f1 (ppm)

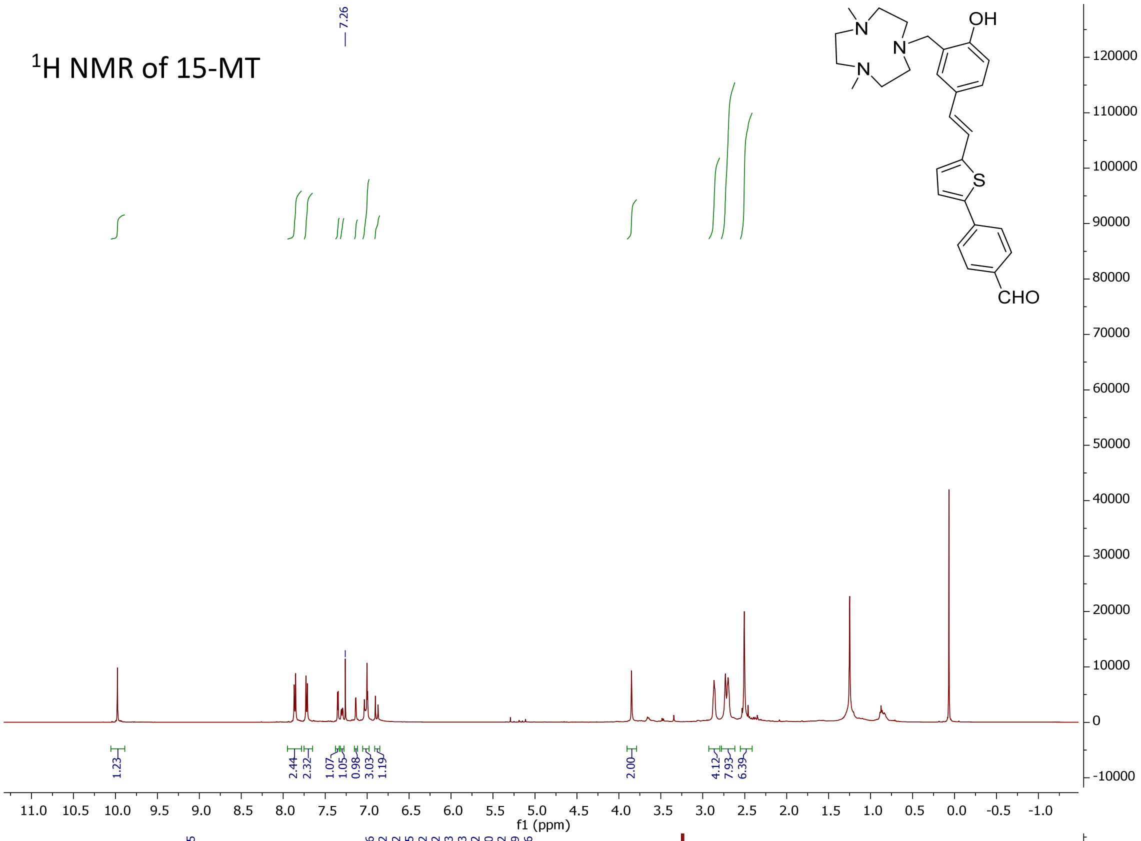

1H NMR of 15-MT
7.26
OH
S
CHO
f1 (ppm)

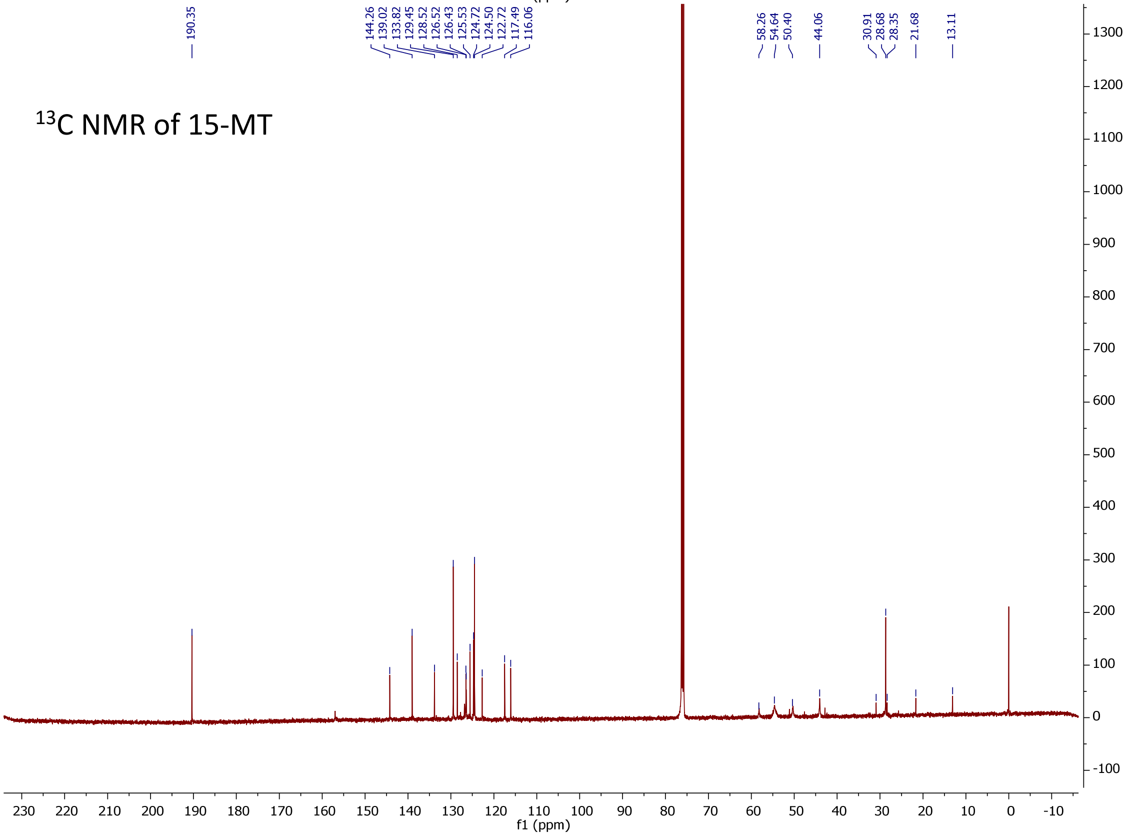

13C NMR of 15-MT
190.35
144.26
139.02
133.82
129.45
128.52
126.52
126.43
125.53
124.72
124.50
122.72
117.49
116.06
58.26
54.64
50.40
44.06
30.91
28.68
28.35
21.68
13.11
f1 (ppm)

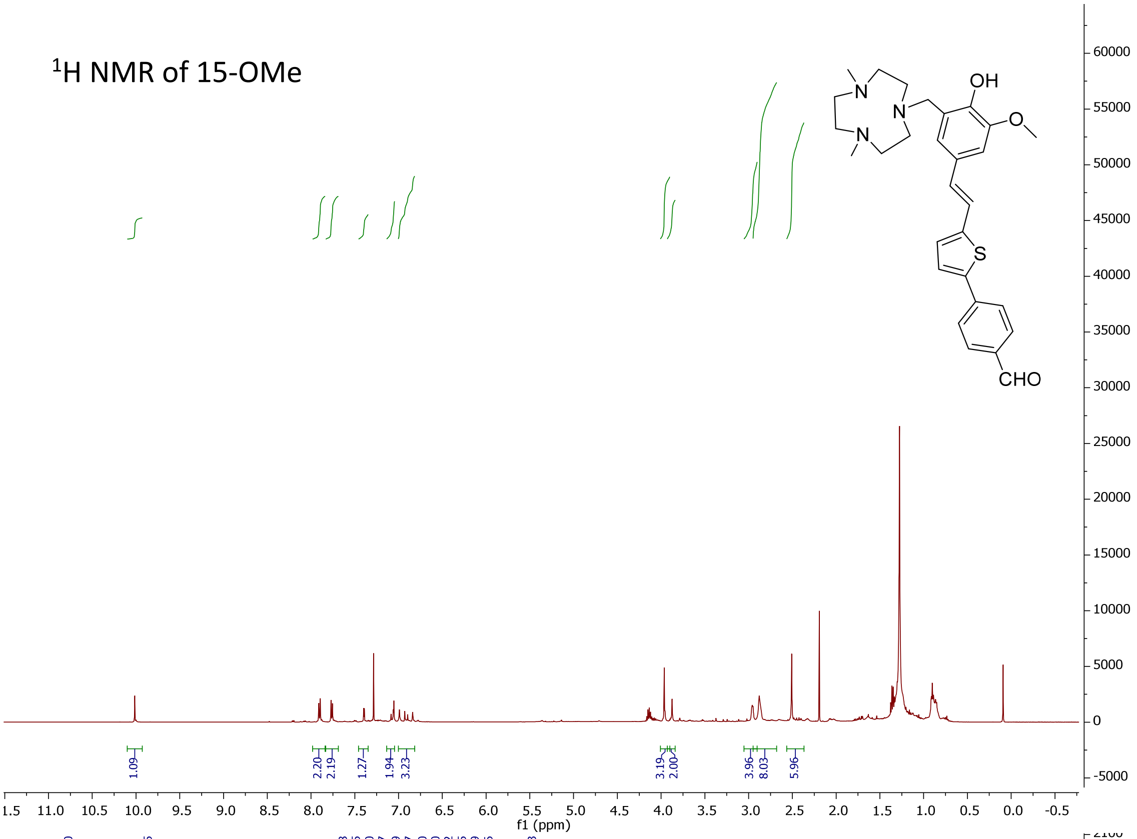
1H NMR of 15-OMe
OH
O
N
N
N
S
CHO
1.09
2.20
2.19
1.27
1.94
3.23
3.19
2.00
3.96
8.03
5.96
f1 (ppm)

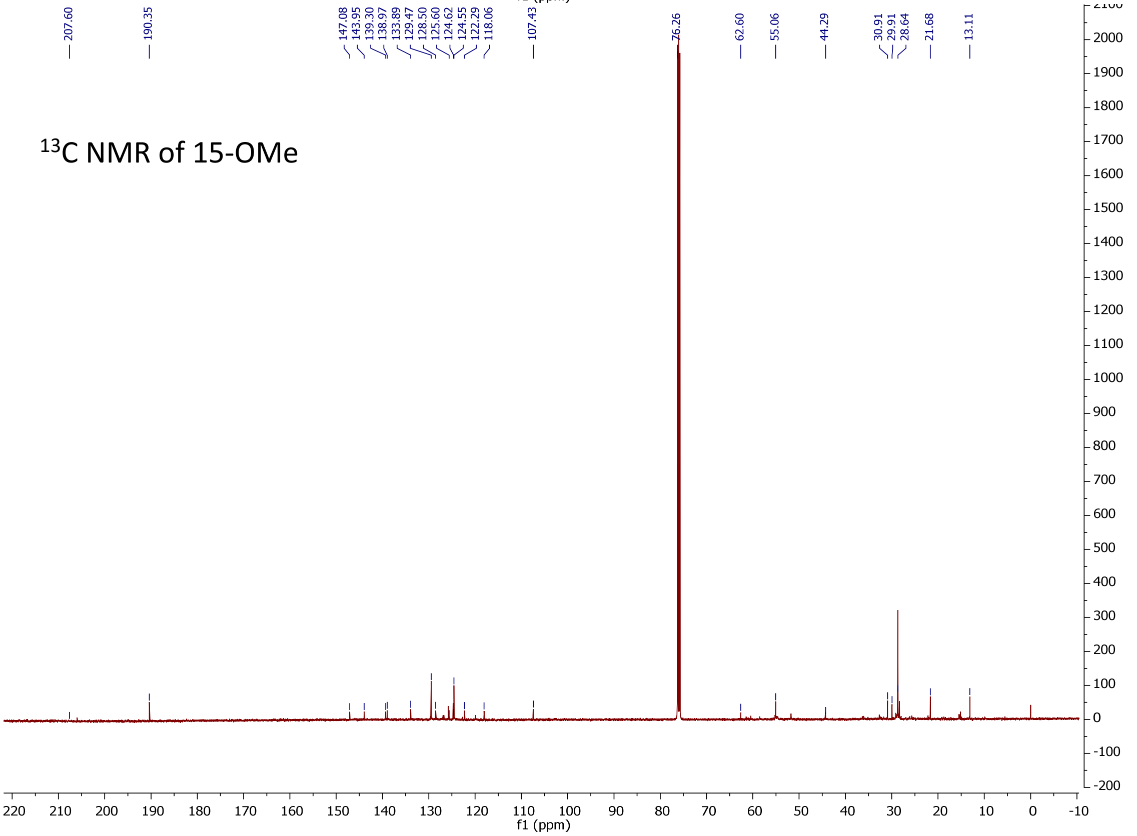
13C NMR of 15-OMe
207.60
190.35
147.08
143.95
139.30
138.97
133.89
129.47
128.50
125.60
124.62
124.55
122.29
118.06
107.43
76.26
62.60
55.06
44.29
30.91
29.91
28.64
21.68
13.11
f1 (ppm)

15. References

1. Sharma, A. K.; Schultz, J. W.; Prior, J. T.; Rath, N. P.; Mirica, L. M., Coordination Chemistry of Bifunctional Chemical Agents Designed for Applications in Cu-64 PET Imaging for Alzheimer's Disease. *Inorg. Chem.* **2017,** *56* (22), 13801-13814.
2. Bero, A. W.; Bauer, A. Q.; Stewart, F. R.; White, B. R.; Cirrito, J. R.; Raichle, M. E.; Culver, J. P.; Holtzman, D. M., Bidirectional Relationship between Functional Connectivity and Amyloid-β Deposition in Mouse Brain. *J. Neurosci.* **2012,** *32* (13), 4334.
3. Dahlgren, K. N.; Manelli, A. M.; Stine, W. B., Jr.; Baker, L. K.; Krafft, G. A.; LaDu, M. J., Oligomeric and fibrillar species of amyloid-beta peptides differentially affect neuronal viability. *J. Biol. Chem.* **2002,** *277* (35), 32046-53.
4. Ran, C. Z.; Xu, X. Y.; Raymond, S. B.; Ferrara, B. J.; Neal, K.; Bacskai, B. J.; Medarova, Z.; Moore, A., Design, Synthesis, and Testing of Difluoroboron-Derivatized Curcumins as Near-Infrared Probes for in Vivo Detection of Amyloid-beta Deposits. *J. Am. Chem. Soc.* **2009,** *131* (42), 15257-15261.
5. Schrödinger, L., The PyMOL Molecular Graphics System, Version 2.3. **2019**.
